# Orientation Selection in Proton-Detected Magic-Angle Spinning Torsion Angle Experiments

**DOI:** 10.1021/acs.jpca.5c07723

**Published:** 2026-03-02

**Authors:** Evgeny Nimerovsky, Marianna Stampolaki, Venus Singh Mithu, Stefan Becker, Loren B. Andreas

**Affiliations:** Department of NMR-Based Structural Biology, Max Planck Institute for Multidisciplinary Sciences, Am Faßberg 11, Göttingen 37077, Germany

## Abstract

Determination of torsion angles via recoupling of backbone HC and HN dipolar interactions is a well-known method in magic-angle spinning NMR spectroscopy. Torsion angle values can be obtained by comparing simulated and experimental signals, either in the frequency or time domains. Typically, all molecular orientations are assumed to have identical detected amplitudes at zero recoupling time. The changes in these amplitudes during the recoupling period define the dipolar coupling values and the torsion angles. Experimentally, however, orientations may exhibit different detected amplitudes due to additional cross-polarization (CP) blocks that connect different spins in multidimensional experiments. We numerically and experimentally investigate how CP blocks bias backbone φ torsion angle determination and propose CP conditions that minimize this effect, thereby improving accuracy. Applying these conditions in pseudo-4D (H)­CANH experiments yields improved agreement of the extracted angles with X-ray crystallographic data for microcrystalline chicken α-spectrin SH3. For the influenza A M2 membrane protein, we identify an unexpected backbone dihedral angle for the I32 residue, which is consistent with TALOS-N predictions but deviates from ideal α-helical transmembrane geometry.

## Graphical abstract

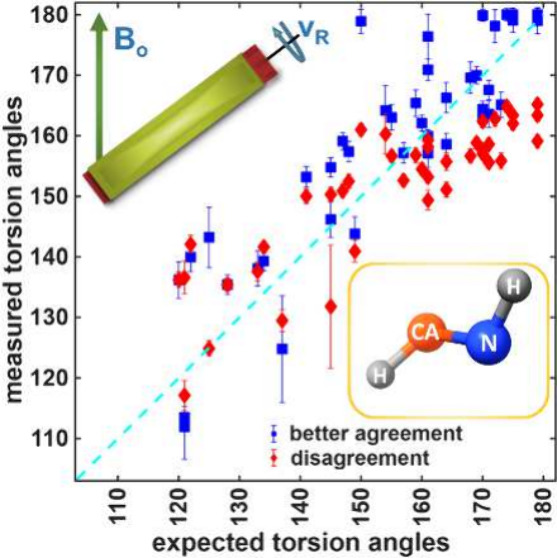

## Introduction

The accurate determination of torsion angle values is an essential factor for the successful calculation and refinement of proton-detected^19,20^ magic angle spinning^21,22^ (MAS) nuclear magnetic resonance (NMR) spectroscopy-based structures of biological macromolecules.^1–18^ Torsion angles can be determined from experiments in which two different dipolar couplings of directly bonded spin pairs are sequentially recoupled.^23–48^ Other experimental methods for determining relative orientation^49^ – such as using recoupled chemical shift anisotropy,^50–56^ measuring distances,^57–66^ observing the evolution of sidebands,^67–69^ or detecting indirect multiple-quantum coherences^70–79^ – have also been proposed. Nevertheless, TALOS-N^80^ remains the most common method for empirical prediction of protein backbone torsion angles.

With recoupling of two backbone H–Cα and H–N dipolar interactions, the ideal torsion angle (TA) signal is considered to depend on two dipolar coupling values, as well as the relative orientation between them – the latter of which defines the backbone torsion angle, ϕ_*H*_. Note that ϕ_*H*_ differs from ϕ by about 60°. Experimentally, dipolar recoupling sequences for TA determination are typically incorporated into multidimensional experiments, which include other dipolar recoupling sequences as building blocks for magnetization transfer.

Historically, such incorporations were first made into carbon-detected experiments using different building blocks. In particular, in the works of Hong et al.^81^ and Huster et al.,^82^ the first building block was cross-polarization (CP)^83^ used to transfer the magnetization from H to C spins. For C–N transfers, the REDOR element^84^ and 90°-pulses were used to create antiphase magnetization, which was then evaluated with simultaneous recoupling of H–Cα and H–N dipolar interactions achieved by either MREV8 ^85^ or FSLG^86^ sequences. The indirect encoding of the ^15^N dimension allowed these experiments to be acquired as 2D spectra, improving the resolution, as demonstrated in later experiments on the M2 protein of influenza A^87^ and for the resonance assignment of a Human α-Defensin, HNP-1.^88^

Another combination of building blocks was proposed by Takegoshi et al.^89^ and Rienstra et al.^8^ In these works, the first CP block^83^ transferred the magnetization from H to N spins. Then, either FSLG2 4̅ 2 ^90^ or T-MREV^35^ was applied to recouple the H–N dipolar interaction. Following this, a second CP block,^91^ and an additional FSLG2 4̅ 2 ^90^/T-MREV^35^ block prior to detection, allowed recoupling of the H–Cα interaction in the same pseudo dimension as the H–N recoupling, thereby introducing a torsion angle dependence. With the development of fast and ultrafast MAS probes, the experiment of Rienstra et al.^8^ was extended from a pseudo-3D experiment to a pseudo-4D experiment^92^ with proton detection,^19,20^ which is the approach used in this article.

While the building blocks are intended only to transfer signal from one nucleus to another, the orientation dependence of the dipolar interaction can result in additional modulation of the final signal. In other words, they can alter the behavior of torsion angle or dipolar coupling signals compared to the ideal ones. The influence of building blocks on the quantitative determination of dipolar couplings was reported in the works of Kurz et al.^93^ and Taware et al.^94^ In the first study, a CP block was used to transfer magnetization from H to C, while the subsequent DIPSHIFT sequence^95,96,97,100^ encoded the measured signal with CH dipolar coupling values. It was found that CP with very short contact times (up to ∼ 100 μs) caused the experimental DIPSHIFT curve to deviate strongly from the theoretically predicted shapes. In the work of Taware et al.,^94^ forward CP blocks (H→C and H→N) were followed by a REDOR-based dipolar recoupling to determine order parameters. The reverse CP steps (C→H and N→H) allowed sequential detection of ^13^C–^1^H and ^15^N–^1^H dipolar-couplings. Through simulation, it was found that reverse CP blocks with very short contact times (up to 160 μs) resulted in faster and deeper dephasing than the standard REDOR curve.

Both of these reports referred to changes in the behavior of dipolar-coupling encoded signals in the presence of CP blocks. Inspired by this work, we investigate how CP blocks influence the quantitative determination of torsion angle (ϕ_*H*_) values in nondeuterated membrane proteins at 55.555 kHz MAS, using pseudo-4D (H)­CANH experiments. We show that CP blocks reduce the torsion-angle sensitivity of TA curves, thereby lowering the accuracy of angle extraction. Using numerical simulations and experiments, we identify CP conditions that minimize this effect and enable more accurate torsion angle measurements.

## Results and Discussion

### Orientation Selection of N–H CP

To recouple H–Cα and H–N dipolar interactions, an improved version of the MODERN pulse sequence,^43^ called pMODERN, was used. The MODERN and pMODERN basis elements, together with a comparison of their simulated and experimental efficiencies, are shown in Figure S1 of the Supporting Information (SI). While pMODERN requires approximately 1.1 times higher RF-field strength and has a smaller dipolar scaling factor^33^ compared to MODERN, it exhibits reduced sensitivity to RF-field missettings overall. First, we quantify the impact of ramped CP blocks on TA signals by comparing simulated and experimental data acquired with and without CP blocks. The ideal case assumes orientation-independent behavior for all building blocks in the pulse sequence, which should be experimentally accessible via J-coupling-based transfers.

The ideal TA signal can be described by the following equation

$${\mathrm{TA}}_{ideal}\left(t_{1,\mathrm{rec}};t_{2,\mathrm{rec}}\right)=\int \mathrm{d\Omega s}\left(\Omega ,\nu_{D,\mathrm{HC}},t_{1,\mathrm{rec}}\right)s\left(\phi_{H},\Omega ,\nu_{D,\mathrm{HN}},t_{2,\mathrm{rec}}\right)$$ 1

where the integration over orientation (Ω) represents powder averaging using Euler angles, (α, β, γ).^98^ The functions s­(Ω, ν_D, HC_, *t*_1, *rec*_) and s­(Ω, ν_D, HN_, ϕ_*H*_, *t*_2, *rec*_) are orientation-dependent signals at the end of the pMODERN recoupling sequence with recoupling times *t*_1, *rec*_ and *t*_2, *rec*_, respectively. The ν_D, HC_ and ν_D, HN_ represent the HC and HN dipolar coupling values. While the response of each molecular orientation to the recoupling sequence varies, eq 1 assumes uniform detected amplitudes at zero recoupling time (*t*_1, rec_ = *t*_2, rec_ = 0). The initial shape of the TA curve is primarily influenced by the ν_D, HC_ and ν_D, HN_ values, however, at longer recoupling times, the TA curve is also influenced by the ϕ_*H*_ value. The NMR-determined angles can theoretically be either positive or negative. Fortunately, ϕ_*H*_ is negative for both β sheet and alpha helical secondary structure, and we can therefore safely assume negative values. In the following, we consider only the magnitude of ϕ_*H*_ (|ϕ_*H*_|), since the TA curve cannot be used to determine the sign. With the presence of CP blocks, eq 1 is modified as follows

$$TA\left(t_{1,\mathrm{rec}};t_{2,\mathrm{rec}}\right)=\int \mathrm{d\Omega}\xi \left(\Omega ,\Omega^{PAS},{CP}_{par}\right)s\times (\Omega ,\nu_{D,\mathrm{HC}},t_{1,\mathrm{rec}})s\left(\phi_{H},\Omega ,\nu_{D,\mathrm{HN}},t_{2,\mathrm{rec}}\right)$$ 2

where ξ­(Ω, Ω^*PAS*^, *CP*_*par*_) represents the detected amplitude of an orientation at *t*_1, rec_ = *t*_2, rec_ = 0. *CP*_*par*_ denotes the set of all CP parameters (the RF-field strengths, duration of the contact times, the shapes) used in the CP blocks to connect the initial and final spin states. Ω^*PAS*^ refers to Ω-dependent orientation of the dipolar interactions^98^ that are relevant to the CP transfer steps.

To confirm the influence of CP blocks by simulation, we consider a three-spin system, H1–N1–N2–H2 (schematically shown in the inset of Figure 1D), in which the Hα–Nα and Hβ–Nβ dipolar interactions are recoupled. The initial and final spin operators are Ha and Hb, respectively, and the signal follows the transfer pathway H1→N1→N2→H2. In the simulations, H1→N1 and N2→H2 CP transfers were taken into account, while for the N1→N2 transfer, a 100% (or uniform) transfer efficiency was assumed. In this case, the simulated system can be simplified by considering an H1–N1/N2-H2 system with a projection angle,^99^ ϕ_*proj*_. Note that this is distinct from the case of the backbone ϕ_*H*_ angle, which spans projection angles, ϕ_*proj*_, of about 49–169 degrees, since angles such as θ_*HNCa*_ are not 90° as detailed in eq S5 of ref 92).

**Figure 1:**
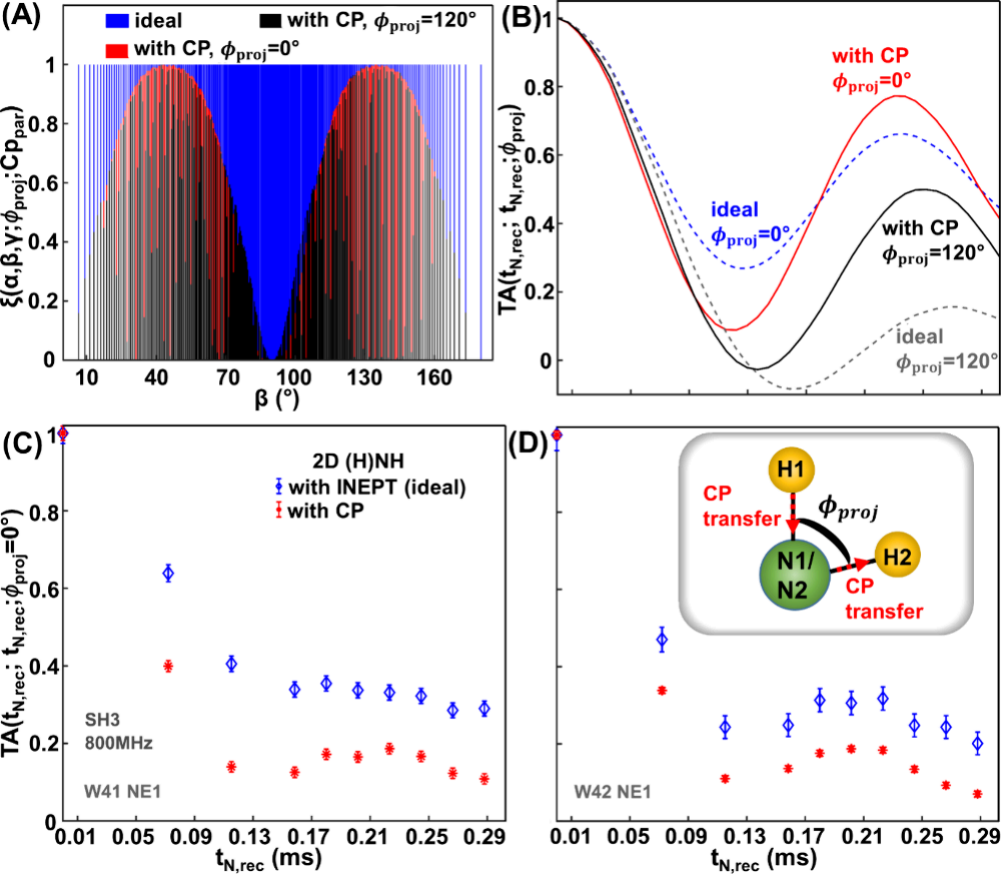
Simulated Ha-Na/Nb-Hb spin system (A)-(B) and experimental curves obtained from 2D (H)­NH spectra of SH3 with different pMODERN recoupling times (C)-(D). The ^15^N–^1^H dipolar coupling values were 10 kHz. The pMODERN sequence was applied twice in succession to recouple the ^15^N–^1^H dipolar coupling. The inset in (D) schematically depicts the three-spin system composed of H1–N1/N2-H2. (A) The amplitude of an individual orientation as a function of angle β in ideal case (blue) and in the case where ramped CP blocks are used for H1→N1/N2 and N1/N2→H2 transfers, with projection angle values of ϕ_*proj*_ = 0° (red) and ϕ_*proj*_ = 120° (black). (B) Simulated curves for two different torsion angle values, 0° and 120° in the ideal case (dashed lines) and with CP blocks (solid lines). (C) and (D) Experimental curves of side chain N–H moieties obtained using refocused INEPT (blue diamonds) and ramped CP (red stars) for the ^1^H→^15^N and ^15^N→^1^H transfers.

Figure 1A shows the distribution of ξ values (in eq 2) as a function of β for two different projection angle values (0° and 120°). These distributions are also shown in separate panels in Figure S3 (A-C). In the ideal case (blue), ξ is uniformly distributed and does not depend on the torsion angle. However, ξ becomes dependent on β and ϕ_*proj*_ (red and black) when two ramped CP blocks are used to connect H1 with N1/N2 and N1/N2 with H2.

With the recoupling of H1–N1/N2 and N1/N2–H2 dipolar interactions, a strong deviation is observed between the ideal TA curves (Figure 1B, blue and gray dashed lines) and the TA curves with dipolar-based CP blocks (Figure 1B, red and black solid lines). Moreover, the use of the CP blocks to connect different spins reduces the sensitivity of the TA curve to ϕ_*proj*_. The separation between the TA curves, represented here by the extreme cases of ϕ_*proj*_ = 0° and ϕ_*proj*_ = 120°, is reduced by around a factor of 2 when CP is used (solid lines).

The same behavior can be observed in experimental curves, as shown in Figure 1C–D for two amino acids in SH3. The pMODERN sequence was incorporated in 2D (H)­NH experiments in such a way that the same H–N dipolar coupling was twice sequentially recoupled (the sequences are shown in Figure S3D-E in the SI). In this case, the experimental signals behave as a TA curve with ϕ_*proj*_ = 0°, which mirrors the simulated conditions in Figure 1B. Cleary, CP blocks have a significant influence on TA curves and reduce the accuracy of torsion angle determination compared to ideal TA signals (blue diamonds). Figure S4 in the SI presents additional experimental curves for seven amino acid residues of SH3, which support the same conclusion.

### Evaluating cCP Conditions

While J-transfer-based blocks may potentially be more effective for torsion angle determination, dipolar-transfer-based CP blocks offer much higher transfer efficiency than refocused INEPT for nondeuterated samples with short relaxation times.^101^ Therefore, we sought a CP condition with tolerable orientation selection, which also allows measurement of dipolar couplings (order parameters).

Since the number of CP blocks used in the experiment affects the orientation selection, we need to use the 3D (H)­CANH sequence^19,102^ (Figure 2A) as the basis for trial CP conditions. Both the dipolar (Dip) and torsion angle (TA) curves were acquired using pMODERN sequences. To acquire Dip curves, pMODERN is applied either for CH or NH recoupling in separate experiments, whereas for TA measurements, CH and NH dipolar couplings are both recoupled within the same experiment.

**Figure 2:**
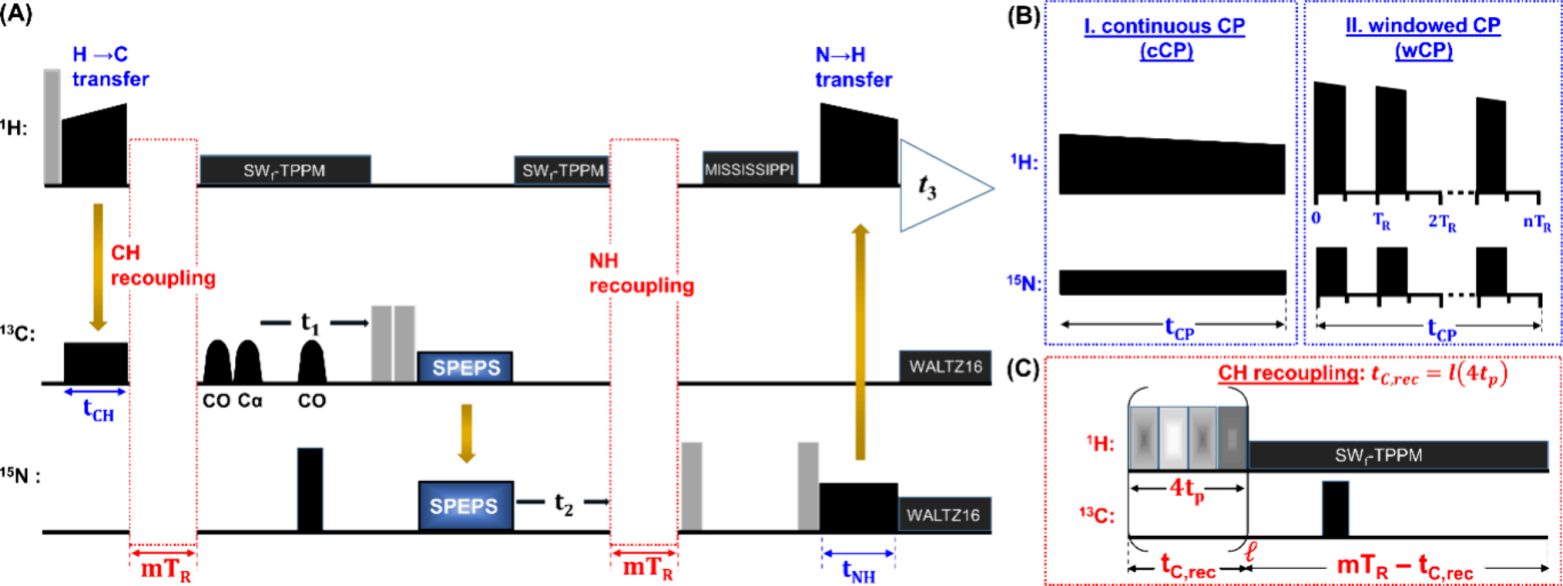
Pulse sequence for torsion angle and dipolar coupling measurements. (A) 3D (H)­CANH with integrated pMODERN sequence for CH recoupling (red boxes, expansion in (C)). The π/2-pulses are indicated by light rectangles, and π-pulses by black rectangles. Ramped CP blocks^83^ effect ^1^H to ^13^C and ^15^N to ^1^H transfers. During the indirect dimension (*t*_1_) and acquisition (*t*_2_), SW_f_-TPPM decoupling^105^ is applied. π-pulses in the middle of *t*_1_ and *t*_2_ are used to decouble carbon–nitrogen interactions. During acquisition, WALTZ16 decoupling^106^ is applied on nitrogen and carbon channels. REBURP soft selective pulses^107^ are applied on the carbon channel to select ^13^CA spins. For ^13^C→^15^N transfers, the SPEPS block^104^ is used. In the SPEPS block, a pair of π/2-pulses is applied before the transfer in order to invert the real part of the signal for even increments of the indirect dimension. (B) Ramped CP blocks with continuous (cCP) and windowed (wCP) shapes.^103^ (C) Constant-time^35^ pMODERN sequence. The basic pMODERN sequence consists of four pulses with phases of 124°, 0°, 123°, 180°. The duration (*t*_*p*_) and RF-field strength of each pulse are 0.2*T*_*R*_ (*T*_*R*_ = 1/ν_*R*_, where ν_*R*_ is the MAS rate in kHz) and 2.75ν_*R*_, respectively. The total pMODERN recoupling time is defined as *t*_*C*, *rec*_ = *l*(4*t*_*p*_). For NH recoupling, a doubled pMODERN recoupling time (*t*_*N*, *rec*_ = 2*t*_*C*, *rec*_) was applied in all experiments and simulations.

Figure 2 shows the pulse sequence for torsion angle and dipolar coupling measurement. CP shapes for ^1^H→^13^C and ^15^N→^1^H transfers can be either continuous (cCP) or windowed (wCP), as illustrated in Figure 2B. While, both shapes offer similar transfer efficiency when FLAN conditions are satisfied,^103^ they have different effects on the TA curves (further details regarding FLAN conditions in the SI, Figure S5). The SPEPS block^104^ was implemented for the ^13^C→^15^N transfer, with a duration of 2.88 ms. The pMODERN sequence for CH (NH) recoupling is depicted in Figure 2C.

First, we numerically and experimentally investigated the influence of zero-quantum and double-quantum CP conditions^108,109^ – on Dip and TA curves. We considered a spin system representing the backbone of an amino acid, with four protons (Hα, two Hβ and ^N^H spins) dipolar-coupled to a Cα spin. The Cα spin is dipolar-coupled to an N spin, which in turn is dipolar-coupled to the amide proton, forming the coupling path ^N^H/Hα/(Hβ)_2_→Cα→N→^N^H.

Figure 3 shows experimental (HCAN)H Dip (A-B) and TA (C–D) curves for different CP cCP conditions. For CH recoupling (Figure 3A), changing the ^15^N→^1^H CP condition from 0.75ν_*R*_/ 1.75ν_*R*_ RF-field strengths to 0.25ν_*R*_/ 1.25ν_*R*_ and 0.25ν_*R*_/ 0.75ν_*R*_ has only a slight effect on the recoupling curves. However, for the 0.25ν_*R*_/ 1.75ν_*R*_ condition, the total Dip signal oscillates more slowly than the other experimental curves, and the depth of the oscillations is smaller. This can be problematic for determination of dipolar coupling values. Changing the ^1^H→^13^C CP condition also affects the CH Dip curves, though to a lesser extent.

**Figure 3:**
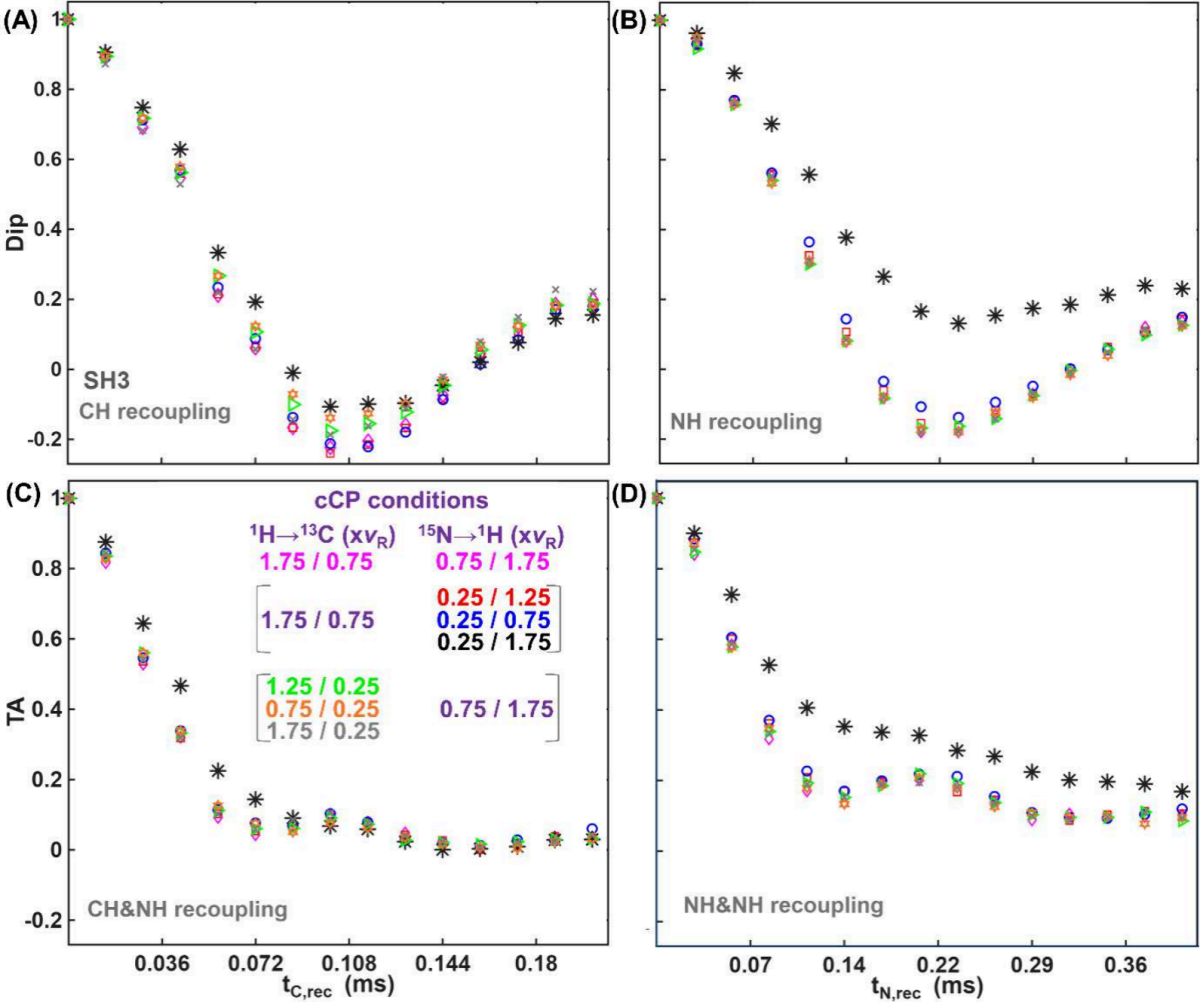
(HCAN)H SH3 dipolar (A-B) and torsion angle (C-D) curves under different CP conditions with continuous shapes (cCP) as shown in the legend.

For sequential CH and NH recoupling (Figure 3C), all TA curves exhibit similar behavior at longer recoupling times, regardless of the CP conditions, and only the curve corresponding to 0.25ν_*R*_/ 1.75ν_*R*_ deviates from others at shorter recoupling times. Since the TA curves shown in Figure 3C consist of the sum of the individual TA curves with different ϕ_*H*_ values, it is not possible to conclude which of the conditions is more favorable for TA determinations, but again emphasizes the impact of the CP.

Similar to Figure 1, the sequential recoupling of the same dipolar interaction (NH) in the (HCAN)H experiment provides insight into the influence of CP conditions on the TA curve (Figure 3D). Only the curve corresponding to 0.25ν_*R*_/ 1.75ν_*R*_ (black stars) lies significantly above the others, while the remaining curves exhibit similar behavior. This suggests that the ^15^N→^1^H CP condition of 0.25ν_*R*_/ 1.75ν_*R*_ is potentially distinct from the other curves, and indeed, a systematic comparison reveals it to be more favorable for torsion angle determinations, despite the drawback of a less oscillatory Dip curve (vide infra). It is worth mentioning that the condition 0.25ν_*R*_/ 1.75ν_*R*_ is not unique; based on simulations, any zero-quantum or double-quantum CP condition that matches twice the MAS rate can have a similar effect.

To experimentally confirm that the CP conditions influence TA curves, two sets of pseudo-4D (H)­CANH spectra were acquired. Figure 4A-B shows TA curves for 44 residues of SH3 acquired with two different CP conditions for the ^15^N→^1^H transfer: 0.75ν_*R*_/1.75ν_*R*_ (A) and of 0.25ν_*R*_/1.75ν_*R*_ (B). The TA curves are colored according to four categories based on the torsion angle values from the 2NUZ crystal structure. Figure 4C–D presents simulated TA curves at different ϕ_*H*_ values, both without and with CP blocks, and a comparison with ideal curves is shown in Figure 4E-F. These data confirm that the ^15^N→^1^H CP condition of 0.25ν_*R*_/1.75ν_*R*_ is qualitatively more favorable for TA determinations compared to 0.75ν_*R*_/1.75ν_*R*_ since the TA curves are more distinctly separated for this condition.

**Figure 4:**
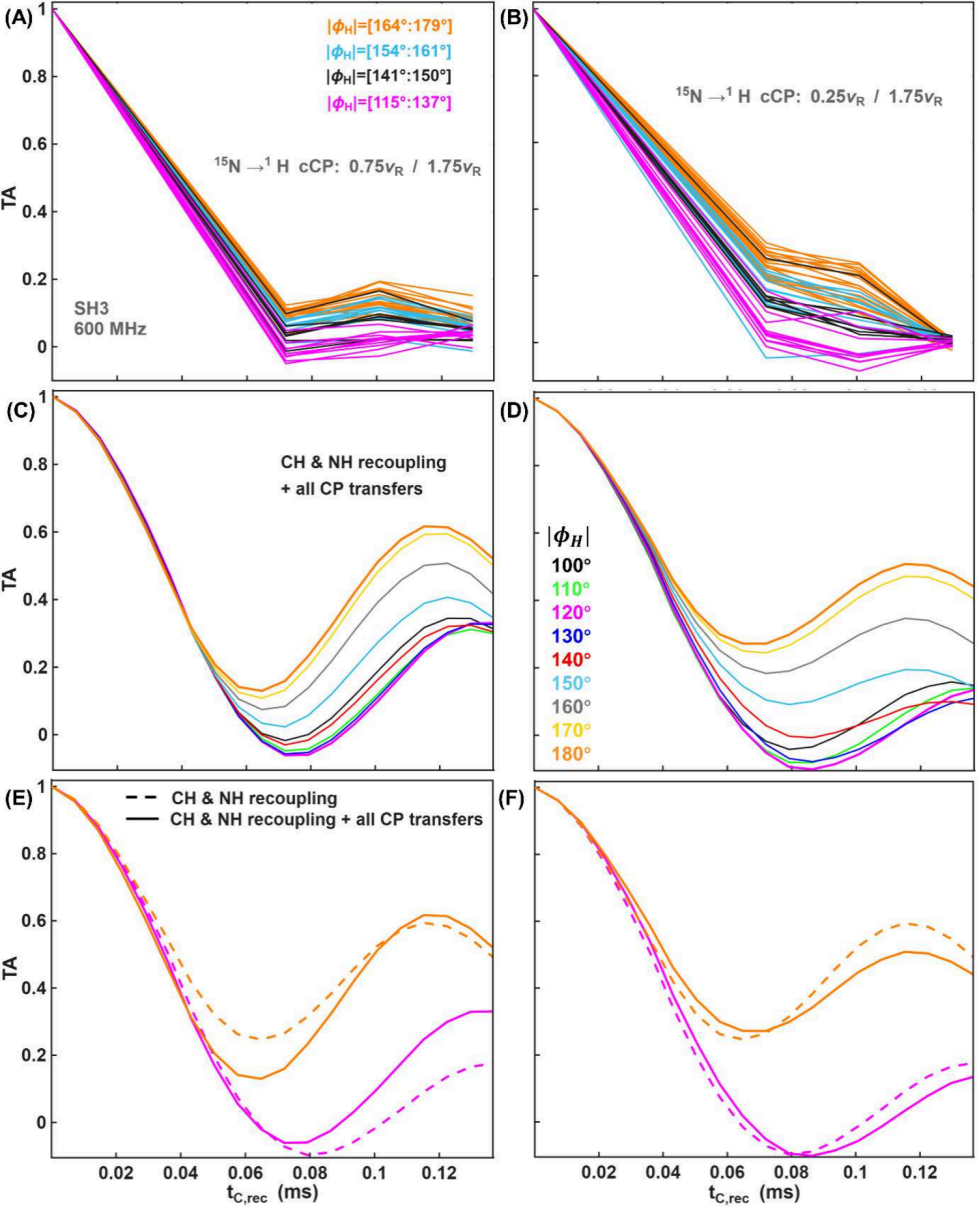
Experimental (A-B) and simulated (C-F) torsion angle curves with CP conditions of 0.75ν_*R*_/1.75ν_*R*_ (A, C, E) and 0.25ν_*R*_/1.75ν_*R*_ (B, D, F) for the ^15^N→^1^H transfer. (A-B) TA curves of 44 amino acid residues obtained from 3D (H)­CANH SH3 spectra, grouped into four categories based on torsion angle values from the 2NUZ crystal structure of SH3 as shown in the legend of panel A. (C–D) Simulated torsion angle curves including CP effects, for selected torsion angle values (ϕ_*H*_). (E-F) Simulated torsion angle curves in the ideal case (dashed lines) and with CP blocks (solid lines), for two torsion angle values.

### Evaluating wCP Conditions

Since quantitative determination of torsion angles using the ^15^N→^1^H CP condition of 0.25ν_*R*_/1.75ν_*R*_ is still a challenge, we investigated wCP conditions as potential alternatives that might simultaneously be optimal for both Dip and TA curves.

Figure 5 compares wCP with cCP. Compared to the continuous ^15^N→^1^H shape, windowed conditions for Dip curves result in a reduced depth of oscillations, as well as a lower effective dipolar scaling factor (Figure 5A and 5B). However, these changes are relatively minor, suggesting that dipolar coupling values can be determined with confidence. Importantly, with windowed CP shapes, the 0° TA curves are lifted closer to the ideal curves (Figure 5D), suggesting improved separation of TA curves and indicating that wCP conditions may be more suitable for the quantitative analysis of TA curves.

**Figure 5:**
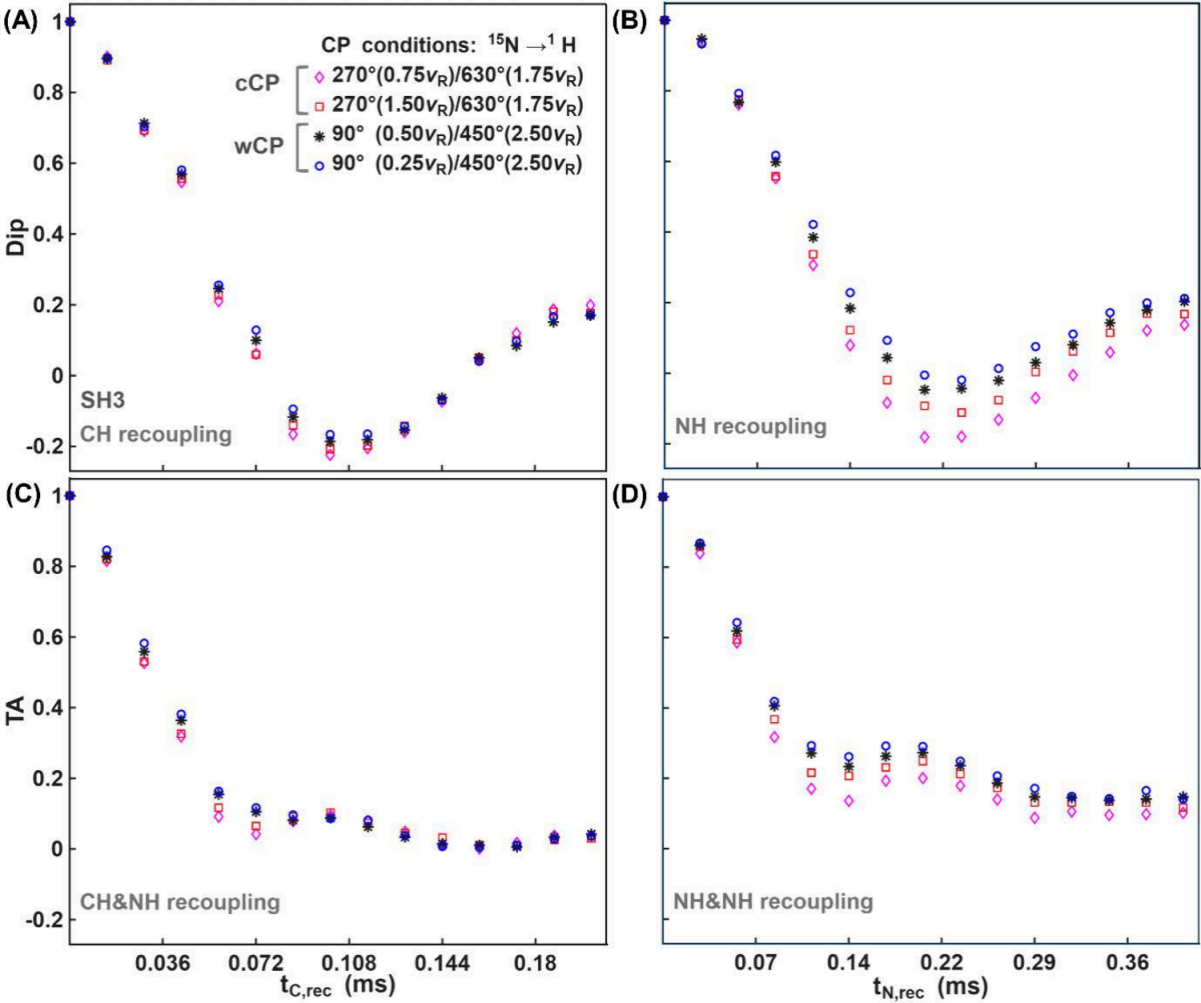
Comparison of wCP and cCP conditions using the SH3 sample and the (HCAN)H transfer pathway. Dipolar (A-B) and torsion angle (C-D) curves for different CP conditions for ^15^N→^1^H transfers. The 1.75ν_*R*_/0.75ν_*R*_ cCP condition was used for ^1^H→^13^C transfer. The legend indicates both the flip angle (in degrees) and the RF field strength (as multiples of the rotor frequency ν_*R*_.

A similar behavior is observed in the torsion angle simulations shown in Figure S7 in the SI. Figure S8A in the SI compares wCP and cCP for the ^1^H→^13^C transfer. The curves are nearly identical. Figure S8B shows signal as a function of the CP transfer time, demonstrating that by 720 μs, a plateau is reached, helping to rationalize the minor changes observed for different CP conditions for the ^1^H →^13^C CP.

An additional important point to mention is that while windowed shapes reduce the dependence of the torsion angle signal on CP blocks, this reduction is not strong enough for their influence to be ignored. Rather than explicit orientation-dependent calculations, the nonidealities – including RF inhomogeneity – can be accounted for through an empirical fitting procedure. This fitting entails two nonstructural parameters (Δα_*rf*, *max*_ and *T*_2, *eff*_, explained in the Fitting procedure section in the SI) and two structural parameters (CH and NH dipolar coupling values), which are then used to obtain torsion angle values through comparison of experimental and simulated TA curves. We previously interpreted Δα_*rf*, *max*_ and *T*_2, *eff*_ as stemming from the effects of RF inhomogeneity alone.^92^

Figure 6 shows experimental TA curves with continuous (A) and windowed (C, E) shapes and the correlation between NMR-derived torsion angles (ϕ_*H*_) and those found in two X-ray crystal structures for each data set (Figure 6B, D and F). Figure 6 shows only one of the possible solutions for |ϕ_*H*_| (that closest to the X-ray values), while two possibilities are presented in Figure S13 in the SI. The RMSD between two different crystal structures of the same crystal form, 2NUZ and 1SHG is 5° degrees, while the NMR-derived angles differ by RMSDs of 10.6° and 9.4° to the two crystal structures. The Δα_*rf*, *max*_, CH and NH dipolar coupling values are summarized in Figures S10 and S12 in the SI. Experimental and simulated Dip and TA curves for 44 amino-acid residues are shown in Figures S15–S23 in the SI.

**Figure 6:**
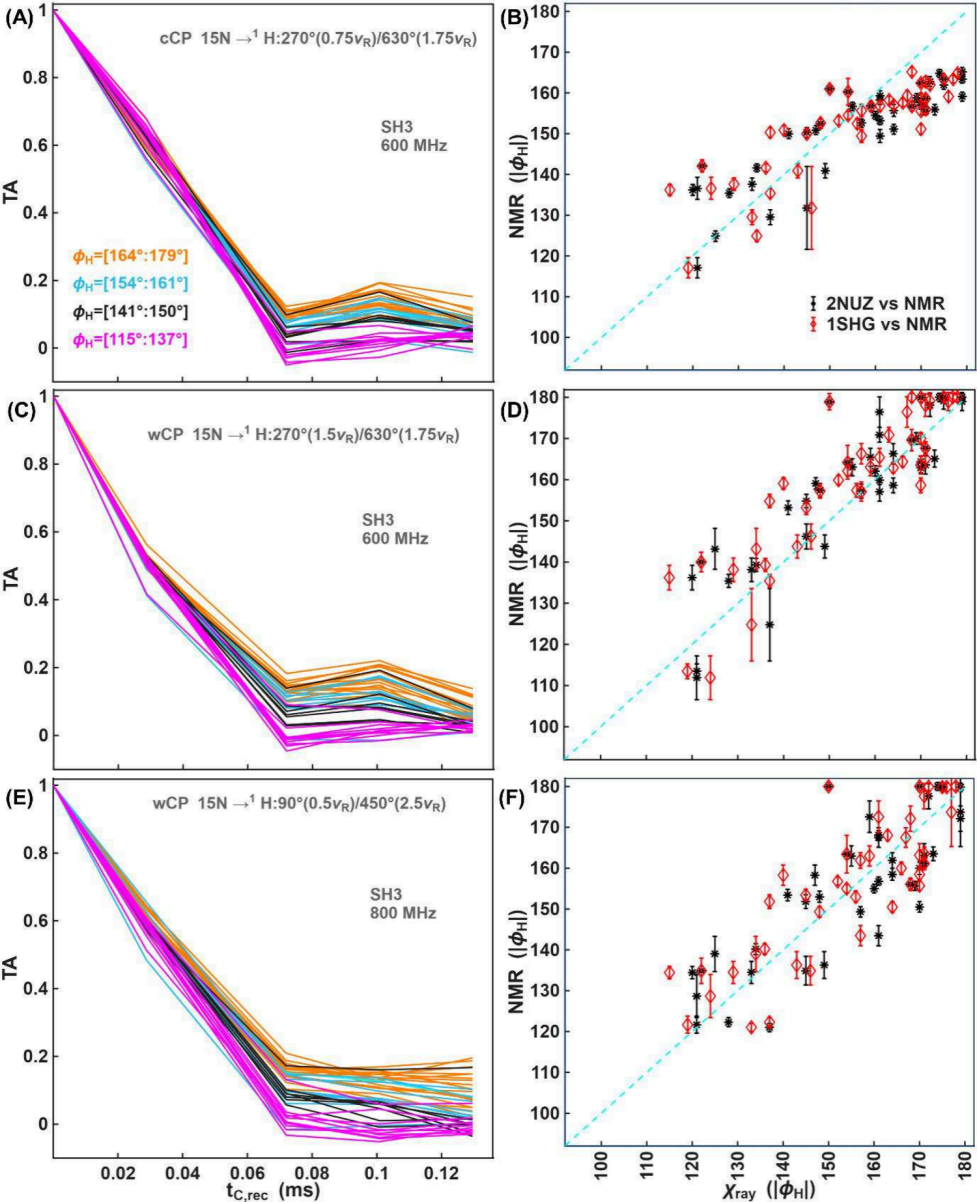
Measurement of torsion angles in microcrystalline SH3 using three different CP conditions for the ^15^N→^1^H transfer: (A, B) cCP and (C-E) wCP conditions as indicated in the legend. Correlation between NMR-derived angles and the X-ray values are shown in (B, D and F) for two X-ray structures: PDB codes 2NUZ (black stars) and 1SHG (red diamonds). For each crystal structure, proton positions were added using ‘add hydrogens’ in ChimeraX. For wCP, the window was half the rotor period and applied on only the nitrogen channel (C–D) or both channels (E-F). For (A) and (C), data were recorded on a 600 MHz spectrometer, while for (E), the data was recorded at 800 MHz. Additional experimental details are provided in the SI. Error bars are shown at 2 times the estimated standard deviation arising from random errors stemming from the spectrum noise. For (F), the last experimental point at 0.1296 ms was excluded.

For |ϕ_*H*_| values smaller than 145°, all three experimental conditions yield similar |ϕ_*H*_| values. The differences arise for torsion angles close to 180°. In that case, the TA experiment with a continuous shape for the ^15^N→^1^H transfer underestimates the |ϕ_*H*_| values (Figure 6B). In contrast, TA experiments with windowed shapes (Figure 6D, F) provide |ϕ_*H*_| values close to 180°, which is more consistent with the two X-ray structures.

We also evaluated the performance of ^15^N–^1^H wCP conditions for determining backbone torsion angles in the membrane protein M2 from Influenza A virus. Measurements were made for the conductance-domain (residues 18–60) of the S31N variant, a drug-resistant strain that is prevalent in seasonal influenza.^112–115^ The experimental TA curves, sorted according to the ϕ_*H*_ values from the NMR structure (PDB code: 2N70),^116^ are shown in Figure 7A,C. Figure 7B,D compares ϕ_*H*_ values obtained with TA measurements and those predicted by TALOS-N.^80^ TA experiments with two different wCP conditions provide similar ϕ_*H*_ values. The Δα_*rf*, *max*_, dipolar coupling and torsion angle values are summarized in the Figures S11 and S14 in the SI. Experimental and simulated Dip and TA curves for 19 amino-acid residues are shown in Figures S24–S29 in the SI.

**Figure 7:**
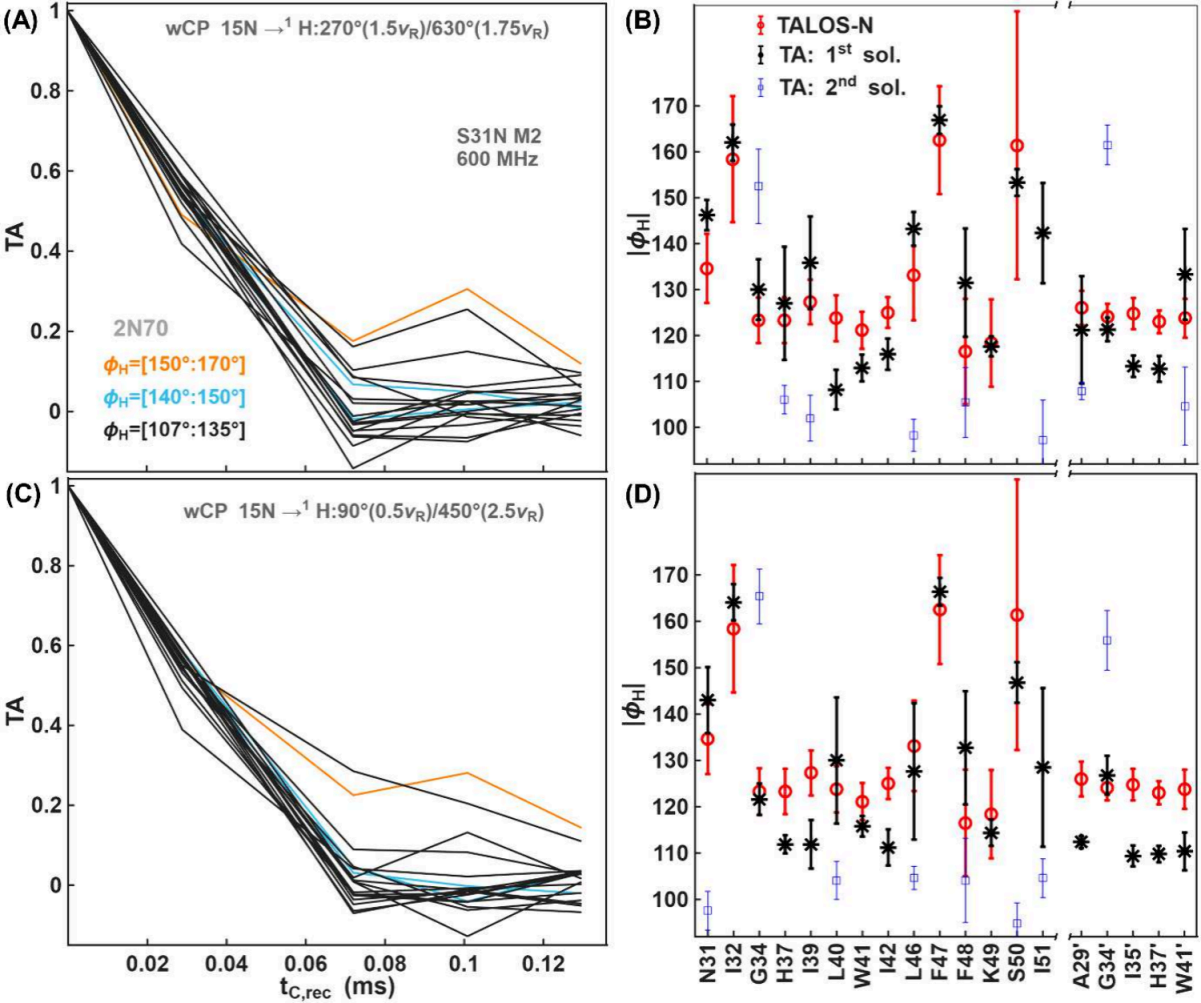
Torsion angle measurements for S31N M2 using two different CP conditions, both with windowed CP for the ^15^N→^1^H transfer. Experimental torsion angle curves (A, C) and the corresponding best fit torsion angle values (B, D) compared with TALOS-N predictions. In (A) the window was on the nitrogen channel, while in (C) the window was applied on both channels. Flip angles and RF-field strengths are indicated in the figure. (A, C) TA curves of 19 amino acid residues (14 in the long and 5 in short chains) obtained from 3D (H)­CANH spectra, grouped into three categories based on |ϕ_*H*_| torsion angle values from the NMR structure (PDB code: 2N70)^116^ as shown in the legend of panel A. For pMODERN data, error bars are shown at 2 times the estimated standard deviation based on spectrum noise. Error bars for TALOS-N are shown at ± the estimated RMS error.

Influenza A M2 is a tetrameric transmembrane membrane protein, that assembles as a dimer of dimers in lipid bilayers,^116,117^ such that two sets of peaks are observed. One of these can be assigned to a longer stretch of residues that includes both the amphipathic helix and transmembrane helix, and a shorter stretch of residues (indicated with a prime symbol) consisting of transmembrane residues. According to the NMR structures (PDB codes: 2N70 ^116^ and 2L0J^118^), a deviation from the ideal helicity is observed at residue F47, with a |ϕ_*H*_| torsion angle of 167.8° and 156°, respectively.

Both TA measurements and TALOS-N confirm a tight and rigid turn at the residue F47 (|ϕ_*H*_|∼162.5°,ϕ∼ –102.5°). Additionally, two other amino-acid residues are found with |ϕ_*H*_| values deviating from ideal α-helical geometry: I32 (∼162°) and S50 (∼153°). While both TA and TALOS-N provide similar values of |ϕ_*H*_|, TA measurements show higher precision, as indicated by the smaller error bars. The torsion angle values based on isotropic chemical shift values (TALOS-N) are much easier to obtain. However, these calculations are still based on database-derived predictions and may therefore exhibit large uncertainties in certain cases, as seen here in particular for S50, F47 and I32. A key advantage of direct torsion angle measurements via tensor correlation is that they can potentially reduce these uncertainties.

An unusual side-chain conformation at residue I32 of the transmembrane helix was recently investigated in our lab^117^ through distance measurements between side-chain carbons and backbone nitrogen spins using the TREDOR sequence.^63^ Both the backbone angle as well as the side chain conformation are likely to be influenced by the neighboring asparagine residue that confers drug resistance.

We showed above that the addition of windows improved the behavior when considering both Dip and TA curves. Using simulations and experiments, we showed that different CP conditions affect Dip and TA curves differently. For further explanation of these differences, we show additional simulations of TA curves for individual orientations. Figure 8 shows such TA orientation plots, which visualize the signal amplitude as a function of both the orientation (*y*-axis) and recoupling time (*x*-axis). Two different cCP conditions and one wCP condition are compared with ideal conditions, for two selected torsion angles.

**Figure 8:**
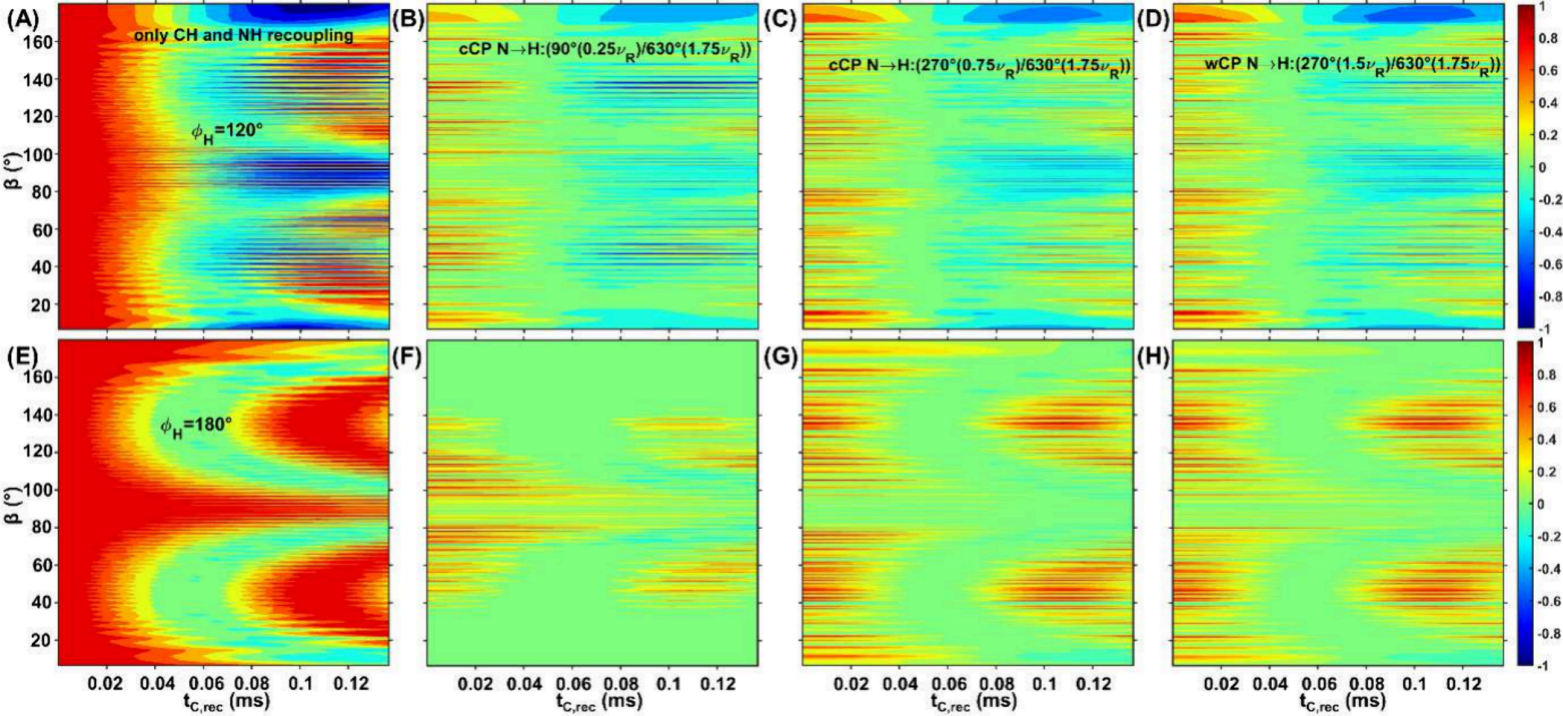
Simulated torsion angle plots are provided for two ϕ_*H*_ values: 120° - (A)-(D) and 180° - (E)-(F) as a function of orientation and mixing time. The orientation-dependent amplitudes are shown as a function of recoupling time and the β angle for the ideal case (A) and (E) and in the presence of three CP blocks for H→C, C→N and N→H transfers (B)-(D) and (F)-(H). The CP condition used for the N–H transfer is indicated in the legend. Note that the signal also depends on the α angle, which is varied together with the β angle and explains the fine structure of the plots.

Under ideal conditions, all orientations – regardless of whether ϕ_*H*_ is 120° (Figure 8A) or 180° (Figure 8E) – have the same amplitude at zero recoupling time (red). As the recoupling time increases, the amplitudes of the orientations are modulated over time. Depending on the β angle and ϕ_*H*_, some amplitudes reach minimum intensities of −1 (blue), drop slightly below zero for (green) or do not dephase at all (red).

In the presence of CP blocks (Figure 8B-D and 8F–H), different orientation regions are detected with different intensities. For example, in the region of β ∈ [160°:180°] with ϕ_*H*_ = 120°, the wCP condition (Figure 8D) detects with higher amplitudes compared to the other two conditions (Figure 8B and 8C). In the region of β ∈ [80°:100°] with ϕ_*H*_ = 180°, the cCP condition of Figure 8F is more efficient than the other two (Figure 8G-H), but the β ∈ [160°:180°] region is hardly detected at all. Since both these regions are particularly dependent on the torsion angle, the better overall performance of the wCP condition can be rationalized since it has more uniform contributions from different orientations that are sensitive to the angle.

While all simulations so far have been performed in the presence of additional distant proton spins, their influence on TA curves has not been fully addressed. Overall, the presence of additional distant dipolar-coupled proton spins has a positive impact on TA curves for both continuous and windowed CP shapes (Figure S30 in the SI). Their influence on TA curves can also be analyzed with orientation plots (Figure 9), where a windowed CP condition is investigated. The torsion angle orientation plots (Figure 9) show that additional dipolar-coupled spins increase the amplitudes of the orientation regions that are sensitive to changes in ϕ_*H*_ – for example, in the regions of β∈[80°:100°] and β∈[160°:180°].

**Figure 9:**
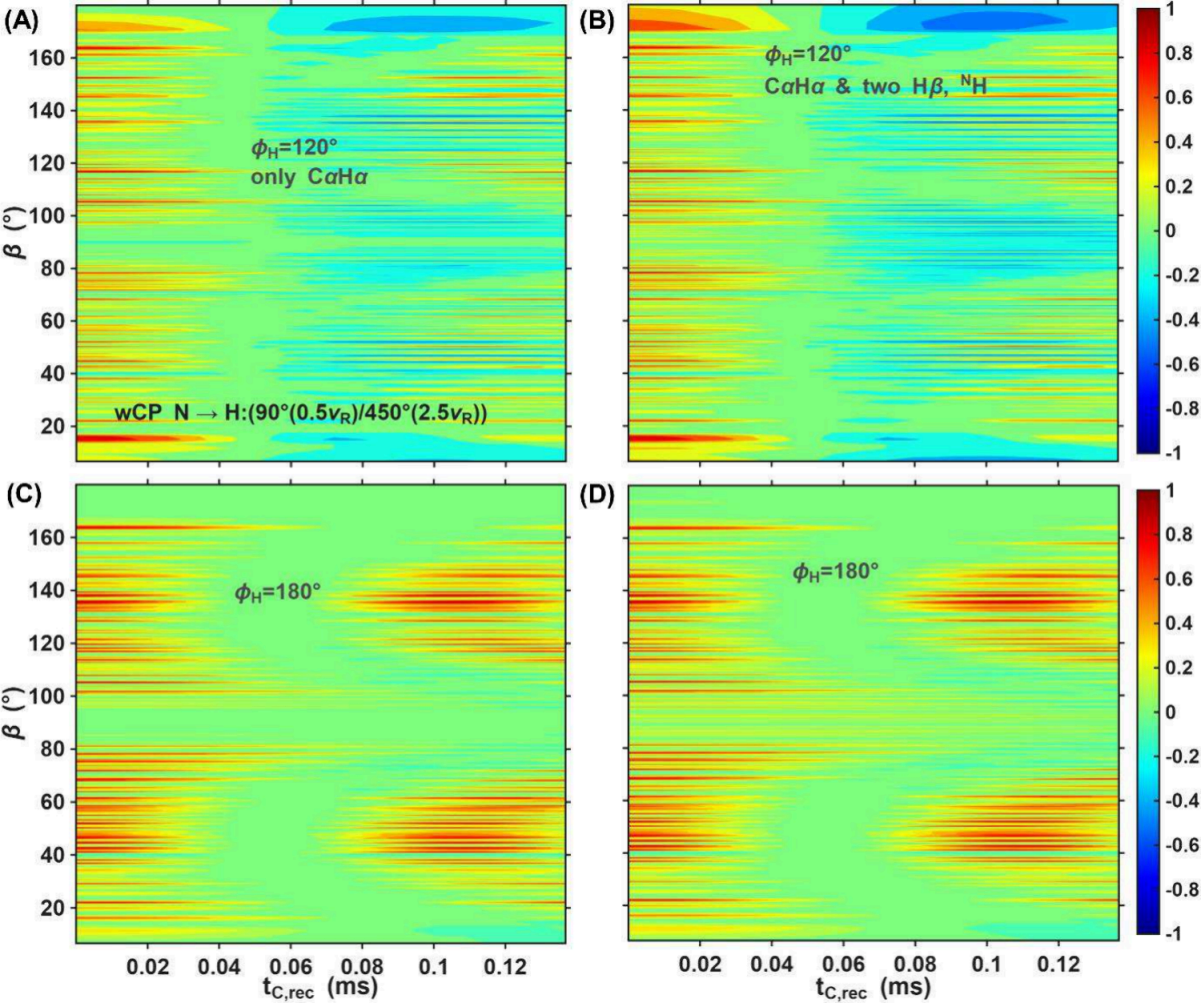
Torsion angle plots are shown as a function of orientation either with only one Hα spin only (A and C) or with three additional dipolar-coupled proton spins (B and D) included in the simulations of the first CP transfer: H→C. The wCP condition is listed in the figure, which shows two ϕ_*H*_ values: 120° (A and B) and 180° (C and D).

The difference in the behavior of Dip curves under various CP conditions can also be analyzed by plotting individual orientations (Figures S31 and S32 in the SI). Interestingly, the orientation regions that are more sensitive to the torsion angle changes (β ∈ [80°:100°]) are at the same time the least sensitive to dipolar coupling changes, as seen in these plots. For this reason, the cCP condition 90°(0.25ν_*R*_)/630°(1.75ν_*R*_) becomes the least efficient for NH dipolar coupling determination in (H)­CANH experiments, since under this condition the region β ∈ [80°:100°] contributes most to the total signal compared to other CP conditions (Figure S32).

Another observation is that the simulated TA curves at longer recoupling times remain distinguishable based on the ϕ_*H*_ value regardless of the CP conditions (Figure 4C and 4D). However, experimental TA curves were observed to converge toward the *x*-axis at longer recoupling times, irrespective of the ϕ_*H*_ value (Figure 4A-B, 6C, E). In fact, the last experimental point of Figure 6E had to be excluded since it significantly deviated from the simulation for some residues (Figures S25–S29 in the SI). The convergence of the experimental TA curves at longer mixing times can be explained by including additional dipolar-coupled spins during the pMODERN sequences (Figure 10) and the proton chemical shift anisotropy (Figure S33 in the SI). Regardless of the CP condition for the N→H transfer, the simulated TA curves at longer recoupling times converge toward the *x*-axis, making the simulations more consistent with the experimental data and further justifying exclusion of later time points.

**Figure 10:**
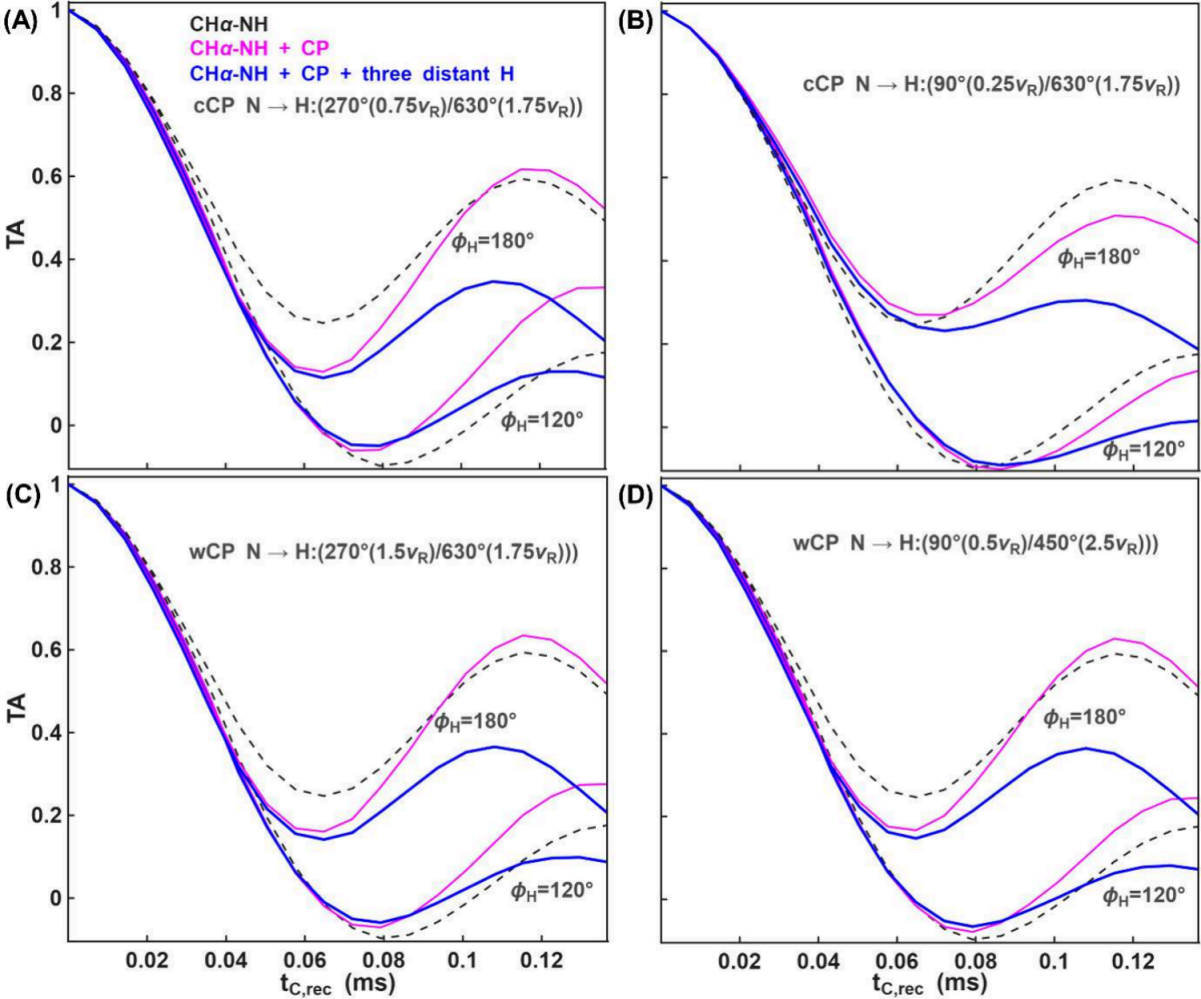
Torsion angle curves with two ϕ_*H*_ values – 120° and 180° – are shown under different N→H CP conditions with continuous (A-B) and half-windowed (C–D) shapes. The solid lines represent torsion angle curves in the presence of CP blocks, with only the directly bonded proton (magenta) or three additional proton spins (blue) during pMODERN sequences. For reference, the dashed lines show the ideal TA curves. For CH, with a dipolar coupling of 20 kHz, additional dipolar-coupled proton spins – two Hβ and one ^N^H spins – from the same amino-acid residues, each with similar dipolar coupling values of 3.1 kHz (corresponding to a 2.14 Å distance), are considered during the pMODERN sequences. For NH, with a dipolar coupling of 10 kHz, two Hα proton spins (from the same and the following amino-acid residues) with dipolar coupling values of 1.35 kHz (corresponding to a 2.14 Å distance) and 0.78 kHz (corresponding to a 2.49 Å distance) and one distant ^N^H spin with dipolar coupling value of 1.1 kHz (corresponding to a 2.23 Å distance) are considered during the pMODERN sequences. The remaining simulation details for CP transfers are the same as those in Figures 8 and 9

Figures S34 shows the impact of cCP on TA curves for different MAS rates. The simulations reveal MAS-dependent changes in the curves, with some angles appearing closer to the ideal curves at low MAS rates, and others closer at high MAS rates. This suggests that orientation selection may still be an issue at lower MAS rates. However, these simulations involved a limited number of spins, and direct comparison of experiments and these simulations for torsion angle determination certainly requires additional evaluation of these effects.

## Conclusions

In summary, this manuscript investigates the influence of CP blocks in 3D (H)­CANH-based torsion angle measurements. This influence manifested as a reduced dependence of the torsion angle curves on the torsion angle values, which in turn decreased the accuracy of torsion angle determination based on the comparison between experimental and ideal TA signals. This effect was explained by the fact that, in the presence of CP blocks, the detected amplitudes at zero recoupling time become dependent upon the orientation of the molecule in the rotor frame. Under these conditions, some orientation regions that were sensitive to torsion angle change contributed less to the overall torsion angle signal.

By using different CP conditions with continuous and windowed shapes, we demonstrated that the latter can reduce the influence of CP blocks when applied during the final transfer from ^15^N→^1^H, making it optimal for both dipolar coupling and torsion angle determination. While windowed CP shapes did not completely eliminate the influence of CP blocks, this influence could be accounted for, which in turn enabled more accurate torsion angle measurements. Comparisons of |ϕ_*H*_| values determined using other methods – X-ray and TALOS-N – showed good agreement, indicating that the proposed conditions enable reliable quantitative analysis of multidimensional NMR data.

Another possible solution could be the use of J-transfer-based blocks instead of CP-transfer-based blocks. However, this approach is only feasible for samples with long relaxation times. For J-transfer-based blocks, additional sample modification, such as α-PET labeling,^119^ can reduce the influence of distant proton spins at longer recoupling times, thereby increasing the accuracy of torsion angle determination.

## Materials and Methods Summary

MODERN and pMODERN simulations were performed using in-house MATLAB scripts with the numerical solution of the equation of motion.^13^ In all simulations, the initial and the final spin operators were H_1,x_ and H_2,x_, respectively. For both experiments and simulations, [80:100](%) ramped CP with a duration of 720 μs was used for forward CP (^1^H→^15^N or ^1^H→^13^C). [100:80](%) ramped CP with a duration of 630 μs was used for the final CP from N to H. For CP with windowed shapes, the window size was 50% of the rotor period. For the ^13^C→^15^N transfer, a SPEPS block was used with a duration of 2.88 ms and RF-field conditions of 0.25ν_*R*_/0.75ν_*R*_. Unless otherwise indicated, the H→C CP was implemented at the 1.75ν_*R*_/ 0.75ν_*R*_ CP condition. RF-field strengths are specified at the midpoint for ramps. Unless noted, data were recorded on an 600 MHz spectrometer with 55.555 kHz MAS. The instrument time required for each point of the pseudo dimension of torsion angle experiments was from 0.5 to 2.33 days (Tables S3–S6).

The spin system for simulation of the H to C CP transfer included four protons (Hα, Hβ1, Hβ2, H^N^), which was necessary to capture certain behaviors of the TA and Dip curves. In all simulations except Figure 10, only directly bonded spins (Hα for Cα and ^N^H for N) were taken into account during the pMODERN sequence. Additional details regarding the simulated spin system are provided in the paragraph related to Figure S6 in the SI.

The SI provides additional details including comparison of MODERN and pMODERN sequences (Figures S1–S2), (H)­NH pulse sequence details and acquisition parameters (Figure S3, Table S1), details regarding FLAN conditions (Figure S5), the spin system geometry (Figure S6), details of the fitting procedure, sample preparation, experimental procedures and parameters (Tables S3–S6), and the Bruker pulse programs.

### Fitting Procedure

To reduce the number of unknown variables in eq 1, three sets of experiments were acquired.^92^ In the first two sets, either CH or NH dipolar couplings are recoupled, while in the third set, both dipolar couplings are sequentially recoupled. From the first two sets, the ν_D, HC_ and ν_D, HN_ values are determined by comparing experimental and simulated signals. Additionally, two fitting parameters are used to better match the experimental and simulated signals, accounting for relaxation effects and inhomogeneity of the applied RF-field in the probe.^110,111^ These four parameters are then used to determine |ϕ_*H*_| by comparing the third set of the experiments with simulations. More details about the fitting procedure are provided in the Fitting procedure section in the SI and in the article of Xue et al.^92^

## Supplementary Material



## Notes

pMODERN pulse sequence and 2D (H)­NH experiments; simulated spin system, FLAN conditions and additional 1D data; fitting procedure; dipolar and torsion angle values in SH3 and S31N M2 samples; dipolar and torsion angle SH3 curves; dipolar and torsion angle S31N M2 curves; additional simulations; experimental methods; Bruker pulse program (PDF)

## References

[1] Ahlawat S., Mote K. R., Lakomek N.-A., Agarwal V. (2022). Solid-State NMR: Methods
for Biological Solids. Chem. Rev..

[2] Yan S., Suiter C. L., Hou G., Zhang H., Polenova T. (2013). Probing Structure
and Dynamics of Protein Assemblies by Magic Angle Spinning NMR Spectroscopy. Acc. Chem. Res..

[3] Quinn C. M., Polenova T. (2017). Structural Biology
of Supramolecular Assemblies by
Magic-Angle Spinning NMR Spectroscopy. Q. Rev.
Biophys..

[4] van
der Wel P. C. A. (2017). Insights into Protein Misfolding and Aggregation Enabled
by Solid-State NMR Spectroscopy. Solid State
Nucl. Magn. Reson..

[5] Mandala V. S., Williams J. K., Hong M. (2018). Structure and Dynamics
of Membrane
Proteins from Solid-State NMR. Annu. Rev. Biophys..

[6] Rienstra C. M., Tucker-Kellogg L., Jaroniec C. P., Hohwy M., Reif B., McMahon M. T., Tidor B., Lozano-Pérez T., Griffin R. G. (2002). De Novo Determination
of Peptide Structure with Solid-State
Magic-Angle Spinning NMR Spectroscopy. Proc.
Natl. Acad. Sci. U. S. A..

[7] Franks W. T., Zhou D. H., Wylie B. J., Money B. G., Graesser D. T., Frericks H. L., Sahota G., Rienstra C. M. (2005). Magic-Angle Spinning
Solid-State NMR Spectroscopy of the Β1 Immunoglobulin Binding
Domain of Protein G (GB1): 15N and 13C Chemical Shift Assignments
and Conformational Analysis. J. Am. Chem. Soc..

[8] Rienstra C.
M., Hohwy M., Mueller L. J., Jaroniec C. P., Reif B., Griffin R. G. (2002). Determination
of Multiple Torsion-Angle Constraints
in U–13C,15N-Labeled Peptides: 3D 1H–15N–13C–1H
Dipolar Chemical Shift NMR Spectroscopy in Rotating Solids. J. Am. Chem. Soc..

[9] Ishii Y., Hirao K., Terao T., Terauchi T., Oba M., Nishiyama K., Kainosho M. (1998). Determination of Peptide *φ* Angles
in Solids by Relayed Anisotropy Correlation
NMR. Solid State Nucl. Magn. Reson..

[10] Reif B., Hohwy M., Jaroniec C. P., Rienstra C. M., Griffin R. G. (2000). NH–NH
Vector Correlation in Peptides by Solid-State NMR. J. Magn. Reson..

[11] Hong M. (1999). Determination
of Multiple φ-Torsion Angles in Proteins by Selective and Extensive
13C Labeling and Two-Dimensional Solid-State NMR. J. Magn. Reson..

[12] Baldus M. (2002). Correlation
Experiments for Assignment and Structure Elucidation of Immobilized
Polypeptides under Magic Angle Spinning. Prog.
Nucl. Magn. Reson. Spectrosc..

[13] Heise H. (2008). Solid-State
NMR Spectroscopy of Amyloid Proteins. ChemBioChem..

[14] Hong M., Zhang Y., Hu F. (2012). Membrane Protein Structure and Dynamics
from NMR Spectroscopy. Annu. Rev. Phys. Chem..

[15] Wang S., Ladizhansky V. (2014). Recent Advances in Magic Angle Spinning Solid State
NMR of Membrane Proteins. Prog. Nucl. Magn.
Reson. Spectrosc..

[16] Thompson L. K. (2002). Solid-State
NMR Studies of the Structure and Mechanisms of Proteins. Curr. Opin. Struct. Biol..

[17] Ladizhansky V., Palani R. S., Mardini M., Griffin R. G. (2024). Dipolar Recoupling
in Rotating Solids. Chem. Rev..

[18] van
der Wel P. C. A. (2021). Dihedral Angle Measurements for Structure Determination
by Biomolecular Solid-State NMR Spectroscopy. Front. Mol. Biosci..

[19] Zhou D. H., Shah G., Cormos M., Mullen C., Sandoz D., Rienstra C. M. (2007). Proton-Detected
Solid-State NMR Spectroscopy of Fully
Protonated Proteins at 40 kHz Magic-Angle Spinning. J. Am. Chem. Soc..

[20] Andreas L. B., Le Marchand T., Jaudzems K., Pintacuda G. (2015). High-Resolution
Proton-Detected NMR of Proteins at Very Fast MAS. J. Magn. Reson..

[21] Andrew E. R., Bradbury A., Eades R. G. (1958). Nuclear Magnetic
Resonance Spectra
from a Crystal Rotated at High Speed. Nature.

[22] Lowe I. J. (1959). Free Induction
Decays of Rotating Solids. Phys. Rev. Lett..

[23] Asami S., Reif B. (2017). Comparative Study of REDOR and CPPI Derived Order Parameters by 1H-Detected
MAS NMR and MD Simulations. J. Phys. Chem. B.

[24] Hong M., Yao X., Jakes K., Huster D. (2002). Investigation of Molecular Motions
by Lee-Goldburg Cross-Polarization NMR Spectroscopy. J. Phys. Chem. B.

[25] Huster D., Xiao L., Hong M. (2001). Solid-State
NMR Investigation of
the Dynamics of the Soluble and Membrane-Bound Colicin Ia Channel-Forming
Domain. Biochemistry.

[26] Franks W. T., Tatman B. P., Trenouth J., Lewandowski J. R. (2021). Dipolar
Order Parameters in Large Systems With Fast Spinning. Front. Mol. Biosci..

[27] Xue K., Mühlbauer M., Mamone S., Sarkar R., Reif B. (2019). Accurate Determination
of 1H-15N Dipolar Couplings Using Inaccurate Settings of the Magic
Angle in Solid-State NMR Spectroscopy. Angew.
Chem., Int. Ed..

[28] Xue K., Mamone S., Koch B., Sarkar R., Reif B. (2019). Determination
of Methyl Order Parameters Using Solid State NMR under off Magic Angle
Spinning. J. Biomol. NMR.

[29] Nishiyama Y., Malon M., Potrzebowski M. J., Paluch P., Amoureux J. P. (2016). Accurate
NMR Determination of C–H or N–H Distances for Unlabeled
Molecules. Solid State Nucl. Magn. Reson..

[30] Hou G., Lu X., Vega A. J., Polenova T. (2014). Accurate Measurement of Heteronuclear
Dipolar Couplings by Phase-Alternating R-Symmetry (PARS) Sequences
in Magic Angle Spinning NMR Spectroscopy. J.
Chem. Phys..

[31] Schanda P., Meier B. H., Ernst M. (2011). Accurate Measurement of One-Bond
H–X Heteronuclear Dipolar Couplings in MAS Solid-State NMR. J. Magn. Reson..

[32] Carravetta M., Edén M., Zhao X., Brinkmann A., Levitt M. H. (2000). Symmetry Principles
for the Design of Radiofrequency
Pulse Sequences in the Nuclear Magnetic Resonance of Rotating Solids. Chem. Phys. Lett..

[33] Brinkmann A., Levitt M. H. (2001). Symmetry Principles
in the Nuclear Magnetic Resonance
of Spinning Solids: Heteronuclear Recoupling by Generalized Hartmann–Hahn
Sequences. J. Chem. Phys..

[34] Liang L., Ji Y., Chen K., Gao P., Zhao Z., Hou G. (2022). Solid-State
NMR Dipolar and Chemical Shift Anisotropy Recoupling Techniques for
Structural and Dynamical Studies in Biological Systems. Chem. Rev..

[35] Hohwy M., Jaroniec C. P., Reif B., Rienstra C. M., Griffin R. G. (2000). Local Structure
and Relaxation in Solid-State NMR: Accurate Measurement of Amide N–H
Bond Lengths and H–N–H Bond Angles. J. Am. Chem. Soc..

[36] Huster D. (2005). Investigations
of the Structure and Dynamics of Membrane-Associated Peptides by Magic
Angle Spinning NMR. Prog. Nucl. Magn. Reson.
Spectrosc..

[37] Seidel K., Etzkorn M., Heise H., Becker S., Baldus M. (2005). High-Resolution
Solid-State NMR Studies on Uniformly [13C,15N]-Labeled Ubiquitin. ChemBioChem..

[38] Chevelkov V., Fink U., Reif B. (2009). Accurate Determination
of Order Parameters
from 1H,15N Dipolar Couplings in MAS Solid-State NMR Experiments. J. Am. Chem. Soc..

[39] Lu X., Zhang H., Lu M., Vega A. J., Hou G., Polenova T. (2016). Improving Dipolar Recoupling
for Site-Specific Structural
and Dynamics Studies in Biosolids NMR: Windowed RN-Symmetry Sequences. Phys. Chem. Chem. Phys..

[40] Zhao X., Edén M., Levitt M. H. (2001). Recoupling of Heteronuclear Dipolar
Interactions in Solid-State NMR Using Symmetry-Based Pulse Sequences. Chem. Phys. Lett..

[41] Edén M. (2003). Enhanced Symmetry-Based
Dipolar Recoupling in Solid-State NMR. Chem.
Phys. Lett..

[42] Dvinskikh S. V., Zimmermann H., Maliniak A., Sandström D. (2005). Heteronuclear
Dipolar Recoupling in Solid-State Nuclear Magnetic Resonance by Amplitude-,
Phase-, and Frequency-Modulated Lee–Goldburg Cross-Polarization. J. Chem. Phys..

[43] Nimerovsky E., Soutar C. P. (2020). A Modification of
γ-Encoded RN Symmetry Pulses
for Increasing the Scaling Factor and More Accurate Measurements of
the Strong Heteronuclear Dipolar Couplings. J. Magn. Reson..

[44] Paluch P., Pawlak T., Ławniczak K., Trébosc J., Lafon O., Amoureux J.-P., Potrzebowski M. J. (2018). Simple
and Robust Study of Backbone Dynamics of Crystalline Proteins Employing
1H–15N Dipolar Coupling Dispersion. J.
Phys. Chem. B.

[45] Good D. B., Wang S., Ward M. E., Struppe J., Brown L. S., Lewandowski J. R., Ladizhansky V. (2014). Conformational Dynamics of a Seven
Transmembrane Helical Protein Anabaena Sensory Rhodopsin Probed by
Solid-State NMR. J. Am. Chem. Soc..

[46] Zhao X., Sudmeier J. L., Bachovchin W. W., Levitt M. H. (2001). Measurement of NH
Bond Lengths by Fast Magic-Angle Spinning Solid-State NMR Spectroscopy:
A New Method for the Quantification of Hydrogen Bonds. J. Am. Chem. Soc..

[47] Jain M. G., Rajalakshmi G., Madhu P. K., Agarwal V., Mote K. R. (2020). Overcoming
Prohibitively Large Radiofrequency Demands for the Measurement of
Internuclear Distances with Solid-State NMR under Fast Magic-Angle
Spinning. J. Phys. Chem. B.

[48] Jain M. G., Mote K. R., Hellwagner J., Rajalakshmi G., Ernst M., Madhu P. K., Agarwal V. (2019). Measuring
Strong One-Bond
Dipolar Couplings Using REDOR in Magic-Angle Spinning Solid-State
NMR. J. Chem. Phys..

[49] Mehring, M. Principles of High Resolution NMR in Solids; Springer: Berlin, 1983; 10.1007/978-3-642-68756-3.

[50] Wylie B. J., Rienstra C. M. (2008). Multidimensional
Solid State NMR of Anisotropic Interactions
in Peptides and Proteins. J. Chem. Phys..

[51] Sehrawat N., Nehra E., Kumar Rohilla K., Kobayashi T., Nishiyama Y., Kumar Pandey M. (2023). Determination
of the Relative Orientation
between 15N-1H Dipolar Coupling and 1H Chemical Shift Anisotropy Tensors
under Fast MAS Solid-State NMR. J. Magn. Reson..

[52] Mukhopadhyay D., Gupta C., Theint T., Jaroniec C. P. (2018). Peptide Bond Conformation
in Peptides and Proteins Probed by Dipolar Coupling-Chemical Shift
Tensor Correlation Solid-State NMR. J. Magn.
Reson..

[53] Czinki E., Császár A. G., Magyarfalvi G., Schreiner P. R., Allen W. D. (2007). Secondary Structures of Peptides
and Proteins via NMR Chemical-Shielding Anisotropy (CSA) Parameters. J. Am. Chem. Soc..

[54] Chan J. C. C., Tycko R. (2003). Solid-State NMR Spectroscopy
Method for Determination
of the Backbone Torsion Angle ψ in Peptides with Isolated Uniformly
Labeled Residues. J. Am. Chem. Soc..

[55] Ishii Y., Terao T., Kainosho M. (1996). Relayed Anisotropy
Correlation NMR:
Determination of Dihedral Angles in Solids. Chem. Phys. Lett..

[56] Hou G., Paramasivam S., Byeon I.-J. L., Gronenborn A. M., Polenova T. (2010). Determination of Relative Tensor Orientations by γ-Encoded
Chemical Shift Anisotropy/Heteronuclear Dipolar Coupling 3D NMR Spectroscopy
in Biological Solids. Phys. Chem. Chem. Phys..

[57] Macholl S., Sack I., Limbach H.-H., Pauli J., Kelly M., Buntkowsky G. (2000). Solid-State
NMR Study of the SH3 Domain of α-Spectrin:
Application of 13C–15N TEDOR and REDOR. Magn. Reson. Chem..

[58] Wi S., Spano J. (2011). Site-Specific *ϕ*- and *ψ*-Torsion Angle Determination
in a Uniformly/Extensively 13C- and
15N-Labeled Peptide. J. Magn. Reson..

[59] Sinha N., Hong M. (2003). X–1H Rotational-Echo Double-Resonance NMR for Torsion Angle
Determination of Peptides. Chem. Phys. Lett..

[60] Sack I., Balazs Y. S., Rahimipour S., Vega S. (2000). Solid-State NMR Determination
of Peptide Torsion Angles: Applications of 2H-Dephased REDOR. J. Am. Chem. Soc..

[61] Wi S., Sinha N., Hong M. (2004). Long-Range
1H–19F Distance
Measurement in Peptides by Solid-State NMR. J. Am. Chem. Soc..

[62] Luca S., Heise H., Baldus M. (2003). High-Resolution
Solid-State NMR Applied
to Polypeptides and Membrane Proteins. Acc.
Chem. Res..

[63] Zhang X. C., Forster M. C., Nimerovsky E., Movellan K. T., Andreas L. B. (2021). Transferred-Rotational-Echo
Double Resonance. J. Phys. Chem. A.

[64] Castellani F., van Rossum B.-J., Diehl A., Rehbein K., Oschkinat H. (2003). Determination
of Solid-State NMR Structures of Proteins by Means of Three-Dimensional
15N–13C–13C Dipolar Correlation Spectroscopy and Chemical
Shift Analysis. Biochemistry.

[65] Tycko R. (2007). Symmetry-Based
Constant-Time Homonuclear Dipolar Recoupling in Solid State NMR. J. Chem. Phys..

[66] Grage S. L., Watts J. A., Watts A. (2004). 2H­{19F} REDOR
for Distance Measurements
in Biological Solids Using a Double Resonance Spectrometer. J. Magn. Reson..

[67] Andreas L. B., Mehta A. K., Mehta M. A. (2007). Determination
of Global Structure
from Distance and Orientation Constraints in Biological Solids Using
Solid-State NMR Spectroscopy. J. Am. Chem. Soc..

[68] O’Connor R. D., Schaefer J. (2002). Relative CSA–Dipolar
Orientation from REDOR
Sidebands. J. Magn. Reson..

[69] Hong M., Gross J. D., Hu W., Griffin R. G. (1998). Determination of
the Peptide Torsion Angle φ by15N Chemical Shift and13Cα-1HαDipolar
Tensor Correlation in Solid-State MAS NMR. J.
Magn. Reson..

[70] Blanco F. J., Tycko R. (2001). Determination of Polypeptide Backbone Dihedral Angles in Solid State
NMR by Double Quantum 13C Chemical Shift Anisotropy Measurements. J. Magn. Reson..

[71] Feng X., Verdegem P. J. E., Lee Y. K., Sandström D., Edén M., Bovee-Geurts P., de Grip W. J., Lugtenburg J., de Groot H. J. M., Levitt M. H. (1997). Direct
Determination of a Molecular
Torsional Angle in the Membrane Protein Rhodopsin by Solid-State NMR. J. Am. Chem. Soc..

[72] Feng X., Edén M., Brinkmann A., Luthman H., Eriksson L., Gräslund A., Antzutkin O. N., Levitt M. H. (1997). Direct Determination
of a Peptide Torsional Angle ψ by Double-Quantum Solid-State
NMR. J. Am. Chem. Soc..

[73] Costa P. R., Gross J. D., Hong M., Griffin R. G. (1997). Solid-State NMR
Measurement of *Ψ* in Peptides: A NCCN 2Q-Heteronuclear
Local Field Experiment. Chem. Phys. Lett..

[74] Edwards R., Madine J., Fielding L., Middleton D. A. (2010). Measurement
of Multiple Torsional Angles from One-Dimensional Solid-State NMR
Spectra: Application to the Conformational Analysis of a Ligand in
Its Biological Receptor Site. Phys. Chem. Chem.
Phys..

[75] Schmidt-Rohr K. (1996). A Double-Quantum
Solid-State NMR Technique for Determining Torsion Angles in Polymers. Macromolecules.

[76] Ladizhansky V., Jaroniec C. P., Diehl A., Oschkinat H., Griffin R. G. (2003). Measurement of Multiple ψ Torsion
Angles in Uniformly
13C,15N-Labeled α-Spectrin SH3 Domain Using 3D 15N–13C–13C–15N
MAS Dipolar-Chemical Shift Correlation Spectroscopy. J. Am. Chem. Soc..

[77] Feng X., Verdegem P. J. E., Edén M., Sandström D., Lee Y. K., Bovee-Geurts P. H. M., de Grip W. J., Lugtenburg J., de Groot H. J. M., Levitt M. H. (2000). Determination
of a Molecular Torsional
Angle in the Metarhodopsin-I Photointermediate of Rhodopsin by Double-Quantum
Solid-State NMR. J. Biomol. NMR.

[78] van
Beek J. D., Meier B. H. (2006). A DOQSY Approach for the Elucidation
of Torsion Angle Distributions in Biopolymers: Application to Silk. J. Magn. Reson..

[79] Mehta M. A., Eddy M. T., McNeill S. A., Mills F. D., Long J. R. (2008). Determination
of Peptide Backbone Torsion Angles Using Double-Quantum Dipolar Recoupling
Solid-State NMR Spectroscopy. J. Am. Chem. Soc..

[80] Shen Y., Bax A. (2013). Protein Backbone and
Sidechain Torsion Angles Predicted from NMR
Chemical Shifts Using Artificial Neural Networks. J. Biomol. NMR.

[81] Hong M., Gross J. D., Griffin R. G. (1997). Site-Resolved Determination
of Peptide
Torsion Angle φ from the Relative Orientations of Backbone N–H
and C–H Bonds by Solid-State NMR. J.
Phys. Chem. B.

[82] Huster D., Yamaguchi S., Hong M. (2000). Efficient β-Sheet Identification
in Proteins by Solid-State NMR Spectroscopy. J. Am. Chem. Soc..

[83] Metz G., Wu X. L., Smith S. O. (1994). Ramped-Amplitude
Cross Polarization
in Magic-Angle-Spinning NMR. J. Magn. Reson.
A.

[84] Gullion T., Schaefer J. (1989). Rotational-Echo Double-Resonance NMR. J. Magn. Reson. 1969.

[85] Rhim W., Elleman D. D., Vaughan R. W. (1973). Enhanced
Resolution for Solid State
NMR. J. Chem. Phys..

[86] Bielecki A., Kolbert A. C., Levitt M. H. (1989). Frequency-Switched
Pulse Sequences:
Homonuclear Decoupling and Dilute Spin NMR in Solids. Chem. Phys. Lett..

[87] Cady S. D., Hong M. (2008). Amantadine-Induced
Conformational and Dynamical Changes of the Influenza
M2 Transmembrane Proton Channel. Proc. Natl.
Acad. Sci. U. S. A..

[88] Zhang Y., Doherty T., Li J., Lu W., Barinka C., Lubkowski J., Hong M. (2010). Resonance Assignment
and Three-Dimensional
Structure Determination of a Human α-Defensin, HNP-1, by Solid-State
NMR. J. Mol. Biol..

[89] Takegoshi K., Imaizumi T., Terao T. (2000). One- and Two-Dimensional
13C–1H/15N–1H
Dipolar Correlation Experiments under Fast Magic-Angle Spinning for
Determining the Peptide Dihedral Angle *φ*. Solid State Nucl. Magn. Reson..

[90] Takegoshi K., Terao T. (1999). 13C-1H Dipolar Recoupling
under Very Fast Magic-Angle Spinning Using
Virtual Pulses. Solid State Nucl. Magn. Reson..

[91] Baldus M., Petkova A. T., Herzfeld J., Griffin R. G. (1998). Cross Polarization
in the Tilted Frame: Assignment and Spectral Simplification in Heteronuclear
Spin Systems. Mol. Phys..

[92] Xue K., Nimerovsky E., Tekwani Movellan K. A., Becker S., Andreas L. B. (2022). Backbone
Torsion Angle Determination Using Proton Detected Magic-Angle Spinning
Nuclear Magnetic Resonance. J. Phys. Chem. Lett..

[93] Kurz R., Cobo M. F., Ribeiro de Azevedo E., Sommer M., Wicklein A., Thelakkat M., Hempel G., Saalwächter K. (2013). Avoiding Bias
Effects in NMR Experiments for Heteronuclear Dipole–Dipole
Coupling Determinations: Principles and Application to Organic Semiconductor
Materials. ChemPhysChem.

[94] Taware P. P., Jain M. G., Raran-Kurussi S., Agarwal V., Madhu P. K., Mote K. R. (2023). Measuring Dipolar
Order Parameters in Nondeuterated
Proteins Using Solid-State NMR at the Magic-Angle-Spinning Frequency
of 100 kHz. J. Phys. Chem. Lett..

[95] Munowitz M. G., Griffin R. G., Bodenhausen G., Huang T. H. (1981). Two-Dimensional
Rotational Spin-Echo Nuclear Magnetic Resonance in Solids: Correlation
of Chemical Shift and Dipolar Interactions. J. Am. Chem. Soc..

[96] Linser R., Bardiaux B., Higman V., Fink U., Reif B. (2011). Structure
Calculation from Unambiguous Long-Range Amide and Methyl 1H–1H
Distance Restraints for a Microcrystalline Protein with MAS Solid-State
NMR Spectroscopy. J. Am. Chem. Soc..

[97] Haasnoot C.
A. G., van de
Ven F. J. M., Hilbers C. W. (1984). COCONOSY. Combination
of 2D Correlated and 2D Nuclear Overhauser Enhancement Spectroscopy
in a Single Experiment. J. Magn. Reson..

[98] Mehring, M. Principles of High Resolution NMR in Solids, 2nd ed.; Springer-Verlag: Berlin, 1983; 10.1007/978-3-642-68756-3.

[99] Reif B., Hohwy M., Jaroniec C. P., Rienstra C. M., Griffin R. G. (2000). NH–NH
Vector Correlation in Peptides by Solid-State NMR. J. Magn. Reson..

[100] Burum D. P., Ernst R. R. (1980). Net Polarization
Transfer via a *J*-Ordered State for Signal Enhancement
of Low-Sensitivity
Nuclei. J. Magn. Reson..

[101] Penzel S., Smith A. A., Agarwal V., Hunkeler A., Org M.-L., Samoson A., Böckmann A., Ernst M., Meier B. H. (2015). Protein Resonance Assignment at MAS
Frequencies Approaching 100 kHz: A Quantitative Comparison of J-Coupling
and Dipolar-Coupling-Based Transfer Methods. J. Biomol. NMR.

[102] Knight M. J., Webber A. L., Pell A. J., Guerry P., Barbet-Massin E., Bertini I., Felli I. C., Gonnelli L., Pierattelli R., Emsley L., Lesage A., Herrmann T., Pintacuda G. (2011). Fast Resonance Assignment and Fold Determination of
Human Superoxide Dismutase by High-Resolution Proton-Detected Solid-State
MAS NMR Spectroscopy. Angew. Chem., Int. Ed..

[103] Nimerovsky E., Becker S., Andreas L. B. (2023). Windowed Cross Polarization
at 55 kHz Magic-Angle Spinning. J. Magn. Reson..

[104] Nimerovsky E., Varkey A. C., Kim M., Becker S., Andreas L. B. (2023). Simplified Preservation of Equivalent
Pathways Spectroscopy. JACS Au.

[105] Thakur R. S., Kurur N. D., Madhu P. K. (2006). Swept-Frequency
Two-Pulse Phase Modulation for Heteronuclear Dipolar Decoupling in
Solid-State NMR. Chem. Phys. Lett..

[106] Shaka A. J., Keeler J., Frenkiel T., Freeman R. (1983). An Improved
Sequence for Broadband Decoupling: WALTZ-16. J. Magn. Reson..

[107] Li Y., Wylie B. J., Rienstra C. M. (2006). Selective Refocusing
Pulses in Magic-Angle
Spinning NMR: Characterization and Applications to Multi-Dimensional
Protein Spectroscopy. J. Magn. Reson..

[108] Marks D., Vega S. (1996). A Theory for Cross-Polarization NMR
of Nonspinning and Spinning Samples. J. Magn.
Reson. A.

[109] Rovnyak D. (2008). Tutorial on Analytic Theory for Cross-Polarization
in Solid State NMR. Concepts Magn. Reson. Part
A.

[110] Paulson E. K., Martin R. W., Zilm K. W. (2004). Cross Polarization,
Radio Frequency Field Homogeneity, and Circuit Balancing in High Field
Solid State NMR Probes. J. Magn. Reson..

[111] Engelke F. (2002). Electromagnetic
Wave Compression and Radio Frequency
Homogeneity in NMR Solenoidal Coils: Computational Approach. Concepts Magn. Reson..

[112] Sugrue R. J., Hay A. J. (1991). Structural Characteristics
of the
M2 Protein of Influenza a Viruses: Evidence That It Forms a Tetrameric
Channe. Virology.

[113] Andreas L. B., Eddy M. T., Chou J. J., Griffin R. G. (2012). Magic-Angle-Spinning
NMR of the Drug Resistant S31N M2 Proton Transporter from Influenza
A. J. Am. Chem. Soc..

[114] Wang C., Lamb R. A., Pinto L. H. (1995). Activation
of the
M2 Ion Channel of Influenza Virus: A Role for the Transmembrane Domain
Histidine Residue. Biophys. J..

[115] Yi M., Cross T. A., Zhou H.-X. (2008). A Secondary Gate As a Mechanism for
Inhibition of the M2 Proton Channel by Amantadine. J. Phys. Chem. B.

[116] Andreas L. B., Reese M., Eddy M. T., Gelev V., Ni Q. Z., Miller E. A., Emsley L., Pintacuda G., Chou J. J., Griffin R. G. (2015). Structure and Mechanism
of the Influenza
A M218–60 Dimer of Dimers. J. Am. Chem.
Soc..

[117] Stampolaki M., Varkey A. C., Nimerovsky E., Leonov A., Becker S., Andreas L. B. (2025). Seeing Double: The
Persistent Dimer-of-Dimers Structure of Drug Resistant Influenza A
M2. Chem. – Eur. J..

[118] Sharma M., Yi M., Dong H., Qin H., Peterson E., Busath D. D., Zhou H.-X., Cross T. A. (2010). Insight
into the Mechanism of the Influenza A Proton Channel from a Structure
in a Lipid Bilayer. Science.

[119] Movellan K. T., Najbauer E. E., Pratihar S., Salvi M., Giller K., Becker S., Andreas L. B. (2019). Alpha Protons
as
NMR Probes in Deuterated Proteins. J. Biomol.
Nmr.

