# Supplementary material for: Orientation Selection in Proton-Detected Magic-Angle Spinning Torsion Angle Experiments
Source: J Phys Chem A. 2026 Mar 2;130(10):2211–24. doi: 10.1021/acs.jpca.5c07723 (PMC12990111; doi:10.1021/acs.jpca.5c07723)
Supplement: Supplementary file 1 [file jp5c07723_si_001.pdf]

Orientation-selection in proton-detected magic-angle spinning torsion angle experiments

Authors: *Evgeny Nimerovsky*, Marianna Stampolaki, Venus Singh Mithu, Stefan Becker & Loren B. Andreas**

Affiliations:

Department of NMR-based Structural Biology, Max Planck Institute for Multidisciplinary Sciences, Am Faßberg 11, Göttingen 37077, Germany

*Corresponding authors: land@mpinat.mpg.de ORCID: 0000-0003-3216-9065 and evni@mpinat.mpg.de ORCID: 0000-0003-3002-0718.

Contents

***The pMODERN pulse sequence and 2D (H)NH experiments* ... 2**

***The simulated spin system, FLAN conditions and additional 1D data* ... 9**

***Fitting procedure* ... 14**

***Dipolar and torsion angle values in SH3 and S31N M2 samples* ... 18**

***Dipolar and torsion angle SH3 curves* ... 23**

***Dipolar and torsion angle S31N M2 curves* ... 50**

***Additional simulations* ... 59**

***Experimental methods* ... 64**

Simulations ... 64

Sample Preparation ... 64

Solid state NMR spectroscopy ... 64

***BRUKER PULSE PROGRAM* ... 68**

***References* ... 76**

The pMODERN pulse sequence and 2D (H)NH experiments

The pMODERN basis element (shown in Figure S1C) consists of four pulses with identical 99° flip angles. The phases of the four pulses are 124°, 0°, 123°, 180°. The duration (t_p) of each pulse is $0.2T_R$ ($T_R = 1/\nu_R$, where ν_R is the MAS rate in kHz) and accordingly, the RF-field strength is $2.75\nu_R$. The MODERN basis element[1] is depicted in Figure S1A.

Figures S1B and S1D compare simulated dipolar profiles as functions of recoupling time (x-axis) and RF-field strength (ν_{rf}, y-axis, in MAS units) for a 2 kHz dipolar coupling. Figure S2 shows simulated dipolar profiles with a 10 kHz dipolar coupling compared with experimental NH dipolar recoupling using the (HCAN)H sequence and a microcrystalline SH3 sample. Please note that the

experimental 1D (HCAN)H signal is a bulk signal, representing the sum of dipolar recoupling curves arising from different amino acid residues.

Overall, pMODERN exhibits reduced sensitivity to RF-field missettings compared to MODERN. While the experimental signal dephased to only -3% with the MODERN sequence (Figure S2B), it reached -18% with pMODERN (Figure S2D). At the same time, it preserves the same behavior as the MODERN sequence – the experimental imperfections mainly affect the depth of dipolar oscillations.

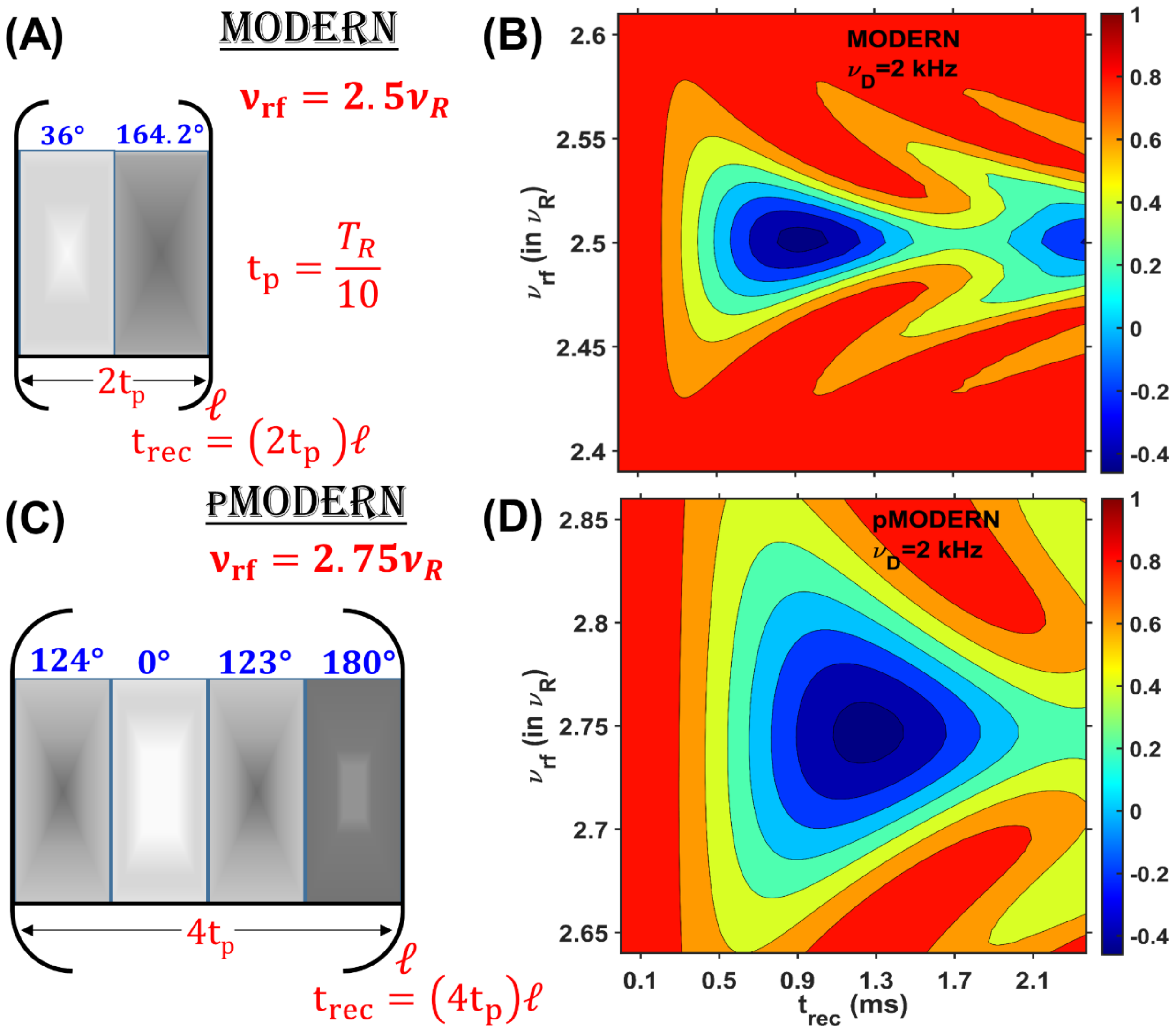

Figure S1 Comparison of MODERN and pMODERN in simulation. (A) MODERN and (C) The pMODERN basis elements. (B, D) Simulated dipolar recoupling signals as a function of recoupling time (x-axis) and RF-field strength (y-axis, in MAS units) using MODERN (B) and pMODERN (D) sequences. In each case, a two-spin system with a 2 kHz dipolar coupling value and a 55.555 kHz MAS was simulated.

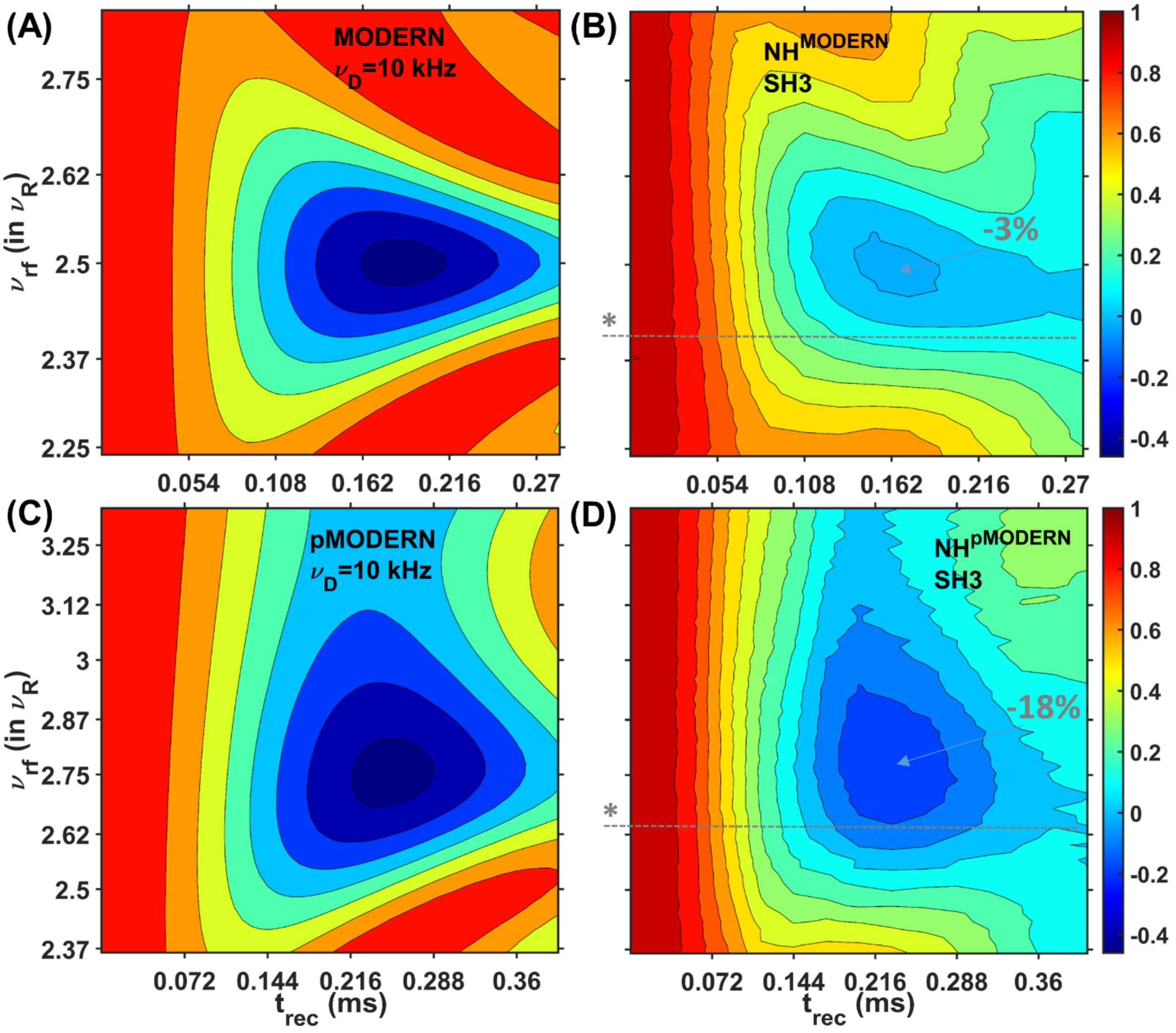

Figure S2 Simulated (A and C) and experimental (B and D) dipolar recoupling signals are shown as a function of recoupling time (x-axis) and RF-field strength (y-axis, in MAS units) using the MODERN (A-B) and pMODERN (C-D) sequences. Gray dashed lines in (B) and (D) indicate the optimal experimental RF-field values according to the RF-field calibration using a 90° ^{1}H hard pulse. In the simulations, a two-spin system with a 10 kHz dipolar coupling

value was considered. In the experiments, 1D (HCAN)H SH3 signals were acquired using MODERN and pMODERN sequences applied to the NH dipolar interaction. The MAS was 55.555 kHz.

Figure S3A-C 1A shows the distribution of ξ values as a function of β for ideal (A) and two different projection angle values - 0° (B) and 120° (C). Figure S3D-E depicts 2D (H)NH sequences with sequential recoupling of the same NH dipolar coupling (using pMODERN). To transfer the signal from ^{1}H to ^{15}N and back, either linear ramped CP elements[2] (Figure S3D) or refocused INPET[3] (Figure S3E) were used. The experimental curves are shown in Figures 1C and 1D in the main text and in Figure S4.

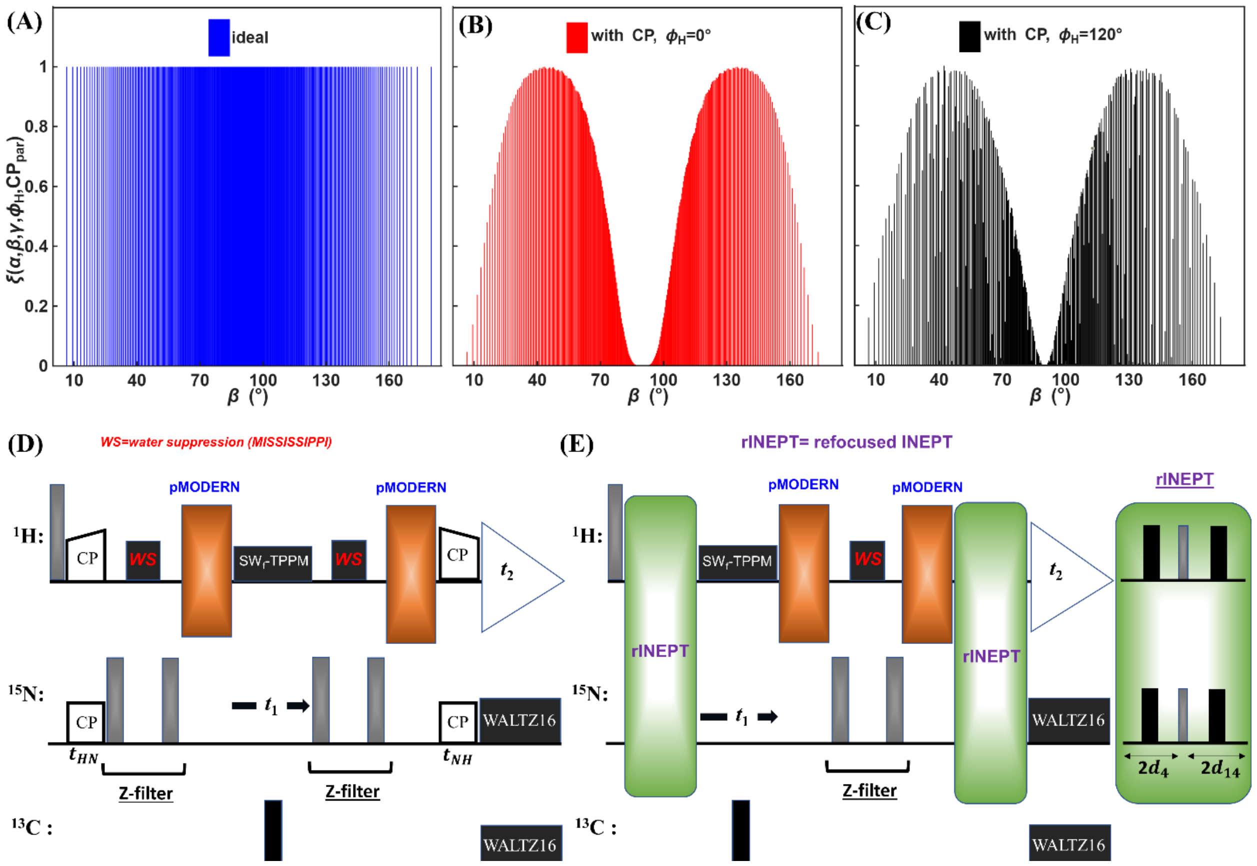

Figure S3 (A)-(C) The amplitude of an individual orientation as a function of angle β in ideal case (A, blue) and in the case where ramped CP blocks are used for H1→N1/N2 and N1/N2→H2 transfers, with projection angle values of $\phi_{proj} = 0°$ (B, red) and $\phi_{proj} = 120°$ (C, black) between the H1-N1/N2 and N1/N2-H2 dipolar interactions.

(D)-(E) 2D (H)NH sequences using linear ramped CP elements (D) and refocused INEPT elements (E) were employed for the ^{1}H→^{15}N and ^{15}N→^{1}H transfers. In each sequence, pMODERN was applied sequentially to the same NH interaction, with a Z-filter (a water suppression element, MISSISSIPPI) placed in the middle. For CP elements, linear ramps of [80:100] (in %) and [100:80] (in %) were used for the ^{1}H→^{15}N and ^{15}N→^{1}H transfers. Heteronuclear dipolar interactions were decoupled using SW_f-TPPM,[4] (proton channel) and WALTZ-16,[5] (nitrogen and carbon channels).

All experiments were performed on an 800 MHz spectrometer at a 55.555 kHz MAS. The optimal RF-field strength for the pMODERN sequence was determined by acquiring 1D (HCAN)H spectra with a fixed pMODERN recoupling time of 0.201 ms, while varying the pMODERN RF-field power. The optimal RF-field strength corresponded to the point at which the 1D (HCAN)H signal intensity was at a minimum.

For experiments using CP elements (Figure S3A), a water suppression block (MISSISSIPPI)[6] was split into two parts: 20% of the total duration (24 m) placed after the first CP element and 80% (96 ms duration) placed after t_1 encoding, for a total time of 120 ms (13.95 kHz RF-field strength). The goal was to eliminate any two-spin operators excited by the first CP element. For experiments using refocused INEPT elements, the optimal d_4 and d_{14} delay times (shown in Figure S3B) were determined from 1D (HN)H experiments by identifying the conditions that yielded the maximum signal intensity. Table S1 summarizes the key experimental parameters used in each experiment.

	**^{1}H→^{15}N transfer**			**^{15}N→^{1}H transfer**			**Number Scans**	**Total Time**
**CP elements**	**^{1}H**	**^{15}N**	$\boldsymbol{t_{HN}}$	**^{1}H**	**^{15}N**	$\boldsymbol{t_{HN}}$	16	0.875 h
	[82:103] (in kHz)	42 kHz	0.72 ms	[103:82] (in kHz)	42 kHz	0.63 ms		

**refocused INEPT**	$\boldsymbol{d_4}$	$\boldsymbol{d_{14}}$	$\boldsymbol{d_4}$	$\boldsymbol{d_{14}}$	128	7 h
	1.6 ms	2.3 ms	2.3 ms	1.6 ms		

Table S1 Summary of the key parameters used in 2D (H)NH experiments with linear ramped CP elements and refocused INEPT elements.

Figure S4A-G compare six SH3 experimental TA curves with $\phi_H = 0°$, obtained using linear ramped CP elements (red) and refocused INEPT elements (blue) for the ^{1}H→^{15}N and ^{15}N→^{1}H transfers in the (H)NH-based sequence. With the exception of certain side-chain peaks (see TA curves in Figures 1C-D in the main text), the initial amplitudes are significantly weaker with refocused INEPT compared to linear ramped CP elements. As an example, 2D (H)NH spectra acquired with zero pMODERN recoupling time are shown in Figure S4H. Even with 8 times more scans (128 via 16, Table S1), only a few backbone nitrogen peaks are detected in the blue spectrum.

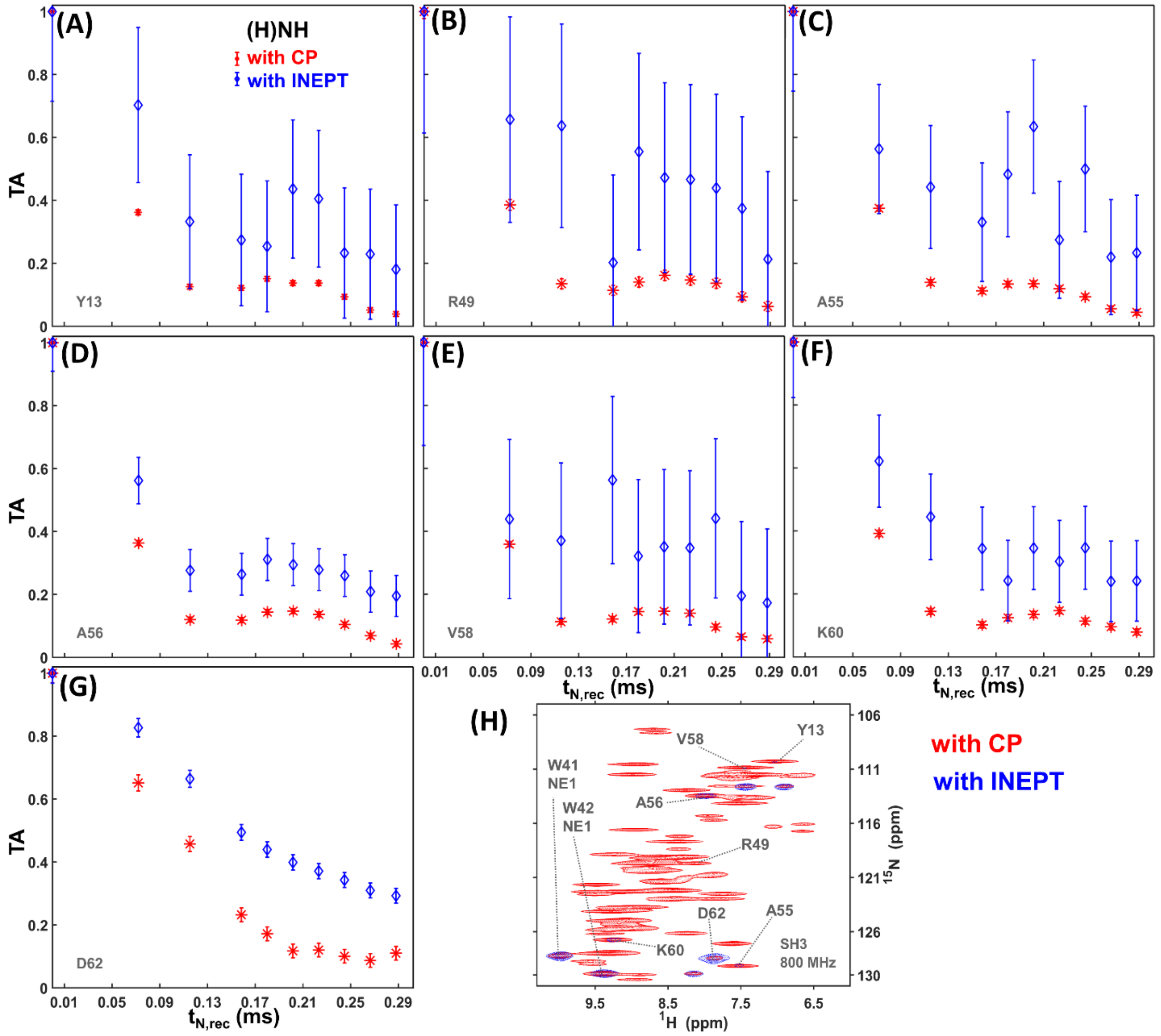

Figure S4 (A)-(G) Six experimental SH3 curves obtained using refocused INEPT (blue diamonds) and ramped CP (red stars) for the $^{1}H\rightarrow^{15}N$ and $^{15}N\rightarrow^{1}H$ transfers. In each case, the pMODERN sequence was applied twice in succession to recouple the ^{15}N-^{1}H dipolar interaction. (H) 2D (H)NH spectra acquired with refocused INEPT (blue) and CP (red). The experimental parameters are summarized in Table S1. Data were recorded on an 800 MHz spectrometer with a 55.555 kHz MAS.

The simulated spin system, FLAN conditions and additional 1D data

FLAN conditions[7] represent an alternative method for defining the matching conditions using the flip angles of applied RF-field pulses, under which the maximum transfer (in absolute value) between a pair of dipolar-coupled spins occurs.

For Hartmann-Hahn conditions,[8] the simultaneously applied RF-field pulses are considered as spin-lock pulses with RF-field strengths of ν_I and ν_S. Maximum transfer is observed when the sum or difference of the applied RF-field strengths matches either one or two times MAS rate.

In contrast, FLAN conditions treat the applied RF-field pulses as a train of windowed rotor-synchronized pulses, with flip angles of $\alpha_I = 2\pi\nu_I\tau_I$ and $\alpha_S = 2\pi\nu_S\tau_S$. In the case of continuous RF-field pulses (spin-lock pulses), $\tau_I = \tau_S = T_R$.

The FLAN conditions for zero-quantum (ZQ) and double-quantum transfers are defined by the following equations:

ZQ: $\alpha_I + \alpha_S = \pi + 2\pi n$ and $\alpha_I - \alpha_S = 2\pi + 2\pi n', \quad n \geq n'$, Eqn. (S1A)

DQ: $\alpha_I + \alpha_S = 2\pi + 2\pi n$ and $\alpha_I - \alpha_S = \pi + 2\pi n', \quad n \geq n'$. Eqn. (S1B)

As an example, some optimal flip angle conditions and the corresponding RF-field strength values for continues RF-field pulses are illustrated in Figure S5A. Decreasing the pulse duration on the S spin by a factor of two requires doubling ν_S to maintain the same flip angle value (x-axis in Figure S5B).

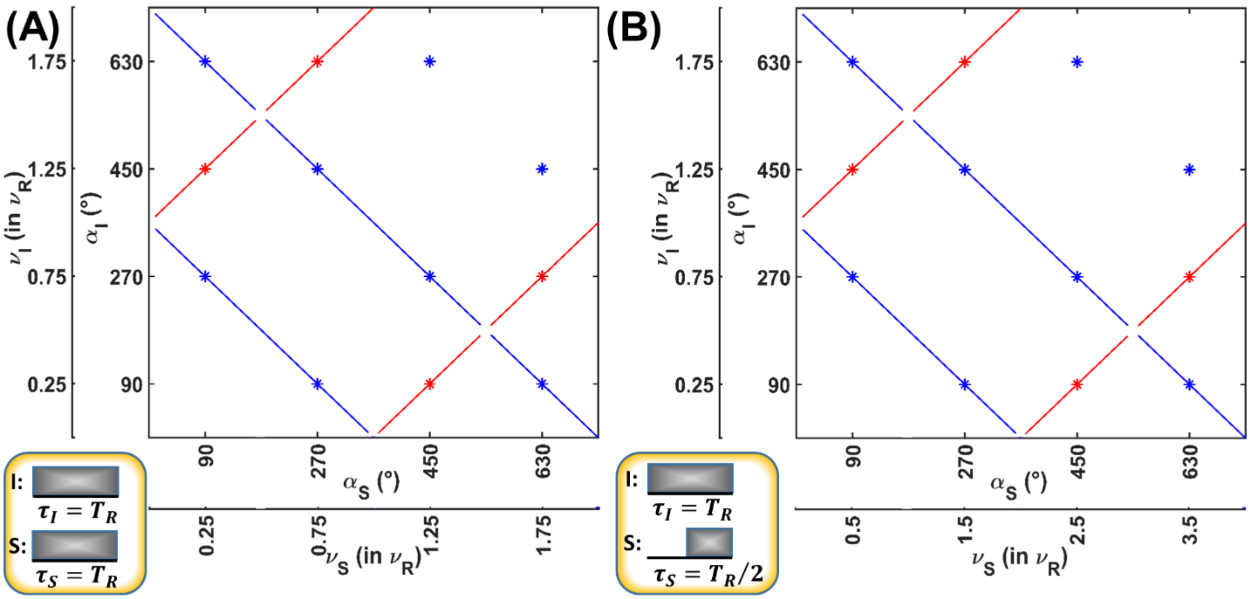

Figure S5 Some optimal flip angle and RF-field strength values (in MAS rate, ν_R) for continuous pulses on each channel (A) and half-windowed pulses on S channel (B). Red and blue stars represent positive and negative conditions, respectively. Red and blue lines represent the flip angles that correspond to the well-established CP conditions, where the sum or difference between the (average) RF-field strengths matches Hartmann-Hahn conditions.[10]

The simulated spin system is depicted in Figure S6A and consists of three aliphatic proton spins (one H_α, two H_β) and one amide proton spin (H), one carbon (C_α) and one nitrogen (N) spin. For CP transfers, the initial signals from all simulated proton spins are transferred to carbon using a linear ramped CP element.[2] Then, the carbon is connected to the nitrogen spin using the SPEPS element.[9] Finally, the signal is transferred from the nitrogen to the amide proton using another linear ramped CP element.

For calculating dipolar couplings and angles between different dipolar interactions, the coordinates of the five spins in a histidine residue were taken from the 2N70 PDB structure.[10] The dipolar coupling values and isotropic chemical shift values used in the simulations are shown in Figure S6B. For pMODERN simulations, a 20 kHz dipolar coupling between C-H spins and a 10 kHz dipolar coupling between N-H spins were used.

The torsion angle, ϕ_H, is defined as the angle between the two planes formed by coordinates of the H_α-C_α-N and C_α-N-H spins. The NMR-determined angles can theoretically be either positive or negative. In the following, we consider only the magnitude of ϕ_H ($|\phi_H|$), since the TA curve cannot be used to determine the sign; therefore, $|\phi_H|=\phi_H$.

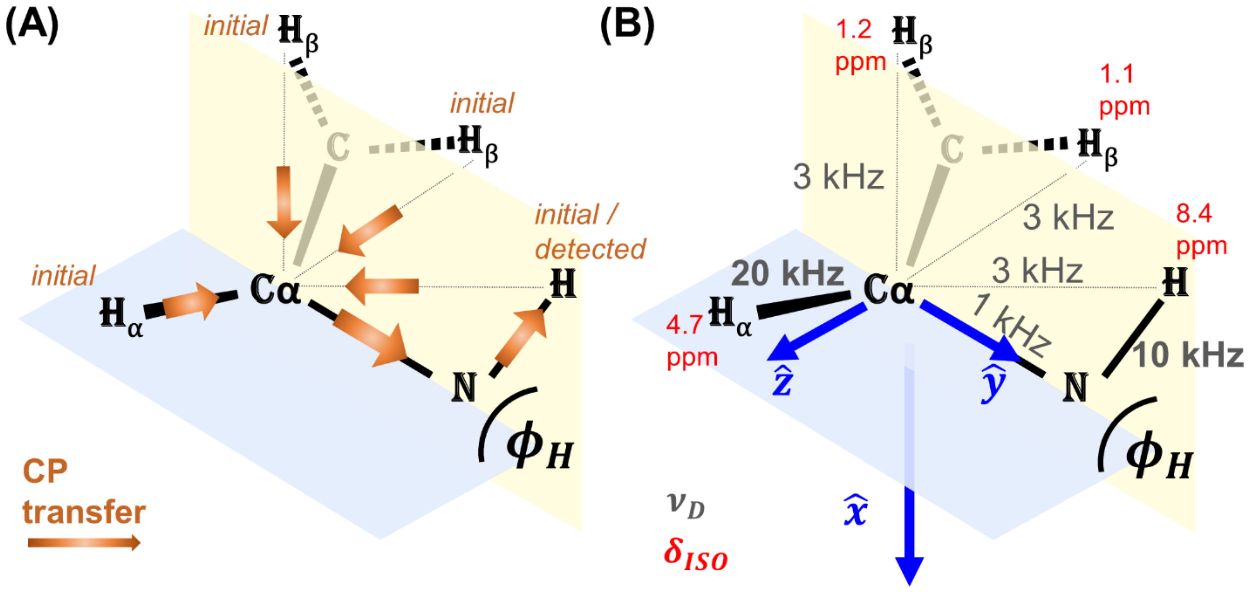

Figure S6 The simulated spin system consists of six spins in total: four proton spins (one H_α, two H_β, one amide H), one carbon (C_α) and one nitrogen (N) spin. (A) The CP transfer path from the three aliphatic and one amide protons (initial) to the amide proton (detected), via C_α and N spins, is depicted with brown arrows. (B) The dipolar coupling values and isotropic chemical shift values used in the simulations.

Figure S7 presents simulated TA curves at different ϕ_H values, both without (ideal, dashed) and with CP elements (solid) and different CP conditions for N→H transfer with continuous (Figures S7A and S7B) and windowed (Figures S7C and S7D) shapes.

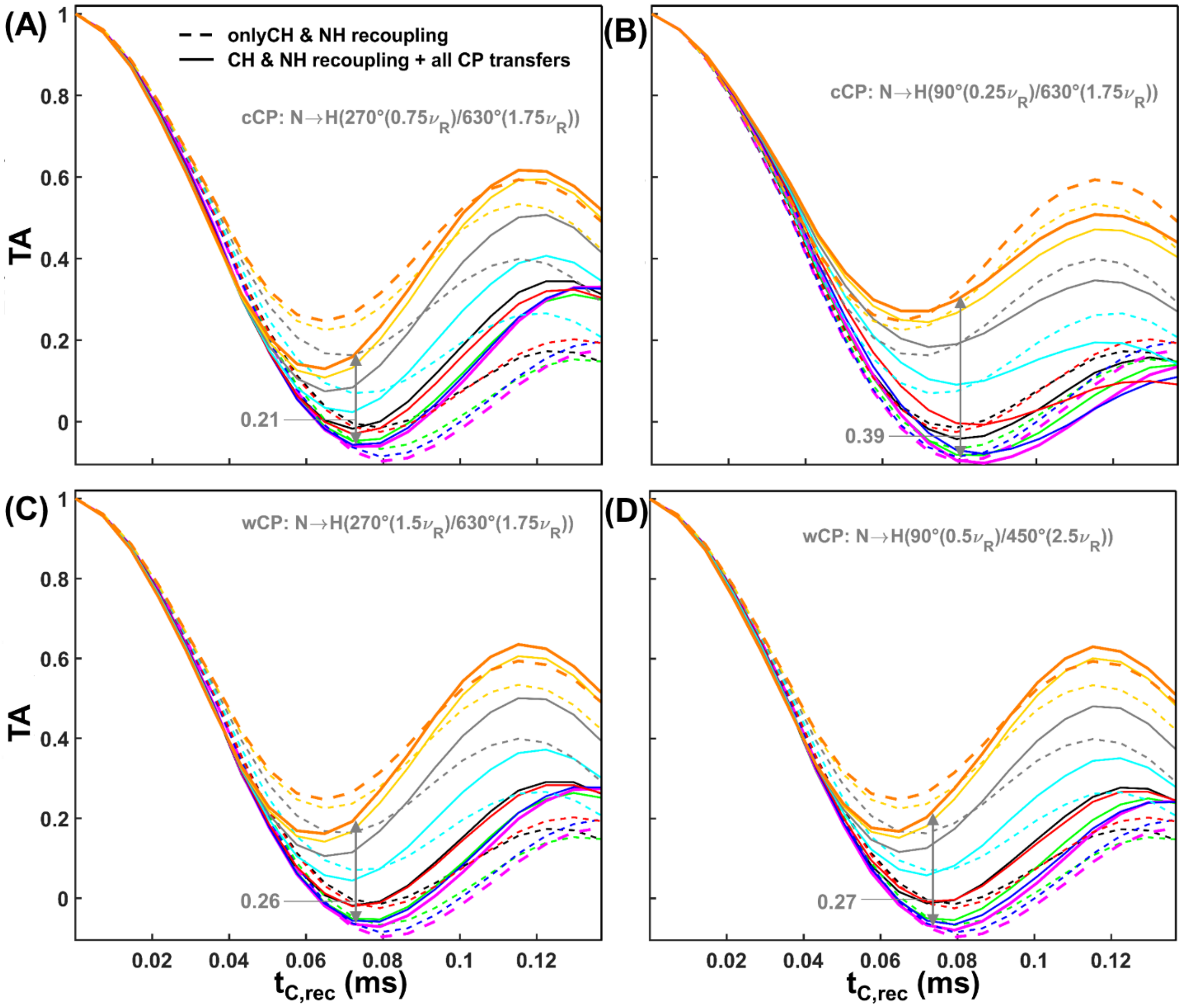

Figure S7 Simulated torsion angle curves in the ideal case (dashed lines) and with continuous (A and B) and windowed (C and D) CP elements (solid lines) with CP conditions of 270°(0.75ν_R)/630°(1.75ν_R) (A), 90°(0.25ν_R)/630°(1.75ν_R) (B), 270°(1.5ν_R)/630°(1.75ν_R) (C) and 90°(0.5ν_R)/450°(2.5ν_R) (D) for the N→H transfer. In the simulations, a six-spin system was considered: one Hα, two Hβ, one Cα, one N and one ^{N}H. In all simulations, only directly bonded spins (Hα for Cα and ^{N}H for N) are taken into account during the pMODERN sequences. For H→C and N→H transfers, the following ramps were applied on the proton channel: an [80:100](%) with a duration of 720 μs for H→C transfer, and a [100:80](%) with a duration of 630 μs for N→H transfer. For the C→N transfer, a SPEPS element was used with a duration of 2.88 ms and RF-field conditions of 0.25ν_R/0.75ν_R. For CP elements, the mentioned values in MAS units represent the RF-field strengths at the midpoint of the applied shapes. The MAS rate was 55.555 kHz.

Figure S8A shows the curves with sequential recoupling of the same dipolar interaction (NH) in the (HCAN)H experiments. All curves exhibit similar behavior, regardless CP conditions for the $^{1}H \rightarrow ^{13}C$ transfer. Figure S8A shows the build-up of (HCAN)H signal as a function of HC contact time.

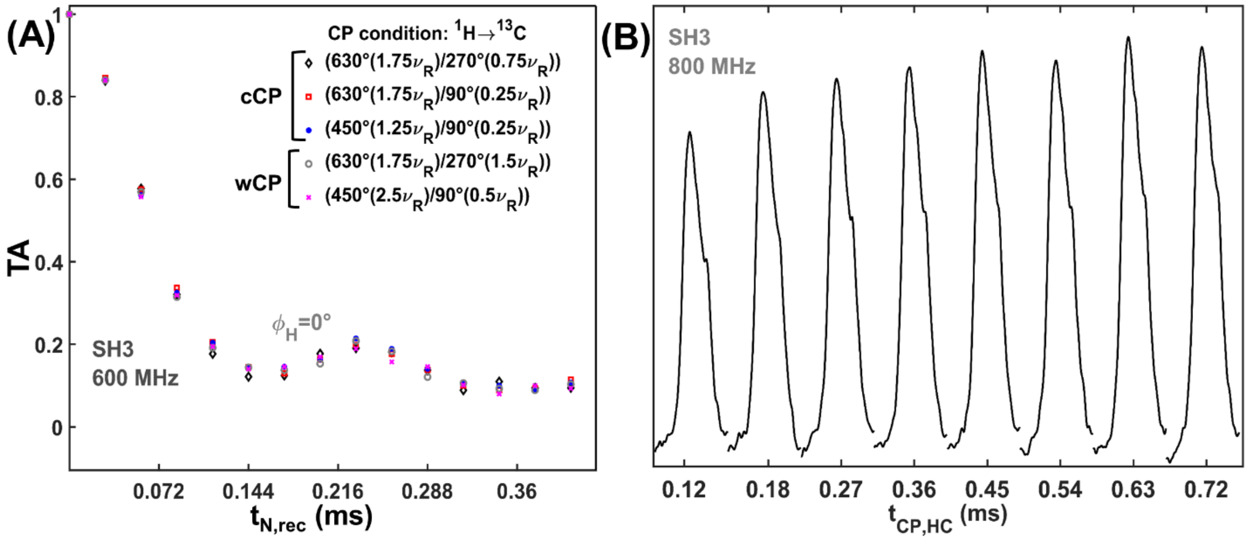

Figure S8 (A) (HCAN)H SH3 torsion angle curves (the pMODERN sequence was applied twice in succession to recouple the ^{15}N-^{1}H dipolar coupling) and (B) (HCAN)H spectra at different HC contact time. (A) The experimental torsion angle curves under different CP conditions for $^{1}H \rightarrow ^{13}C$ transfer with continuous (cCP) and windowed (wCP) shapes. For H→C and N→H transfers, the following ramps were applied on the proton channel: an [80:100](%) with a duration of 720 μs for H→C transfer, and a [100:80](%) with a duration of 630 μs for N→H transfer. For the $^{15}N \rightarrow ^{1}H$ transfer, RF-field conditions of $0.75\nu_R/1.75\nu_R$ (continuous CP) were used. For the $^{13}C \rightarrow ^{15}N$ transfer, a SPEPS element was used with a duration of 2.88 ms and RF-field conditions of $0.25\nu_R/0.75\nu_R$. For CP elements, the mentioned values in MAS units represent the RF-field strengths at the midpoint of the applied shapes. Data were recorded on an 600 MHz spectrometer with 55.555 kHz MAS. (B) The duration of the H→C ramped CP was varied. For the $^{15}N \rightarrow ^{1}H$ transfers, the windowed CP condition of $90°(0.5\nu_R)/450°(2.5\nu_R)$ was used. Data were recorded on an 800 MHz spectrometer.

Fitting procedure

The experimental signal, S_{exp}, is compared with the simulated signal, S_{sim}, in the time domain by minimizing the value of the residual, χ_ν^2:

$$\chi_\nu^2 = \left[sum\left[\left(S_{exp}(t_{rec}) - S_{sim}(x; t_{rec})\right)/error\right]^2\right]/(\nu - 1), \qquad \text{Eqn. (S2)}$$

where, x is the parameter obtained (a dipolar coupling value or torsion angle), ν is the number of the experimental points; *error* – is the uncertainty in the peak amplitude, estimated from the RMSD of a noise region of the spectrum.

For a better fit between experimental and simulated signals, two non-structural parameters are involved in the simulations. Both parameters are obtained from the comparison of experimental and simulated Dip curves and then are used for obtaining torsion angle values.

The first parameter, $T_{2,eff}$, has inverse time units. The second parameter is used to define a Gaussian distribution of ν_{rf}, originally proposed to take into account RF inhomogeneity, and now also proposed to approximate the effects of orientation-dependent CP intensity. The Gaussian distribution was truncated at 3.6 percent, and $\Delta\alpha_{rf,max}$ represents the maximum deviation from the reference flip angle, normalized by the reference flip angle. $\Delta\alpha_{rf,max}$ is chosen based on the best agreement between the experimental and simulated curves for each residue. For example, with $\Delta\alpha_{rf,max}$=0.2 and reference $\nu_{rf} = 2.75\nu_R$, the Gaussian distribution goes from $2.75\nu_R$ down to $2.75\nu_R$*0.8=2.2ν_R. While the first parameter is a global parameter (the same for all curves), the second varies depending on the type of recoupled spin pair (CH or NH) as well as CP conditions used.

The simulated Dip signal is described with the following equation:

$$Dip_{sim}(\nu_D; t_{rec}) = \sum_{i=1}^{N} \bar{P}(\Delta\alpha_{rf,i})\, e^{\{-t_{mix}/T_{2,eff}\}} \int \mathrm{d}\Omega\, \mathrm{s}(\Omega, \nu_D; \Delta\alpha_{rf,i}; t_{rec}), \qquad \text{Eqn. (S3)}$$

where the integration over orientation (Ω) represents powder averaging using Euler angles, (α, β, γ).[11] The functions $\mathrm{s}(\Omega, \nu_D; \Delta\alpha_{rf,i}; t_{rec})$ and are recoupled orientation-dependent signals at the end of the pMODERN recoupling sequence with recoupling times t_{rec} and deviated flip angle from the ideal one: $\Delta\alpha_{rf,i} = 1 - i * \Delta\alpha_{rf,max}/N$ and $i = 0,1,..,N-1$. The $T_{2,eff}$ is an effective relaxation time and $\bar{P}(\Delta\alpha_{rf,i})$ is the normalized Gaussian weight factor for $\Delta\alpha_{rf,i}$ value. In ideal case, $\Delta\alpha_{rf,max} = 0$ and $T_{2,eff} \to \infty$.

As an example, Figure S9A shows eleven simulated Dip curves (dashed gray lines) obtained with different deviated normalized flip angle values, ranging from $\Delta\alpha_{rf,1} = 0$ ($\nu_{rf} = 2.75\nu_R$) to $\Delta\alpha_{rf,11} = 0.2$ ($\nu_{rf} = 2.2\nu_R$), with N=11. The solid lines represent their sum with a Gaussian weight factor (shown in Figure S9B).

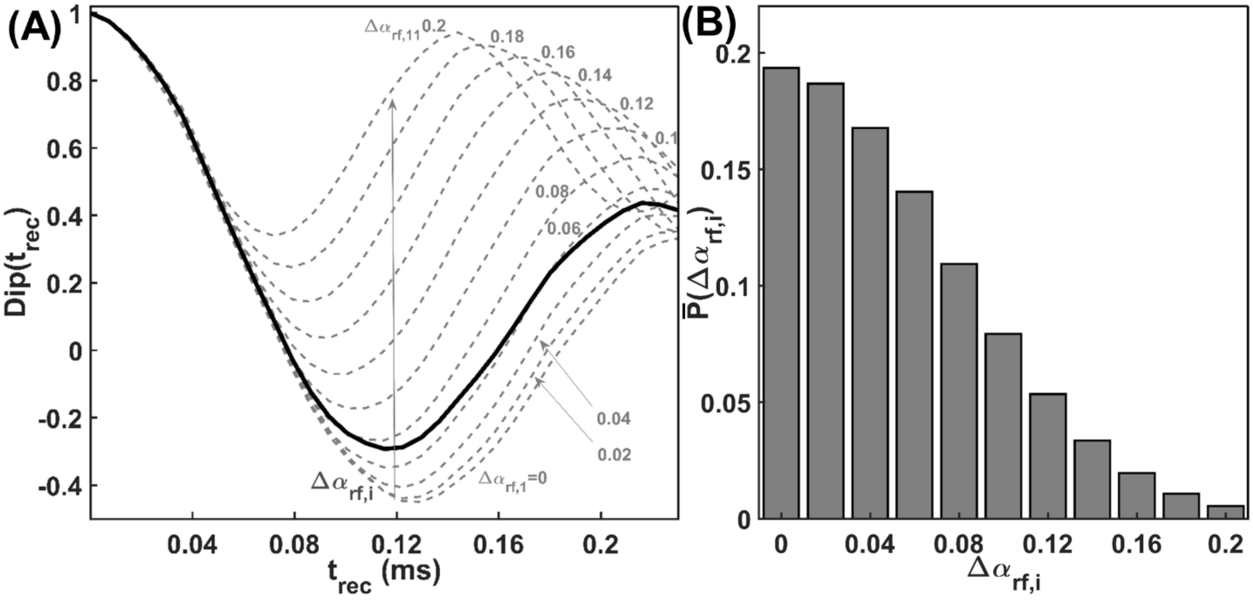

Figure S9 (A) The simulated Dip curves with different deviated normalized flip angle values / rf-field strengths: 0 / $2.75\nu_R$, 0.02 / $2.695\nu_R$, 0.04 / $2.64\nu_R$, 0.06 / $2.585\nu_R$, 0.08 / $2.53\nu_R$, 0.1 / $2.475\nu_R$, 0.12 / $2.42\nu_R$, 0.14 / $2.365\nu_R$, 0.16 / $2.31\nu_R$, 0.18 / $2.255\nu_R$ and 0.2 / $2.2\nu_R$. The solid line represents their sum with a Gaussian weight factor,

whose distribution is shown in (B). The simulations were performed for a two-spin system with a 20 kHz dipolar coupling value and at a 55.555 kHz MAS.

The simulated TA signal is described with the following equation:

$$TA_{sim}(\nu_{D,CH}, \nu_{D,NH}; t_{1,rec}, t_{2,rec}; \phi_H) = \quad \text{Eqn. (S4)}$$

$$\sum_{i=1}^{N} \bar{P}(\Delta\alpha_{rf,i})\, e^{\{-t_{mix}/T_{2,eff}\}} \int \mathrm{d}\Omega\, \mathrm{s}(\Omega, \nu_{\mathrm{D,HC}}, \Delta\alpha_{rf,i}^{CH}, t_{1,\mathrm{rec}})\mathrm{s}(\phi_H, \Omega, \nu_{\mathrm{D,HN}}, \Delta\alpha_{rf,i}^{NH}, t_{2,\mathrm{rec}}),$$

where $\Delta\alpha_{rf,max}^{CH} \neq \Delta\alpha_{rf,max}^{NH}$.

For the pMODERN simulations, exactly the same steps were used to analyze the experimental data as was for the data obtained with the MODERN sequence.[12] The only difference is that the $\Delta\alpha_{rf,max}$ value is not fixed, as it was for the MODERN pulses, but instead determined for each dipolar-recoupled curve. In this way, the remaining influence of CP elements is taken into account, which increases the accuracy of torsion angle determination through comparison of experimental and torsion angle curves. $T_{2,eff}$ remains fixed for all Dip and TA curves from the same dataset.

The distribution of $\Delta\alpha_{rf,max}$ values over CH and NH dipolar couplings for SH3 and S31N M2 under different CP conditions and external magnetic fields is summarized in Figure S10 and S11, respectively. The Table S2 summarizes $T_{2,eff}$ values:

	SH3<br>600 MHz<br>270°(0.75ν_R)/<br>630°(1.75ν_R)	SH3<br>600 MHz<br>270°(1.5ν_R)/<br>630°(1.75ν_R)	SH3<br>800 MHz<br>90°(0.5ν_R)/<br>450°(2.5ν_R)	S31N M2<br>600 MHz<br>270°(1.5ν_R)/<br>630°(1.75ν_R)	S31N M2<br>600 MHz<br>90°(0.5ν_R)/<br>450°(2.5ν_R)
$T_{2,eff}$	0.468 ms	0.353 ms	0.566 ms	0.555 ms	0.596 ms

Table S2 The summary of $T_{2,eff}$ values for each sample and experimental conditions, found from comparison three chosen NH experimental curves with the best performance.

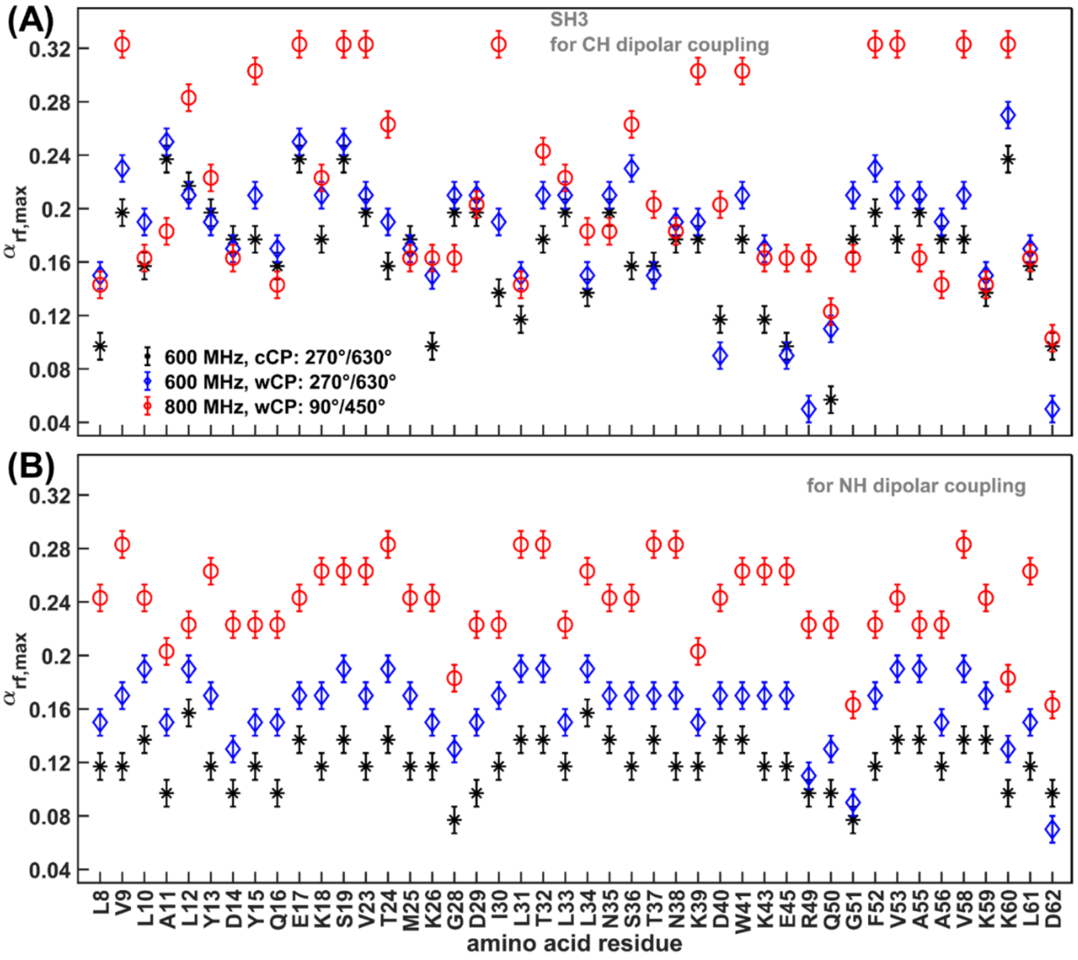

Figure S10 The experimental $\Delta\alpha_{rf,max}$ values as function of amino acid residue in SH3, obtained by comparing simulated and experimental CH (A) and NH (B) Dip curves. The black stars represent data acquired on a 600 MHz with a continuous CP shape for the $^{15}N\rightarrow^{1}H$ transfer: 270°(0.75ν_R)/ 630°(1.75ν_R). The blue diamonds represent data acquired on a 600 MHz with a windowed CP shape for the $^{15}N\rightarrow^{1}H$ transfer: 270°(1.5ν_R, half window)/ 630°(1.75ν_R). The red circles represent data acquired on a 800 MHz with windowed CP shapes for the $^{15}N\rightarrow^{1}H$ transfer: 90°(0.5ν_R, half window)/ 450°(2. 5ν_R, half window). The remaining experimental parameters were identical.

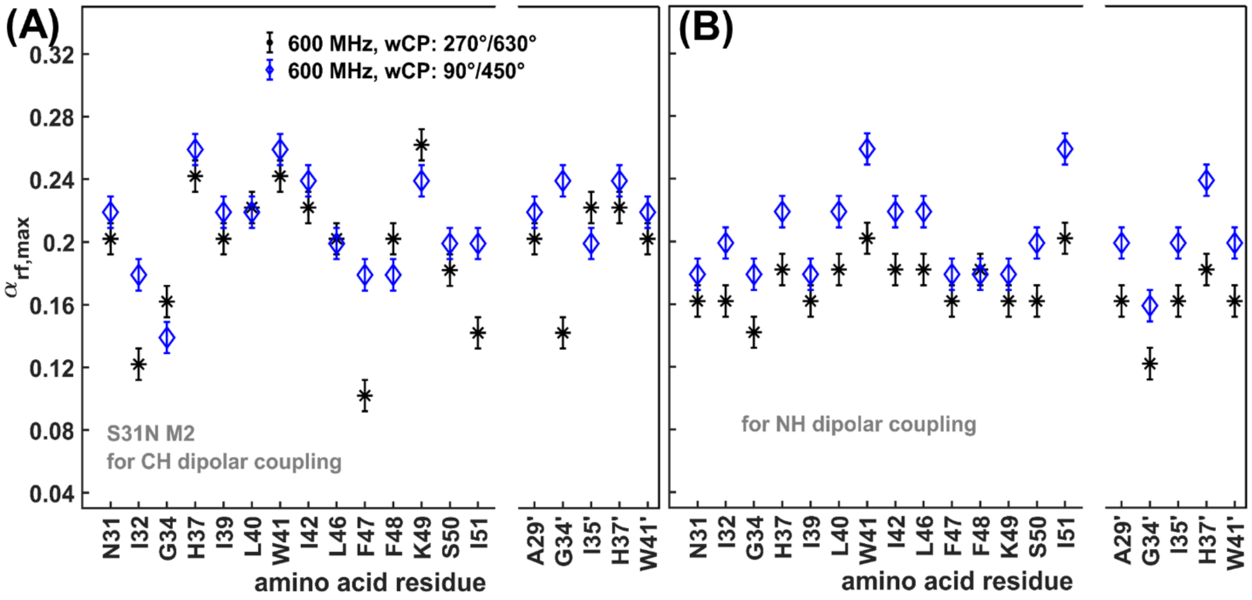

Figure S11 The experimental $\Delta\alpha_{rf,max}$ values as function of amino acid residue in S31N M2, obtained by comparing simulated and experimental CH (A) and NH (B) Dip curves. The black stars represent data acquired with a windowed CP shape for the $^{15}N \rightarrow ^{1}H$ transfer: 270°(1.5ν_R, half window)/ 630°(1.75ν_R). The blue diamonds represent data acquired with windowed CP shapes for the $^{15}N \rightarrow ^{1}H$ transfer: 90°(0.5ν_R, half window)/ 450°(2. 5ν_R, half window). All data were recorded on a 600 MHz spectrometer. The remaining experimental parameters were identical.

Dipolar and torsion angle values in SH3 and S31N M2 samples

Figure S12 summarizes CH and NH dipolar coupling values in the SH3 sample (44 amino acid residues), obtained under three different CP conditions and at different external magnetic fields. A broad distribution of CH and NH dipolar coupling values is observed when the three datasets are compared in kHz (Figures S12A and 12B). However, better agreement between the datasets is achieved when each set is normalized to its own maximum value (Figures S12C and 12D).

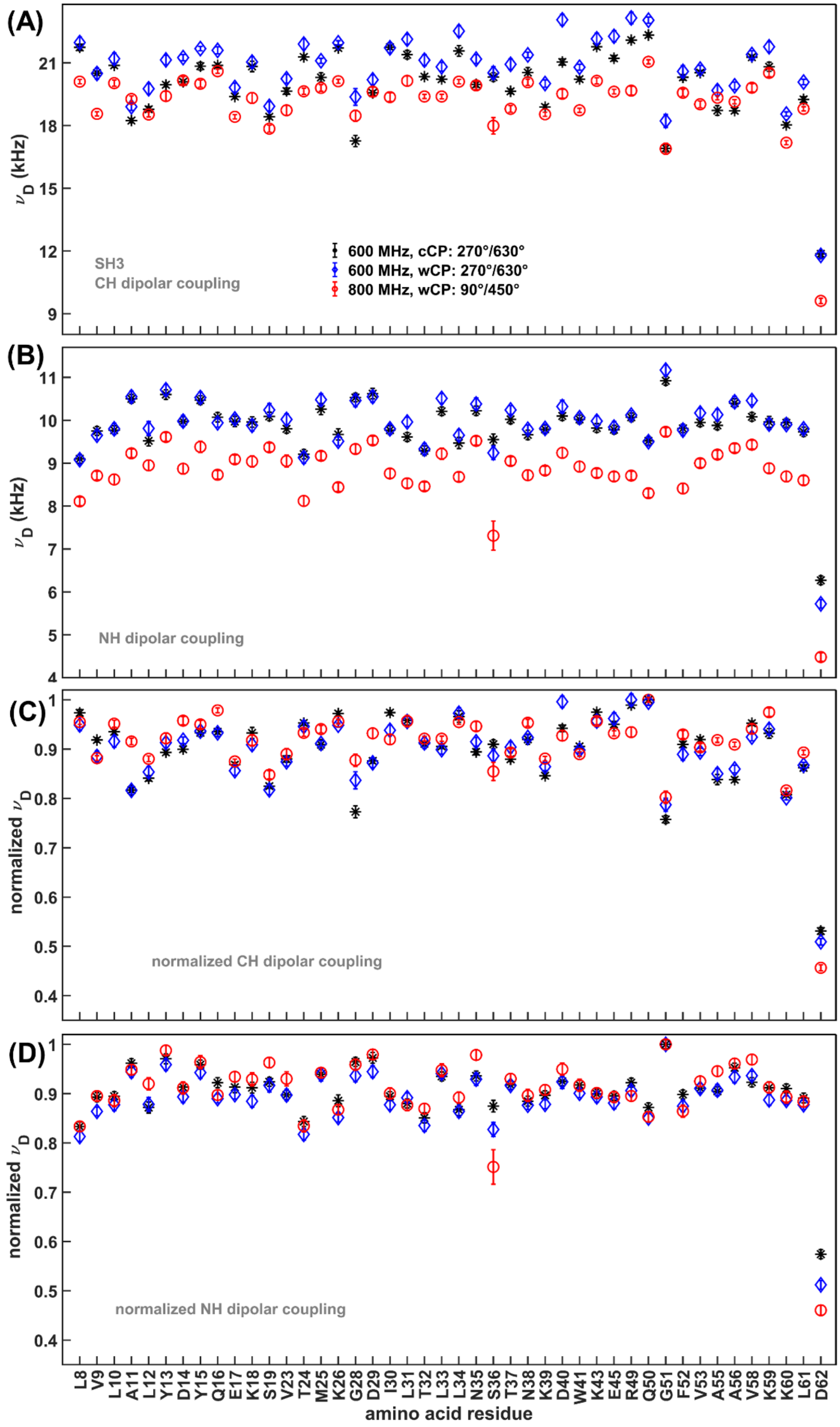
(A)
(B)
(C)
(D)
ν_D (kHz)
normalized ν_D
SH3
CH dipolar coupling
600 MHz, cCP: 270°/630°
600 MHz, wCP: 270°/630°
800 MHz, wCP: 90°/450°
NH dipolar coupling
normalized CH dipolar coupling
normalized NH dipolar coupling
L8 V9 L10 A11 L12 Y13 D14 Y15 Q16 E17 K18 S19 V23 T24 M25 K26 G28 D29 I30 L31 T32 L33 L34 N35 S36 T37 N38 K39 D40 W41 K43 E45 R49 Q50 G51 F52 V53 A55 A56 V58 K59 K60 L61 D62
amino acid residue

Figure S12 CH (A, C) and NH (B, D) SH3 dipolar coupling values in kHz (A-B) and normalized to the maximum value from the same dataset (C-D). The black stars represent data acquired on a 600 MHz with a continuous CP shape for the $^{15}N \rightarrow ^{1}H$ transfer: 270°(0.75ν_R)/ 630°(1.75ν_R). The blue diamonds represent data acquired on a 600 MHz with a windowed CP shape for the $^{15}N \rightarrow ^{1}H$ transfer: 270°(1.5ν_R, half window)/ 630°(1.75ν_R). The red circles represent data acquired on a 800 MHz with windowed CP shapes for the $^{15}N \rightarrow ^{1}H$ transfer: 90°(0.5ν_R, half window)/ 450°(2.5ν_R, half window). The remaining experimental parameters were identical.

Figure S13 summarizes all torsion angle values obtained under three different CP conditions. The comparison between the experimental and simulated curves always yields at least two possible ϕ_H values. However, the ϕ_H values below 90° can be excluded.

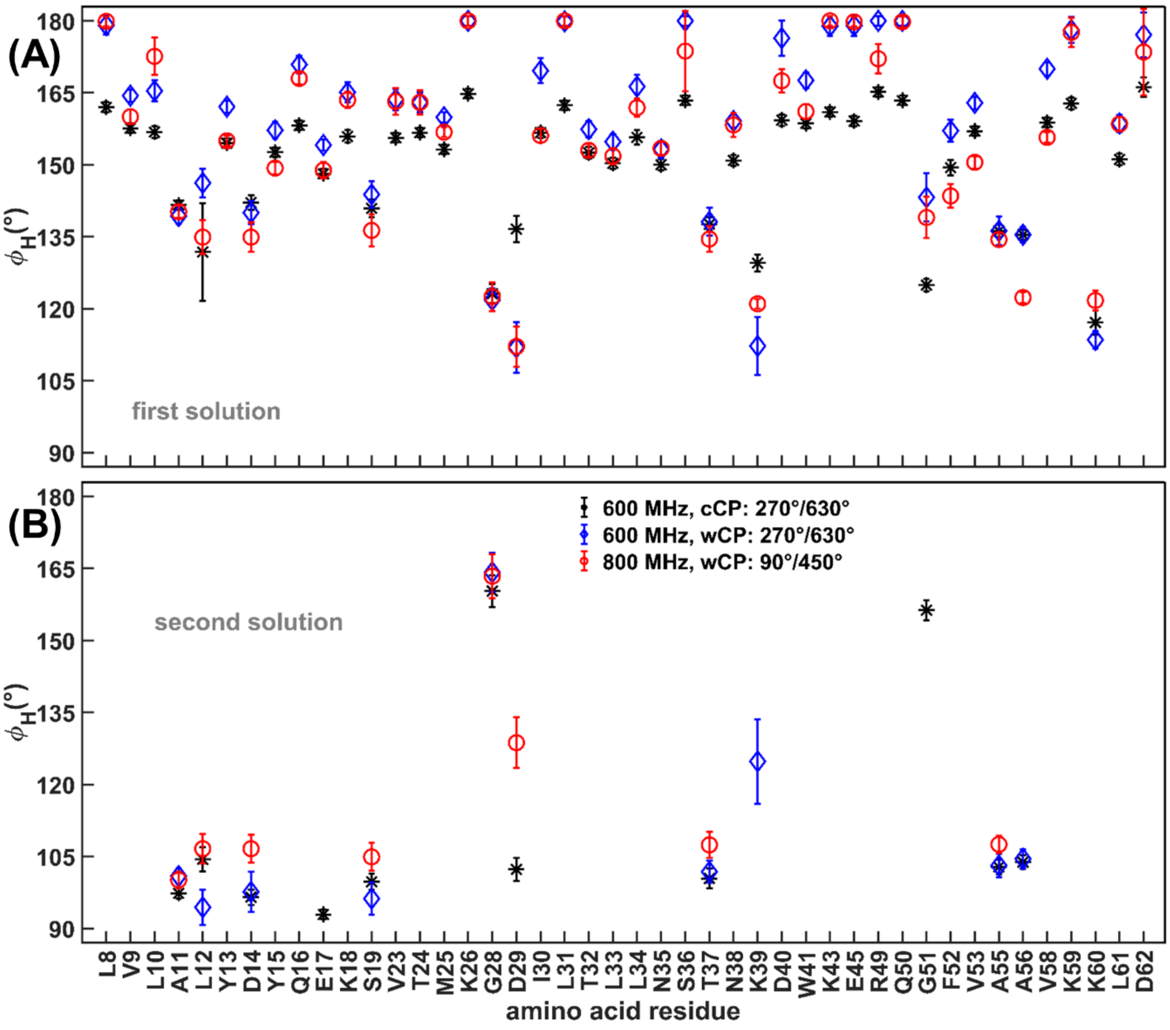

Figure S13 The first (A) and second (B) possible ϕ_H values obtained from the comparison between experimental and simulated torsion angle curves. The black stars represent data acquired on a 600 MHz with a continuous CP shape for the $^{15}N \rightarrow ^{1}H$ transfer: 270°(0.75ν_R)/ 630°(1.75ν_R). The blue diamonds represent data acquired on a 600 MHz with a windowed CP shape for the $^{15}N \rightarrow ^{1}H$ transfer: 270°(1.5ν_R, half window)/ 630°(1.75ν_R). The red circles represent data acquired on a 800 MHz with windowed CP shapes for the $^{15}N \rightarrow ^{1}H$ transfer: 90°(0.5ν_R, half window)/ 450°(2. 5ν_R, half window). The remaining experimental parameters were identical.

Figure S14 summarizes CH, NH dipolar coupling values and torsion angle values in S31N M2 sample (14 amino acid residues in the long chain and 5 in the short chain), obtained under two different CP conditions, both windowed.

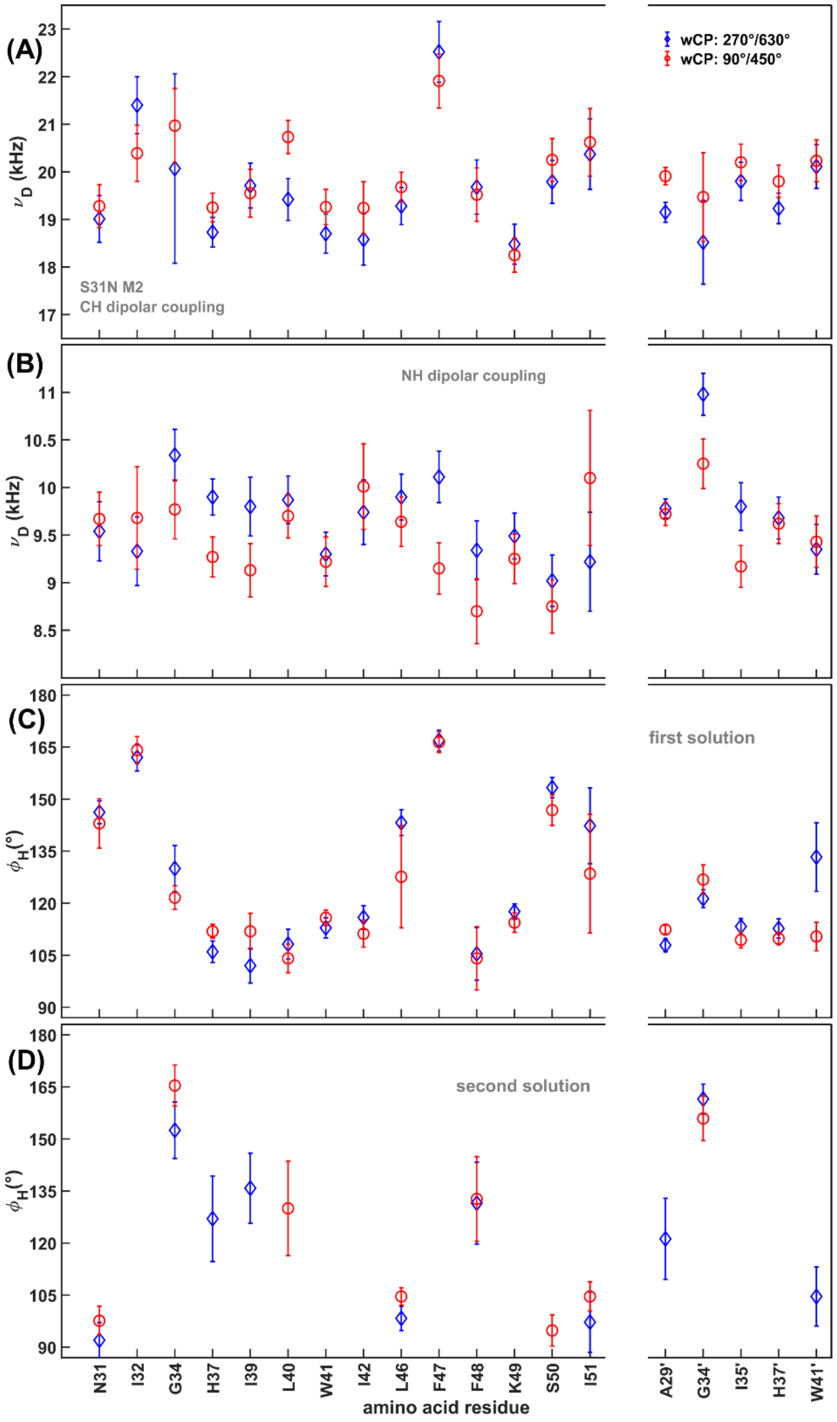

Figure S14 CH (A), NH (B) dipolar coupling values in kHz and torsion angle values (C, D) in S31N M2 sample. The blue diamonds represent data with a windowed CP shape for the $^{15}N \rightarrow ^{1}H$ transfer: 270°(1.5ν_R, half window)/ 630°(1.75ν_R). The red circles represent with windowed CP shapes for the $^{15}N \rightarrow ^{1}H$ transfer: 90°(0.5ν_R, half window)/

450°(2. 5ν_R, half window). All data was acquired on a 600 MHz spectrometer. The remaining experimental parameters were identical.

Dipolar and torsion angle SH3 curves

Figures 15, 16 and 17 show the experimental and simulated Dip CH, Dip NH and TA curves for CP condition with continuous shape: 270°(0.75ν_R)/ 630°(1.75ν_R).

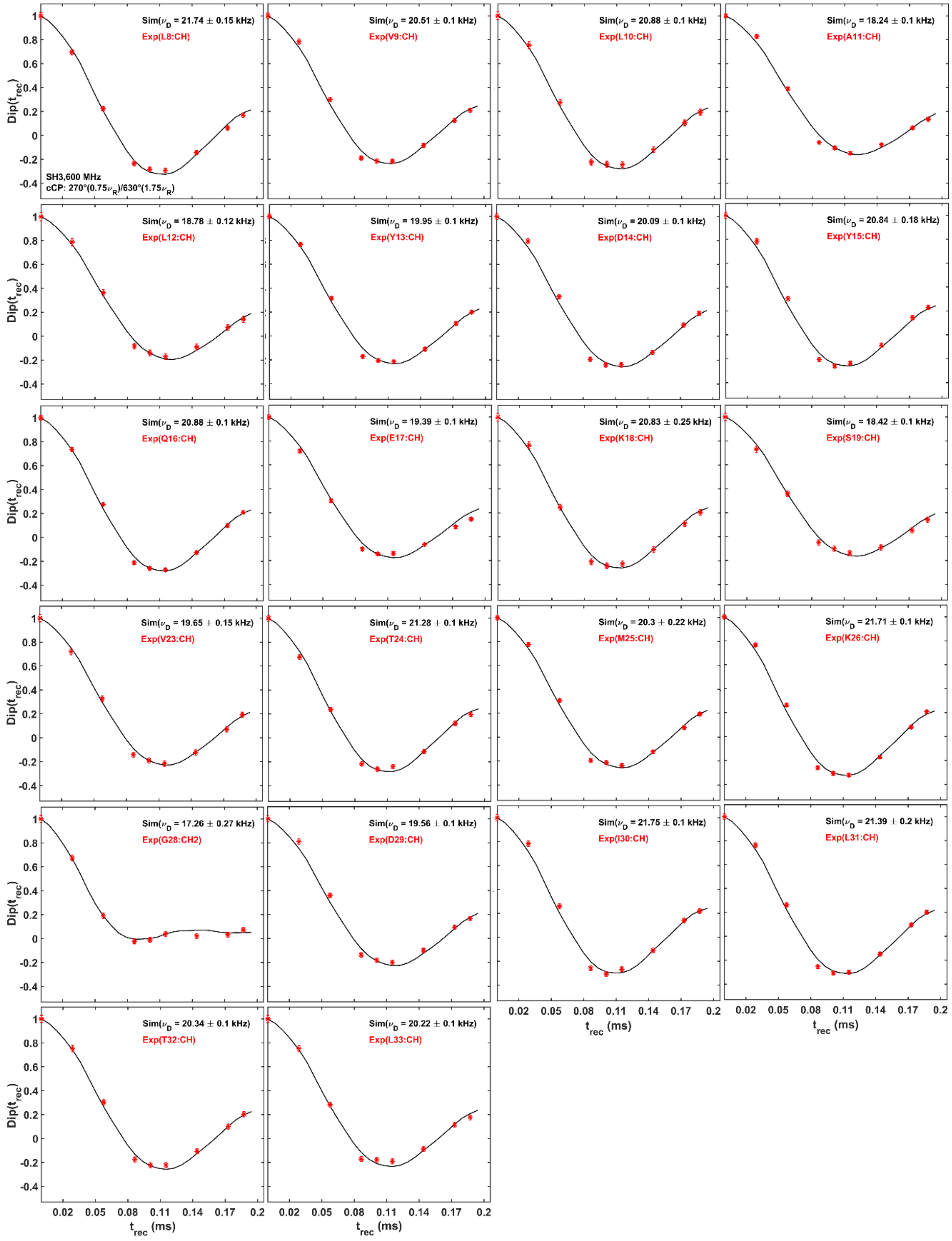
Sim(νD = 21.74 ± 0.15 kHz)
Exp(L8:CH)
SH3,600 MHz
cCP: 270°(0.75νR)/630°(1.75νR)
Sim(νD = 20.51 ± 0.1 kHz)
Exp(V9:CH)
Sim(νD = 20.88 ± 0.1 kHz)
Exp(L10:CH)
Sim(νD = 18.24 ± 0.1 kHz)
Exp(A11:CH)
Sim(νD = 18.78 ± 0.12 kHz)
Exp(L12:CH)
Sim(νD = 19.95 ± 0.1 kHz)
Exp(Y13:CH)
Sim(νD = 20.09 ± 0.1 kHz)
Exp(D14:CH)
Sim(νD = 20.84 ± 0.18 kHz)
Exp(Y15:CH)
Sim(νD = 20.88 ± 0.1 kHz)
Exp(Q16:CH)
Sim(νD = 19.39 ± 0.1 kHz)
Exp(E17:CH)
Sim(νD = 20.83 ± 0.25 kHz)
Exp(K18:CH)
Sim(νD = 18.42 ± 0.1 kHz)
Exp(S19:CH)
Sim(νD = 19.65 ± 0.15 kHz)
Exp(V23:CH)
Sim(νD = 21.28 ± 0.1 kHz)
Exp(T24:CH)
Sim(νD = 20.3 ± 0.22 kHz)
Exp(M25:CH)
Sim(νD = 21.71 ± 0.1 kHz)
Exp(K26:CH)
Sim(νD = 17.26 ± 0.27 kHz)
Exp(G28:CH2)
Sim(νD = 19.56 ± 0.1 kHz)
Exp(D29:CH)
Sim(νD = 21.75 ± 0.1 kHz)
Exp(I30:CH)
Sim(νD = 21.39 ± 0.2 kHz)
Exp(L31:CH)
Sim(νD = 20.34 ± 0.1 kHz)
Exp(T32:CH)
Sim(νD = 20.22 ± 0.1 kHz)
Exp(L33:CH)
Dip(trec)
trec (ms)

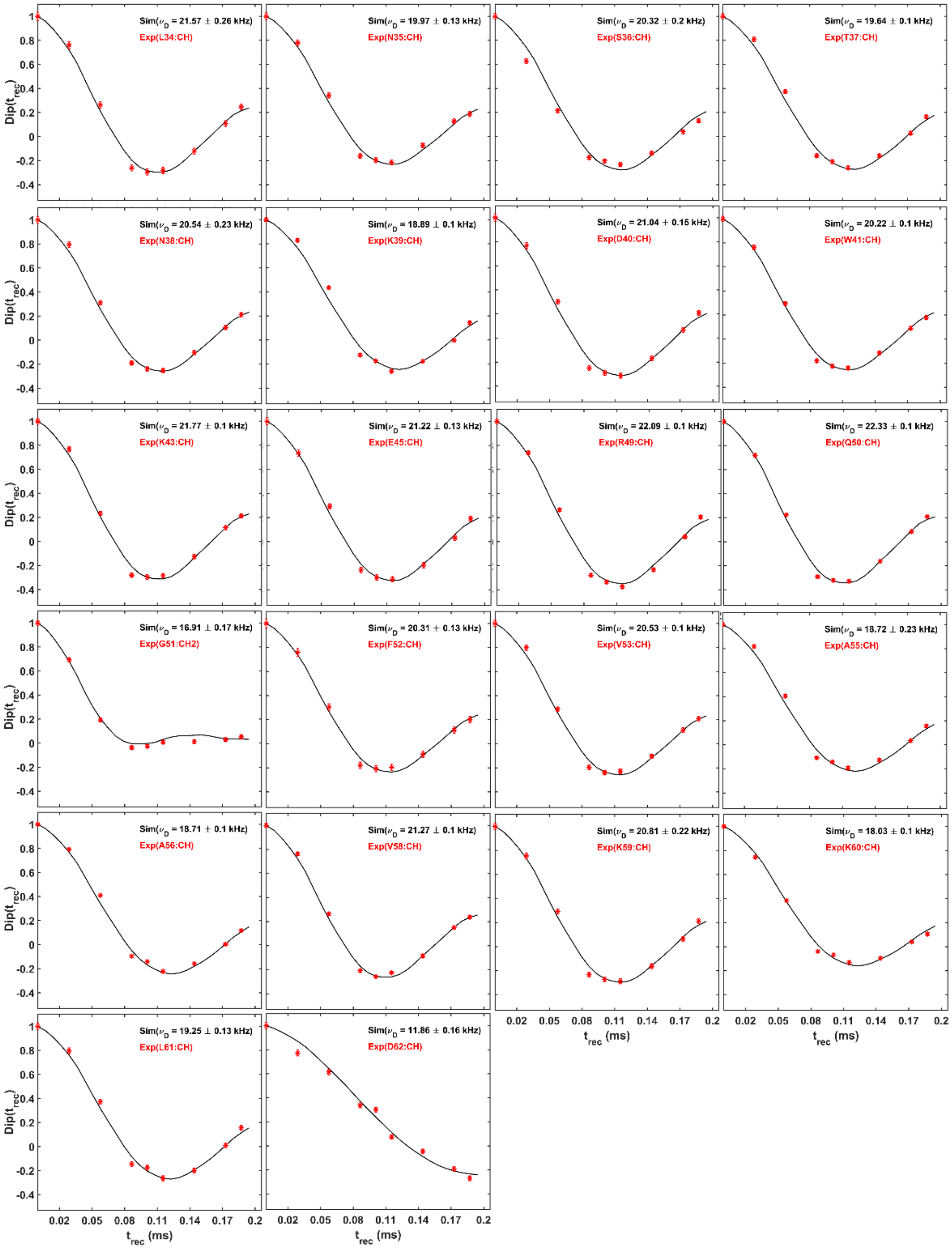

Figure S15 Experimental SH3 (with continuous CP condition of 270°(0.75ν_R)/ 630°(1.75ν_R)) and simulated **Dip curves for HC dipolar coupling values**. The data was acquired on a 600 MHz spectrometer. The simulated pMODERN curves were obtained using the $\Delta\alpha_{rf,max}$ values (summarized in Figure S10A, black stars) and $T_{2,eff} =$ 0.468 ms for better fitting. The fitting errors were obtained by generating 200 Monte Carlo curves, assuming a Gaussian noise distribution (2σ).

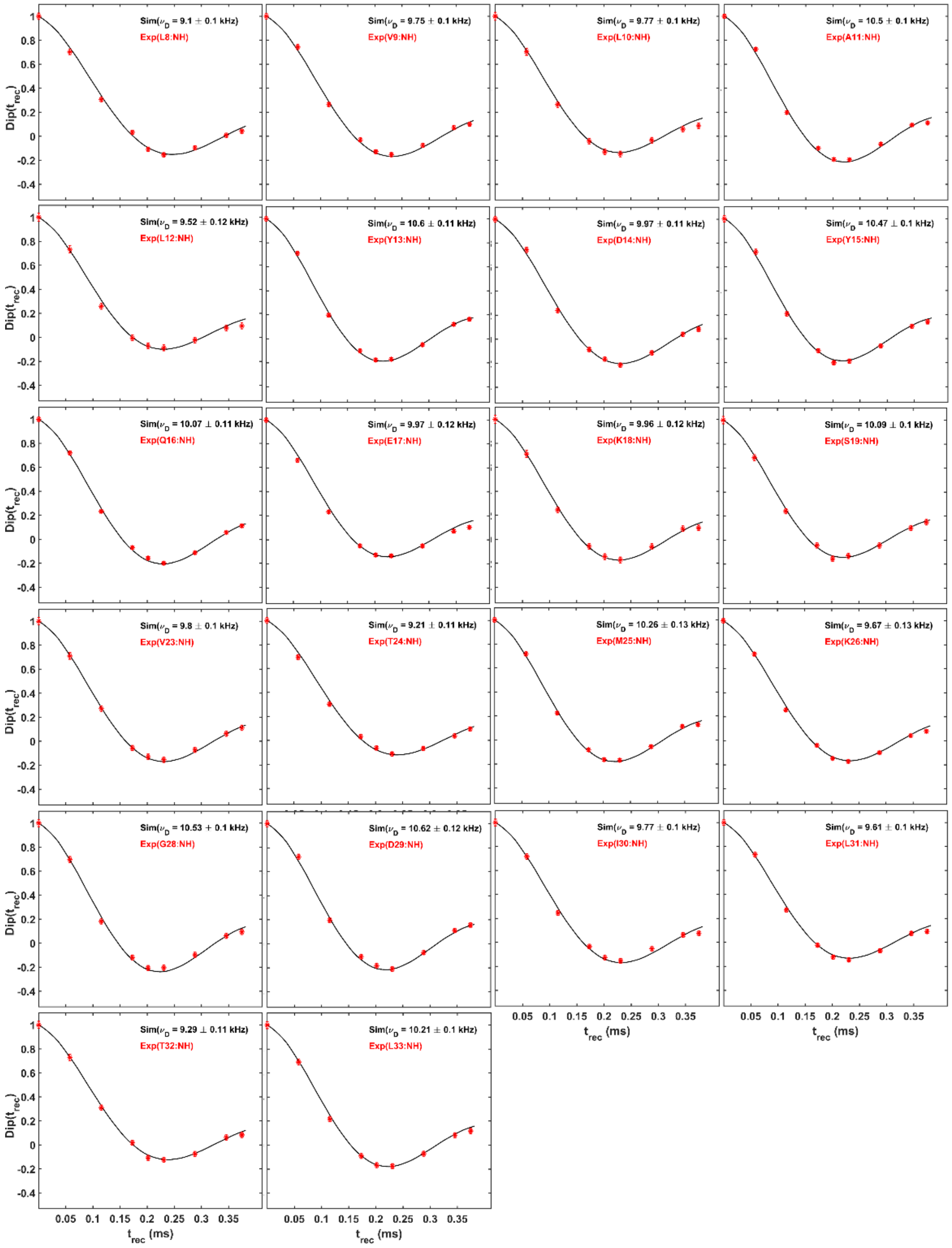

Sim(νD = 9.1 ± 0.1 kHz)
Exp(L8:NH)
Sim(νD = 9.75 ± 0.1 kHz)
Exp(V9:NH)
Sim(νD = 9.77 ± 0.1 kHz)
Exp(L10:NH)
Sim(νD = 10.5 ± 0.1 kHz)
Exp(A11:NH)
Sim(νD = 9.52 ± 0.12 kHz)
Exp(L12:NH)
Sim(νD = 10.6 ± 0.11 kHz)
Exp(Y13:NH)
Sim(νD = 9.97 ± 0.11 kHz)
Exp(D14:NH)
Sim(νD = 10.47 ± 0.1 kHz)
Exp(Y15:NH)
Sim(νD = 10.07 ± 0.11 kHz)
Exp(Q16:NH)
Sim(νD = 9.97 ± 0.12 kHz)
Exp(E17:NH)
Sim(νD = 9.96 ± 0.12 kHz)
Exp(K18:NH)
Sim(νD = 10.09 ± 0.1 kHz)
Exp(S19:NH)
Sim(νD = 9.8 ± 0.1 kHz)
Exp(V23:NH)
Sim(νD = 9.21 ± 0.11 kHz)
Exp(T24:NH)
Sim(νD = 10.26 ± 0.13 kHz)
Exp(M25:NH)
Sim(νD = 9.67 ± 0.13 kHz)
Exp(K26:NH)
Sim(νD = 10.53 ± 0.1 kHz)
Exp(G28:NH)
Sim(νD = 10.62 ± 0.12 kHz)
Exp(D29:NH)
Sim(νD = 9.77 ± 0.1 kHz)
Exp(I30:NH)
Sim(νD = 9.61 ± 0.1 kHz)
Exp(L31:NH)
Sim(νD = 9.29 ± 0.11 kHz)
Exp(T32:NH)
Sim(νD = 10.21 ± 0.1 kHz)
Exp(L33:NH)
Dip(trec)
trec (ms)

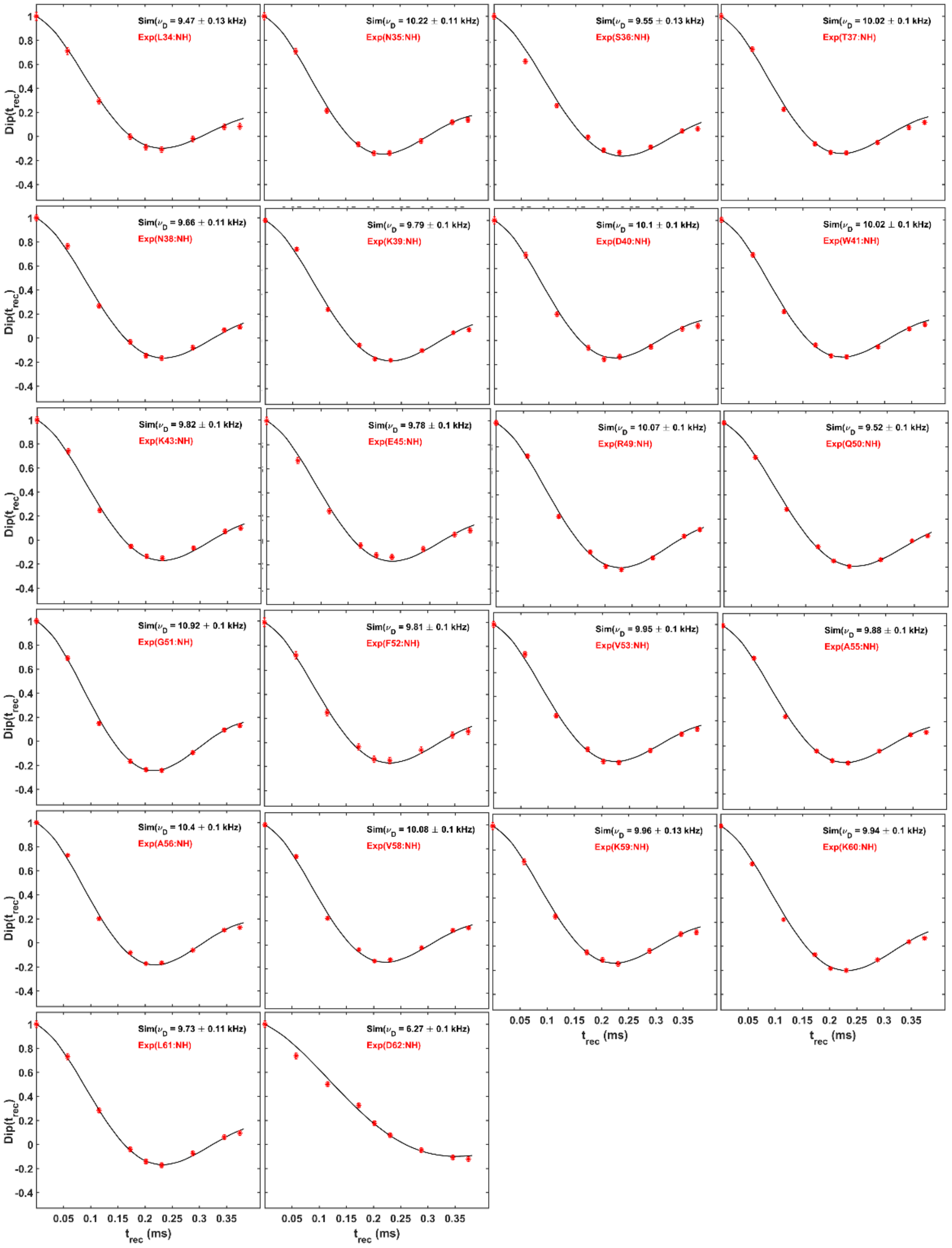

Figure S16 Experimental SH3 (with continuous CP condition of 270°(0.75ν_R)/ 630°(1.75ν_R)) and simulated **Dip curves for HN dipolar coupling values**. The data was acquired on a 600 MHz spectrometer. The simulated pMODERN curves were obtained using the $\Delta\alpha_{rf,max}$ values (summarized in Figure S10B, black stars) and $T_{2,eff}$ = 0.468 ms for better fitting. The fitting errors were obtained by generating 200 Monte Carlo curves, assuming a Gaussian noise distribution (2σ).

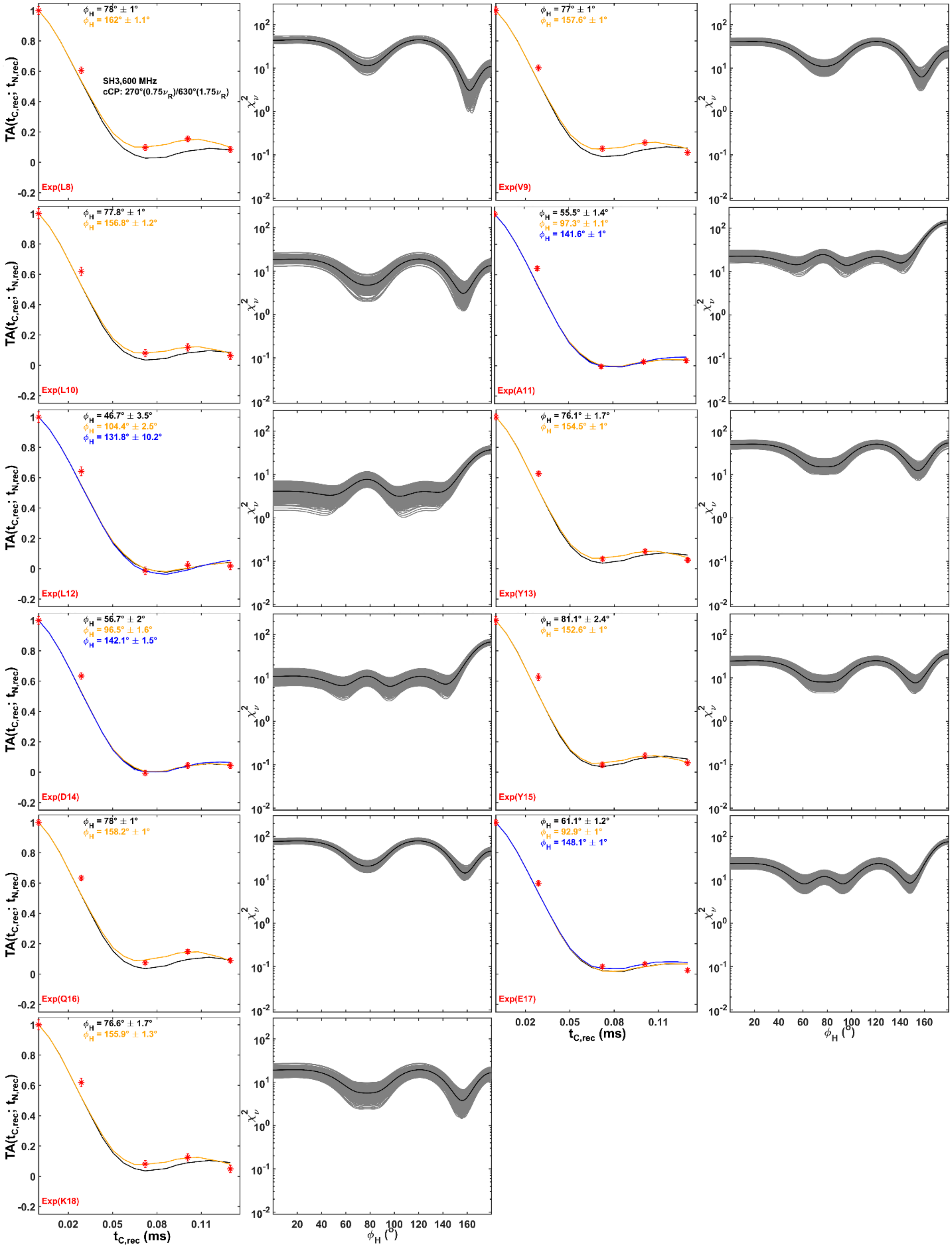

SH3,600 MHz
cCP: 270°(0.75νR)/630°(1.75νR)
ϕH = 78° ± 1°
ϕH = 162° ± 1.1°
Exp(L8)
ϕH = 77° ± 1°
ϕH = 157.6° ± 1°
Exp(V9)
ϕH = 77.8° ± 1°
ϕH = 156.8° ± 1.2°
Exp(L10)
ϕH = 55.5° ± 1.4°
ϕH = 97.3° ± 1.1°
ϕH = 141.6° ± 1°
Exp(A11)
ϕH = 46.7° ± 3.5°
ϕH = 104.4° ± 2.5°
ϕH = 131.8° ± 10.2°
Exp(L12)
ϕH = 76.1° ± 1.7°
ϕH = 154.5° ± 1°
Exp(Y13)
ϕH = 56.7° ± 2°
ϕH = 96.5° ± 1.6°
ϕH = 142.1° ± 1.5°
Exp(D14)
ϕH = 81.1° ± 2.4°
ϕH = 152.6° ± 1°
Exp(Y15)
ϕH = 78° ± 1°
ϕH = 158.2° ± 1°
Exp(Q16)
ϕH = 61.1° ± 1.2°
ϕH = 92.9° ± 1°
ϕH = 148.1° ± 1°
Exp(E17)
ϕH = 76.6° ± 1.7°
ϕH = 155.9° ± 1.3°
Exp(K18)
TA(tC,rec; tN,rec)
χ²ν
tC,rec (ms)
ϕH (°)

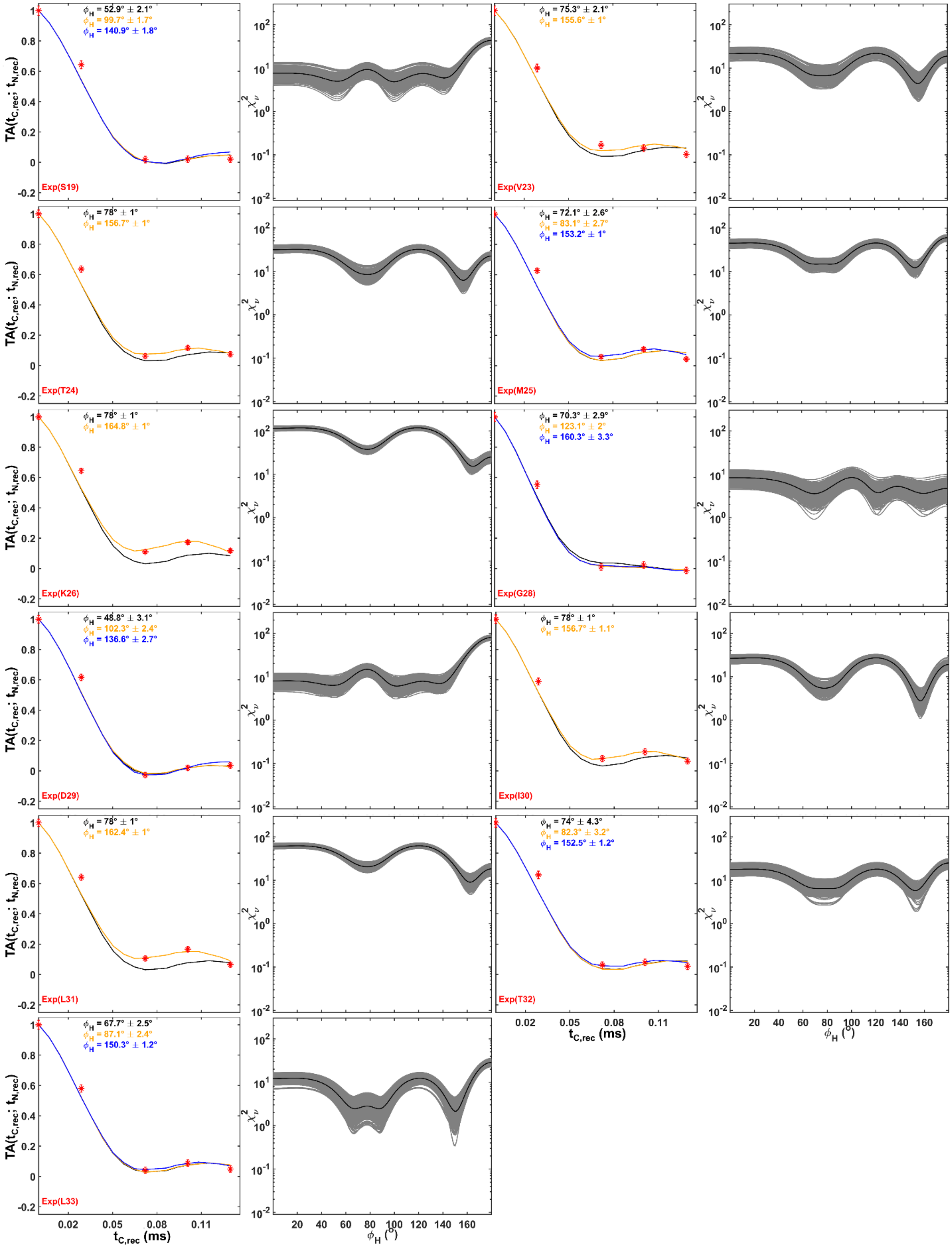

φH = 52.9° ± 2.1°
φH = 99.7° ± 1.7°
φH = 140.9° ± 1.8°
Exp(S19)
φH = 75.3° ± 2.1°
φH = 155.6° ± 1°
Exp(V23)
φH = 78° ± 1°
φH = 156.7° ± 1°
Exp(T24)
φH = 72.1° ± 2.6°
φH = 83.1° ± 2.7°
φH = 153.2° ± 1°
Exp(M25)
φH = 78° ± 1°
φH = 164.8° ± 1°
Exp(K26)
φH = 70.3° ± 2.9°
φH = 123.1° ± 2°
φH = 160.3° ± 3.3°
Exp(G28)
φH = 48.8° ± 3.1°
φH = 102.3° ± 2.4°
φH = 136.6° ± 2.7°
Exp(D29)
φH = 78° ± 1°
φH = 156.7° ± 1.1°
Exp(I30)
φH = 78° ± 1°
φH = 162.4° ± 1°
Exp(L31)
φH = 74° ± 4.3°
φH = 82.3° ± 3.2°
φH = 152.5° ± 1.2°
Exp(T32)
φH = 67.7° ± 2.5°
φH = 87.1° ± 2.4°
φH = 150.3° ± 1.2°
Exp(L33)
TA(tC,rec; tN,rec)
χ²ν
tC,rec (ms)
φH (°)

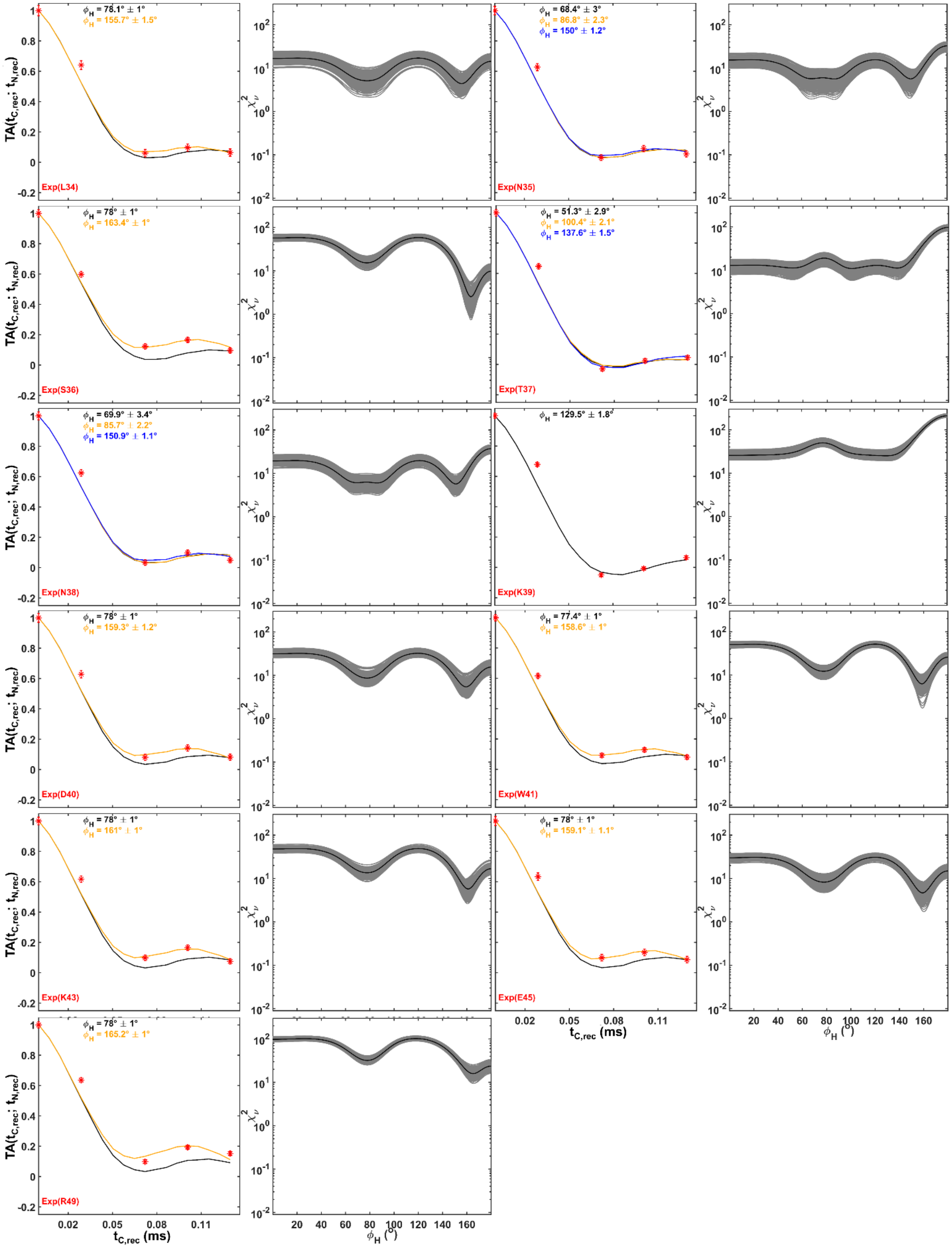
ϕ_H = 78.1° ± 1°
ϕ_H = 155.7° ± 1.5°
Exp(L34)
ϕ_H = 68.4° ± 3°
ϕ_H = 86.8° ± 2.3°
ϕ_H = 150° ± 1.2°
Exp(N35)
ϕ_H = 78° ± 1°
ϕ_H = 163.4° ± 1°
Exp(S36)
ϕ_H = 51.3° ± 2.9°
ϕ_H = 100.4° ± 2.1°
ϕ_H = 137.6° ± 1.5°
Exp(T37)
ϕ_H = 69.9° ± 3.4°
ϕ_H = 85.7° ± 2.2°
ϕ_H = 150.9° ± 1.1°
Exp(N38)
ϕ_H = 129.5° ± 1.8°
Exp(K39)
ϕ_H = 78° ± 1°
ϕ_H = 159.3° ± 1.2°
Exp(D40)
ϕ_H = 77.4° ± 1°
ϕ_H = 158.6° ± 1°
Exp(W41)
ϕ_H = 78° ± 1°
ϕ_H = 161° ± 1°
Exp(K43)
ϕ_H = 78° ± 1°
ϕ_H = 159.1° ± 1.1°
Exp(E45)
ϕ_H = 78° ± 1°
ϕ_H = 165.2° ± 1°
Exp(R49)
TA($t_{C,rec}$; $t_{N,rec}$)
χ^2_ν
$t_{C,rec}$ (ms)
ϕ_H (°)

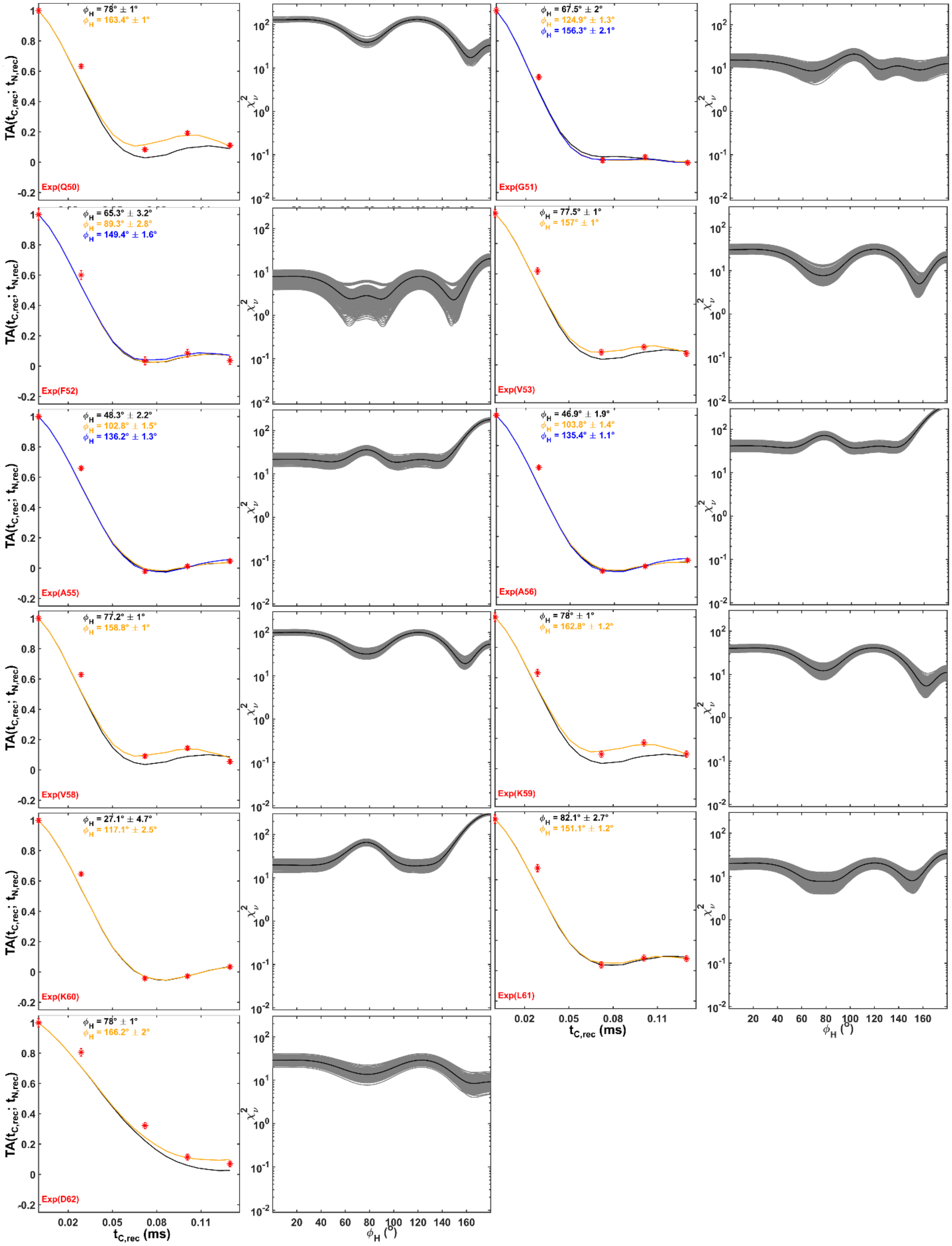

ϕH = 78° ± 1°
ϕH = 163.4° ± 1°
Exp(Q50)
ϕH = 67.5° ± 2°
ϕH = 124.9° ± 1.3°
ϕH = 156.3° ± 2.1°
Exp(G51)
ϕH = 65.3° ± 3.2°
ϕH = 89.3° ± 2.8°
ϕH = 149.4° ± 1.6°
Exp(F52)
ϕH = 77.5° ± 1°
ϕH = 157° ± 1°
Exp(V53)
ϕH = 48.3° ± 2.2°
ϕH = 102.8° ± 1.5°
ϕH = 136.2° ± 1.3°
Exp(A55)
ϕH = 46.9° ± 1.9°
ϕH = 103.8° ± 1.4°
ϕH = 135.4° ± 1.1°
Exp(A56)
ϕH = 77.2° ± 1°
ϕH = 158.8° ± 1°
Exp(V58)
ϕH = 78° ± 1°
ϕH = 162.8° ± 1.2°
Exp(K59)
ϕH = 27.1° ± 4.7°
ϕH = 117.1° ± 2.5°
Exp(K60)
ϕH = 82.1° ± 2.7°
ϕH = 151.1° ± 1.2°
Exp(L61)
ϕH = 78° ± 1°
ϕH = 166.2° ± 2°
Exp(D62)
TA(tC,rec; tN,rec)
χ²ν
tC,rec (ms)
ϕH (°)

Figure S17 Experimental SH3 (with continuous CP condition of 270°(0.75ν_R)/ 630°(1.75ν_R)) and simulated **TA curves for torsion angle values**. The data was acquired on a 600 MHz spectrometer. The simulated pMODERN curves were obtained using the $\Delta\alpha_{rf,max}$ values (summarized in Figures S10A and S10B, black stars) and $T_{2,eff} = 0.468$ ms for better fitting. The fitting errors were obtained by generating 200 Monte Carlo curves, assuming a Gaussian noise distribution (2σ).

Figures 18, 19 and 20 show the experimental and simulated Dip CH, Dip NH and TA curves for CP condition with windowed shape: 270°(1.5ν_R)/ 630°(1.75ν_R).

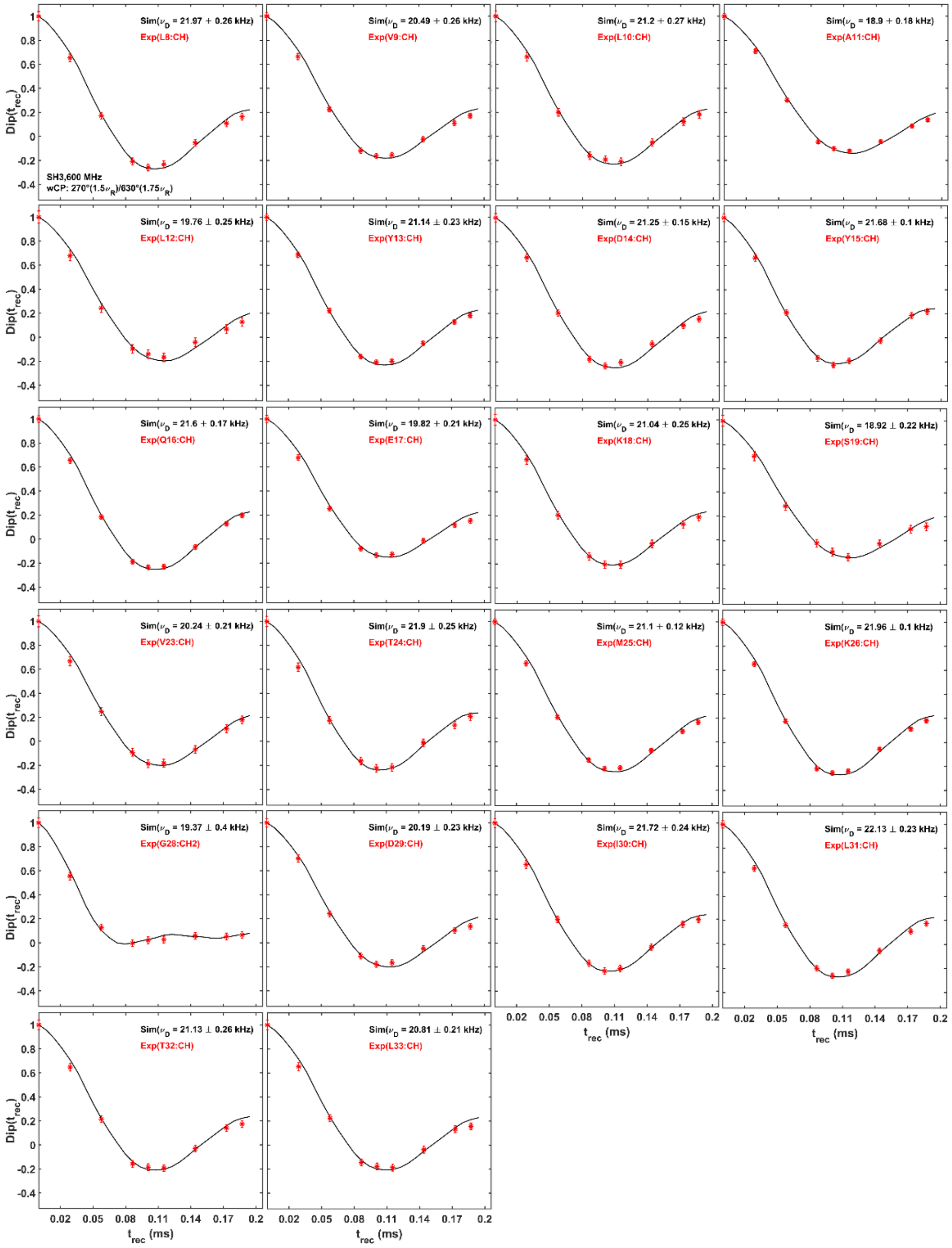
Sim(ν_D = 21.97 ± 0.26 kHz)
Exp(L8:CH)
SH3,600 MHz
wCP: 270°(1.5ν_R)/630°(1.75ν_R)
Sim(ν_D = 20.49 ± 0.26 kHz)
Exp(V9:CH)
Sim(ν_D = 21.2 ± 0.27 kHz)
Exp(L10:CH)
Sim(ν_D = 18.9 ± 0.18 kHz)
Exp(A11:CH)
Sim(ν_D = 19.76 ± 0.25 kHz)
Exp(L12:CH)
Sim(ν_D = 21.14 ± 0.23 kHz)
Exp(Y13:CH)
Sim(ν_D = 21.25 ± 0.15 kHz)
Exp(D14:CH)
Sim(ν_D = 21.68 ± 0.1 kHz)
Exp(Y15:CH)
Sim(ν_D = 21.6 ± 0.17 kHz)
Exp(Q16:CH)
Sim(ν_D = 19.82 ± 0.21 kHz)
Exp(E17:CH)
Sim(ν_D = 21.04 ± 0.25 kHz)
Exp(K18:CH)
Sim(ν_D = 18.92 ± 0.22 kHz)
Exp(S19:CH)
Sim(ν_D = 20.24 ± 0.21 kHz)
Exp(V23:CH)
Sim(ν_D = 21.9 ± 0.25 kHz)
Exp(T24:CH)
Sim(ν_D = 21.1 ± 0.12 kHz)
Exp(M25:CH)
Sim(ν_D = 21.96 ± 0.1 kHz)
Exp(K26:CH)
Sim(ν_D = 19.37 ± 0.4 kHz)
Exp(G28:CH2)
Sim(ν_D = 20.19 ± 0.23 kHz)
Exp(D29:CH)
Sim(ν_D = 21.72 ± 0.24 kHz)
Exp(I30:CH)
Sim(ν_D = 22.13 ± 0.23 kHz)
Exp(L31:CH)
Sim(ν_D = 21.13 ± 0.26 kHz)
Exp(T32:CH)
Sim(ν_D = 20.81 ± 0.21 kHz)
Exp(L33:CH)
Dip(t_rec)
1
0.8
0.6
0.4
0.2
0
-0.2
-0.4
0.02
0.05
0.08
0.11
0.14
0.17
0.2
t_rec (ms)

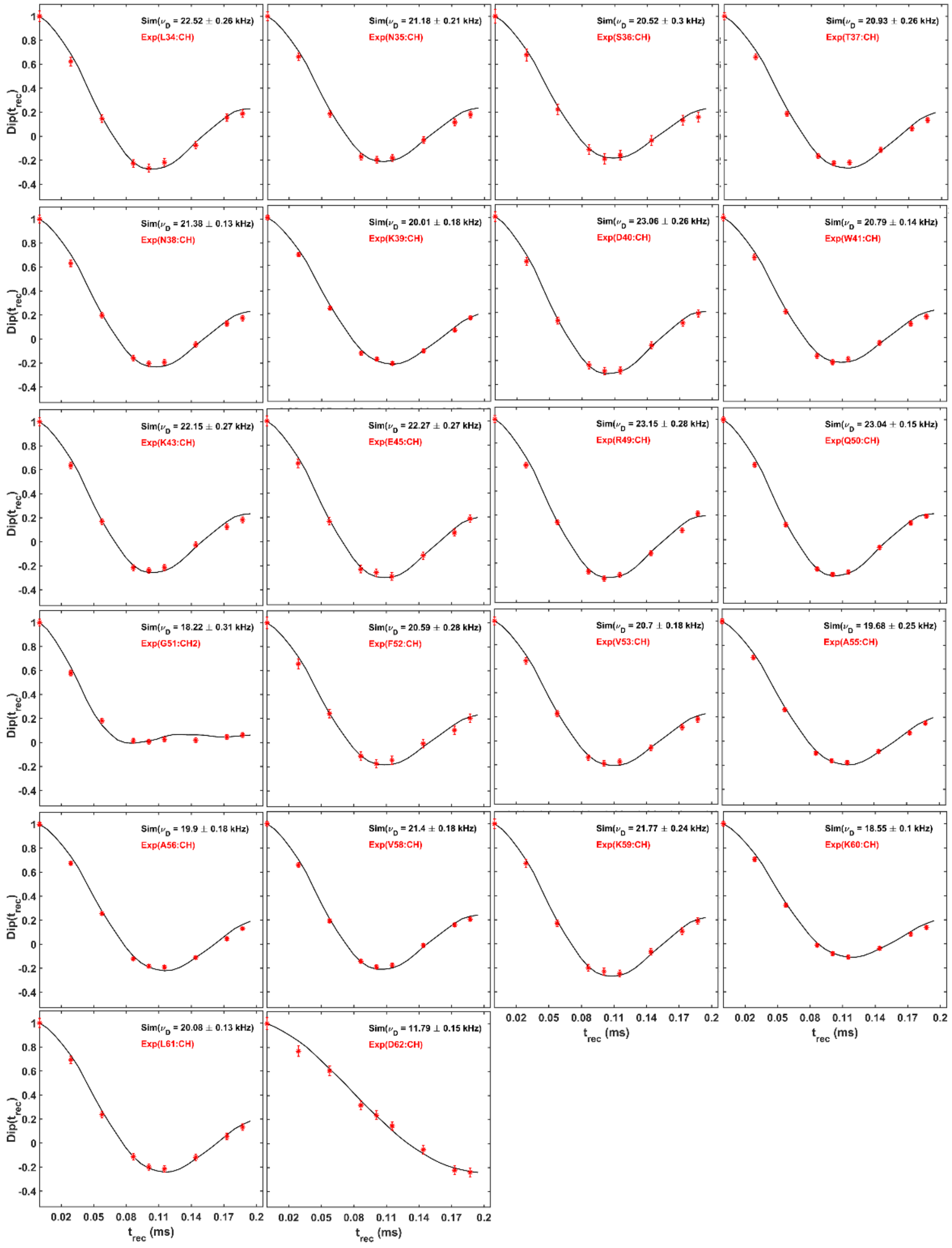

Figure S18 Experimental SH3 (with windowed CP condition of 270°(1.5ν_R)/ 630°(1.75ν_R)) and simulated **Dip curves for HC dipolar coupling values**. The data was acquired on a 600 MHz spectrometer. The simulated pMODERN curves were obtained using the $\Delta\alpha_{rf,max}$ values (summarized in Figure S10A, blue diamonds) and $T_{2,eff} = 0.353$ ms for better fitting. The fitting errors were obtained by generating 200 Monte Carlo curves, assuming a Gaussian noise distribution (2σ).

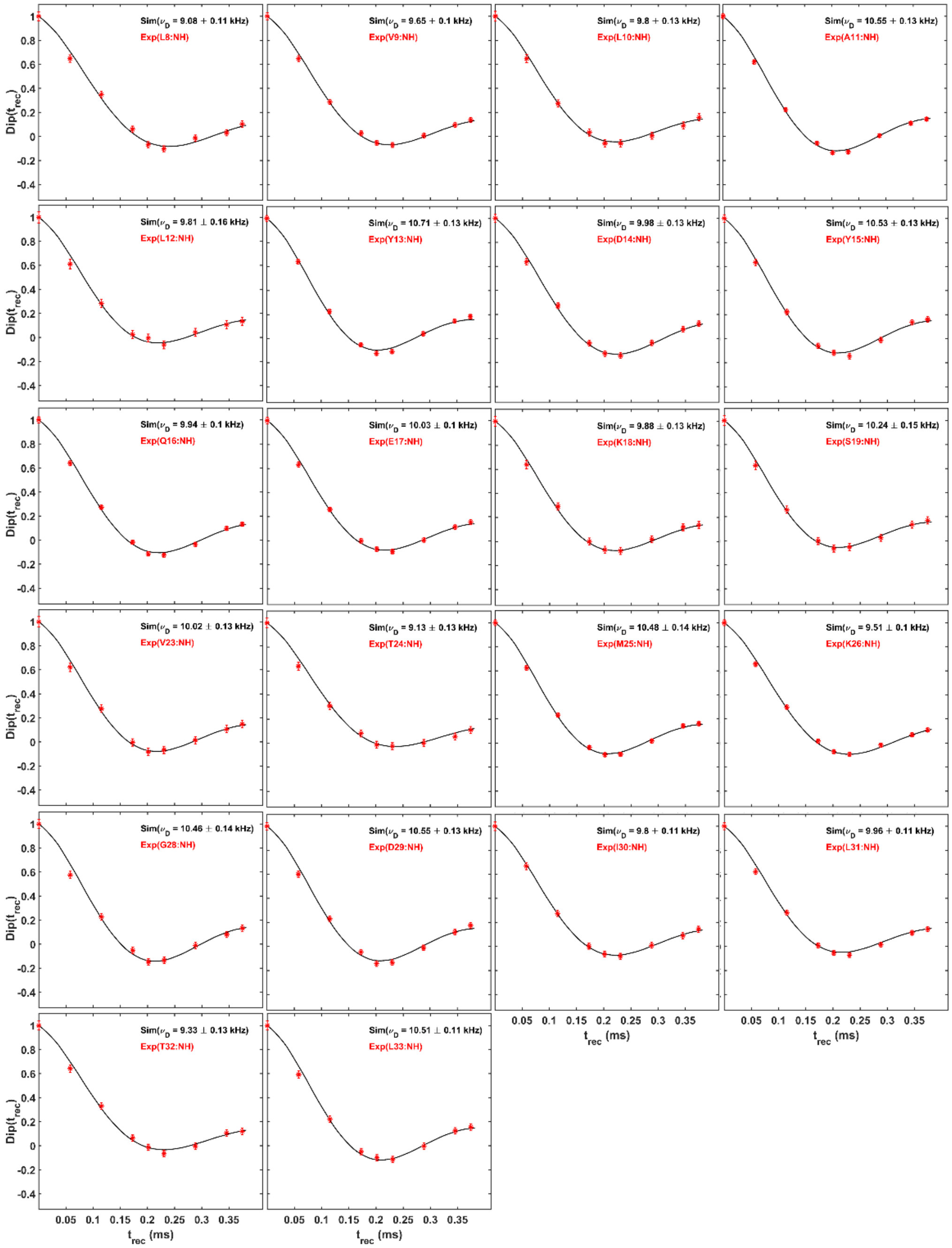

Sim(νD = 9.08 ± 0.11 kHz)
Exp(L8:NH)
Sim(νD = 9.65 ± 0.1 kHz)
Exp(V9:NH)
Sim(νD = 9.8 ± 0.13 kHz)
Exp(L10:NH)
Sim(νD = 10.55 ± 0.13 kHz)
Exp(A11:NH)
Sim(νD = 9.81 ± 0.16 kHz)
Exp(L12:NH)
Sim(νD = 10.71 ± 0.13 kHz)
Exp(Y13:NH)
Sim(νD = 9.98 ± 0.13 kHz)
Exp(D14:NH)
Sim(νD = 10.53 ± 0.13 kHz)
Exp(Y15:NH)
Sim(νD = 9.94 ± 0.1 kHz)
Exp(Q16:NH)
Sim(νD = 10.03 ± 0.1 kHz)
Exp(E17:NH)
Sim(νD = 9.88 ± 0.13 kHz)
Exp(K18:NH)
Sim(νD = 10.24 ± 0.15 kHz)
Exp(S19:NH)
Sim(νD = 10.02 ± 0.13 kHz)
Exp(V23:NH)
Sim(νD = 9.13 ± 0.13 kHz)
Exp(T24:NH)
Sim(νD = 10.48 ± 0.14 kHz)
Exp(M25:NH)
Sim(νD = 9.51 ± 0.1 kHz)
Exp(K26:NH)
Sim(νD = 10.46 ± 0.14 kHz)
Exp(G28:NH)
Sim(νD = 10.55 ± 0.13 kHz)
Exp(D29:NH)
Sim(νD = 9.8 ± 0.11 kHz)
Exp(I30:NH)
Sim(νD = 9.96 ± 0.11 kHz)
Exp(L31:NH)
Sim(νD = 9.33 ± 0.13 kHz)
Exp(T32:NH)
Sim(νD = 10.51 ± 0.11 kHz)
Exp(L33:NH)
Dip(trec)
trec (ms)

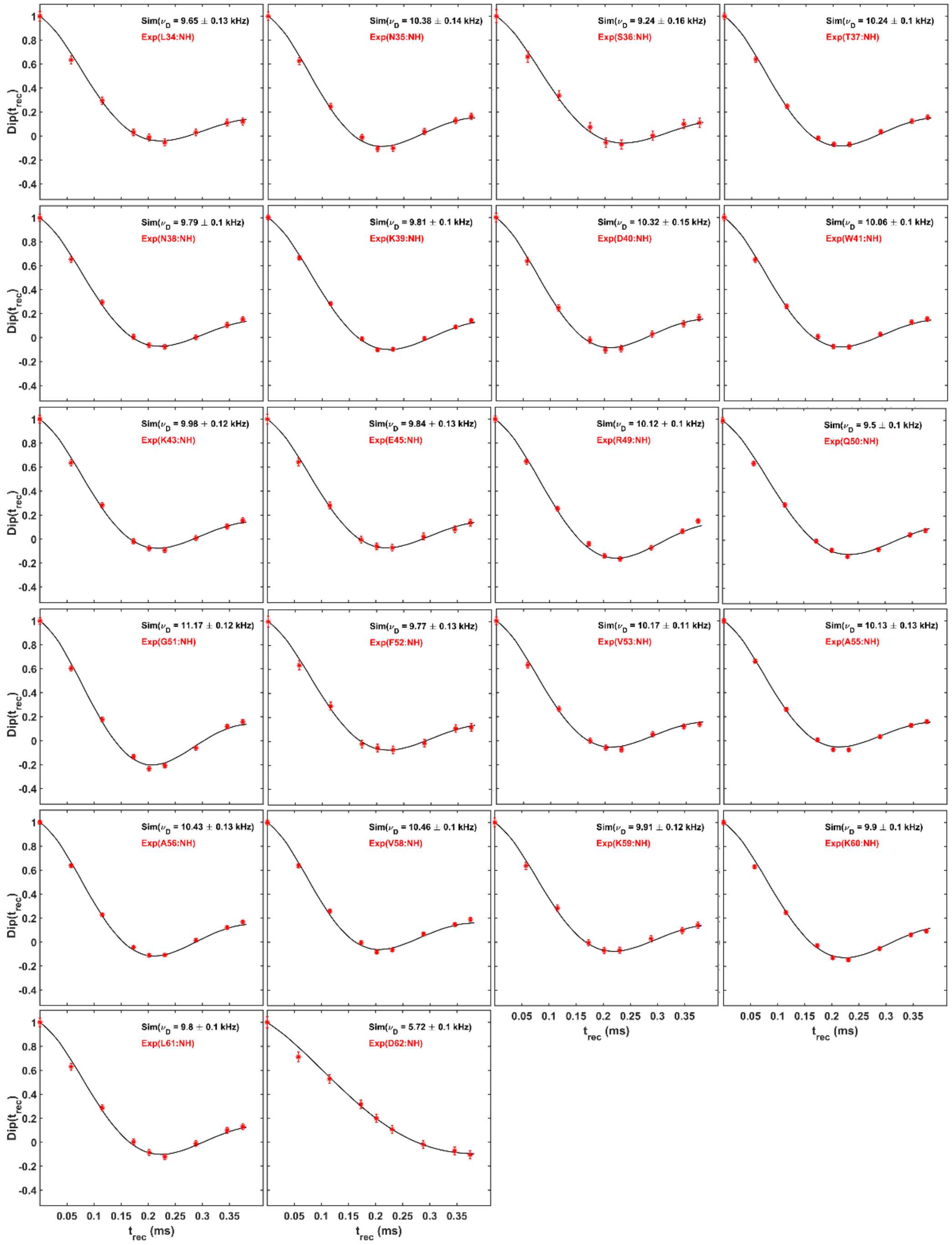

Figure S19 Experimental SH3 (with windowed CP condition of 270°($1.5\nu_R$)/ 630°($1.75\nu_R$)) and simulated **Dip curves for HN dipolar coupling values**. The data was acquired on a 600 MHz spectrometer. The simulated pMODERN curves were obtained using the $\Delta\alpha_{rf,max}$ values (summarized in Figure S10B, blue diamonds) and $T_{2,eff} = 0.353$ ms for better fitting. The fitting errors were obtained by generating 200 Monte Carlo curves, assuming a Gaussian noise distribution (2σ).

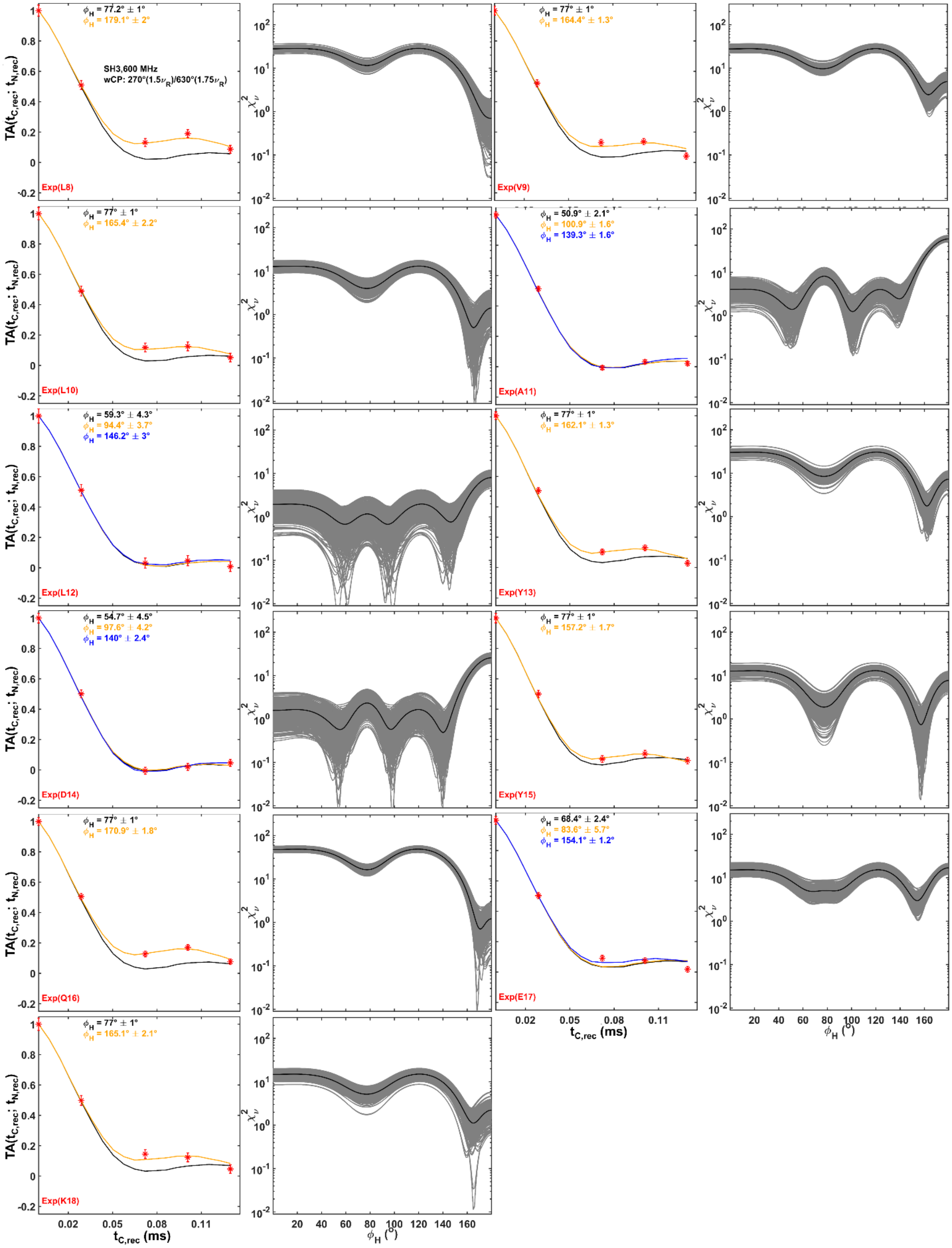
ϕ_H = 77.2° ± 1°
ϕ_H = 179.1° ± 2°
SH3,600 MHz
wCP: 270°(1.5ν_R)/630°(1.75ν_R)
TA($t_{C,rec}$; $t_{N,rec}$)
Exp(L8)
ϕ_H = 77° ± 1°
ϕ_H = 164.4° ± 1.3°
Exp(V9)
ϕ_H = 77° ± 1°
ϕ_H = 165.4° ± 2.2°
Exp(L10)
ϕ_H = 50.9° ± 2.1°
ϕ_H = 100.9° ± 1.6°
ϕ_H = 139.3° ± 1.6°
Exp(A11)
ϕ_H = 59.3° ± 4.3°
ϕ_H = 94.4° ± 3.7°
ϕ_H = 146.2° ± 3°
Exp(L12)
ϕ_H = 77° ± 1°
ϕ_H = 162.1° ± 1.3°
Exp(Y13)
ϕ_H = 54.7° ± 4.5°
ϕ_H = 97.6° ± 4.2°
ϕ_H = 140° ± 2.4°
Exp(D14)
ϕ_H = 77° ± 1°
ϕ_H = 157.2° ± 1.7°
Exp(Y15)
ϕ_H = 77° ± 1°
ϕ_H = 170.9° ± 1.8°
Exp(Q16)
ϕ_H = 68.4° ± 2.4°
ϕ_H = 83.6° ± 5.7°
ϕ_H = 154.1° ± 1.2°
Exp(E17)
ϕ_H = 77° ± 1°
ϕ_H = 165.1° ± 2.1°
Exp(K18)
χ^2_ν
$t_{C,rec}$ (ms)
ϕ_H (°)

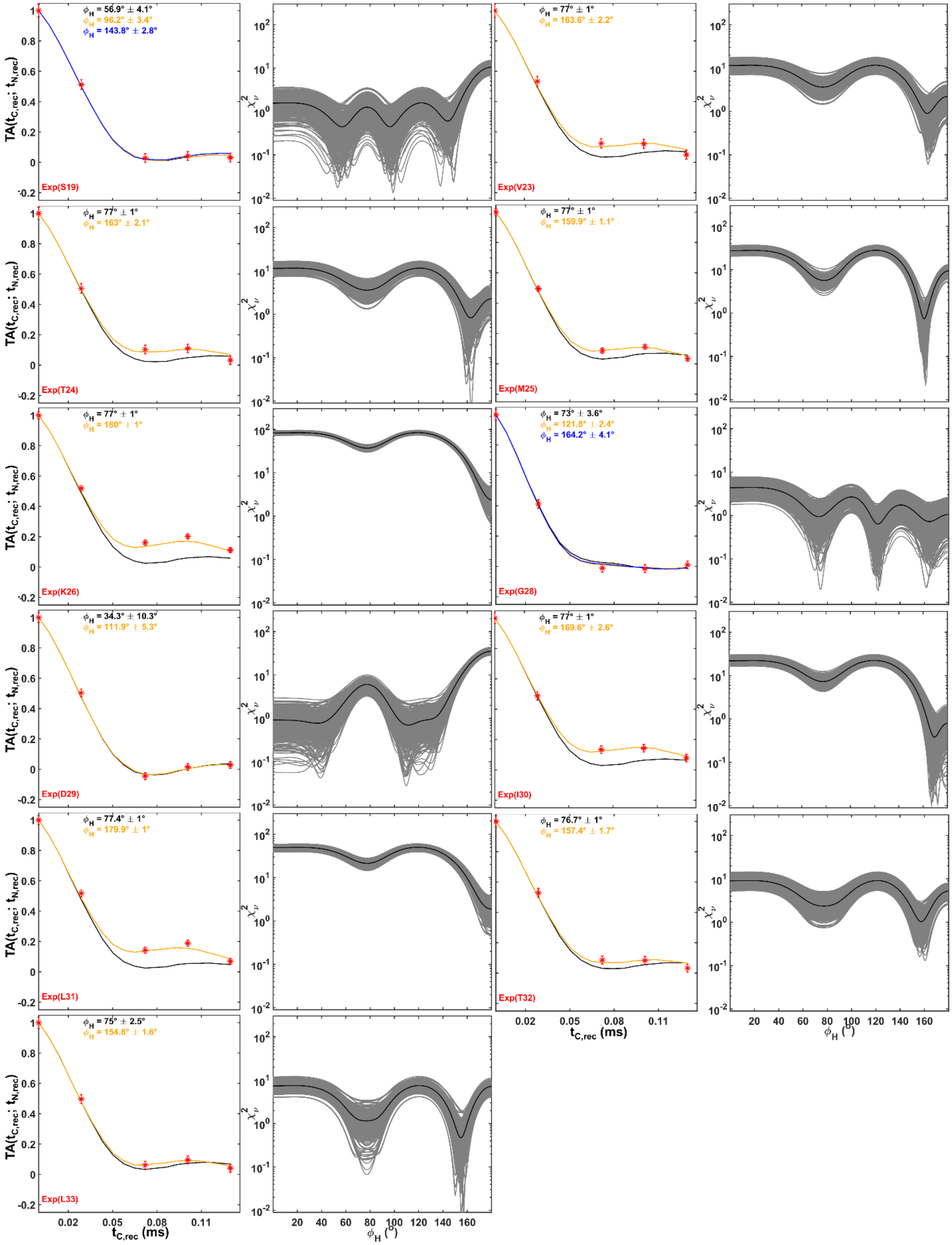

ϕH = 56.9° ± 4.1°
ϕH = 96.2° ± 3.4°
ϕH = 143.8° ± 2.8°
Exp(S19)
ϕH = 77° ± 1°
ϕH = 163.6° ± 2.2°
Exp(V23)
ϕH = 77° ± 1°
ϕH = 163° ± 2.1°
Exp(T24)
ϕH = 77° ± 1°
ϕH = 159.9° ± 1.1°
Exp(M25)
ϕH = 77° ± 1°
ϕH = 180° ± 1°
Exp(K26)
ϕH = 73° ± 3.6°
ϕH = 121.8° ± 2.4°
ϕH = 164.2° ± 4.1°
Exp(G28)
ϕH = 34.3° ± 10.3°
ϕH = 111.9° ± 5.3°
Exp(D29)
ϕH = 77° ± 1°
ϕH = 169.6° ± 2.6°
Exp(I30)
ϕH = 77.4° ± 1°
ϕH = 179.9° ± 1°
Exp(L31)
ϕH = 76.7° ± 1°
ϕH = 157.4° ± 1.7°
Exp(T32)
ϕH = 75° ± 2.5°
ϕH = 154.8° ± 1.6°
Exp(L33)
TA(tC,rec; tN,rec)
χ²ν
tC,rec (ms)
ϕH (°)

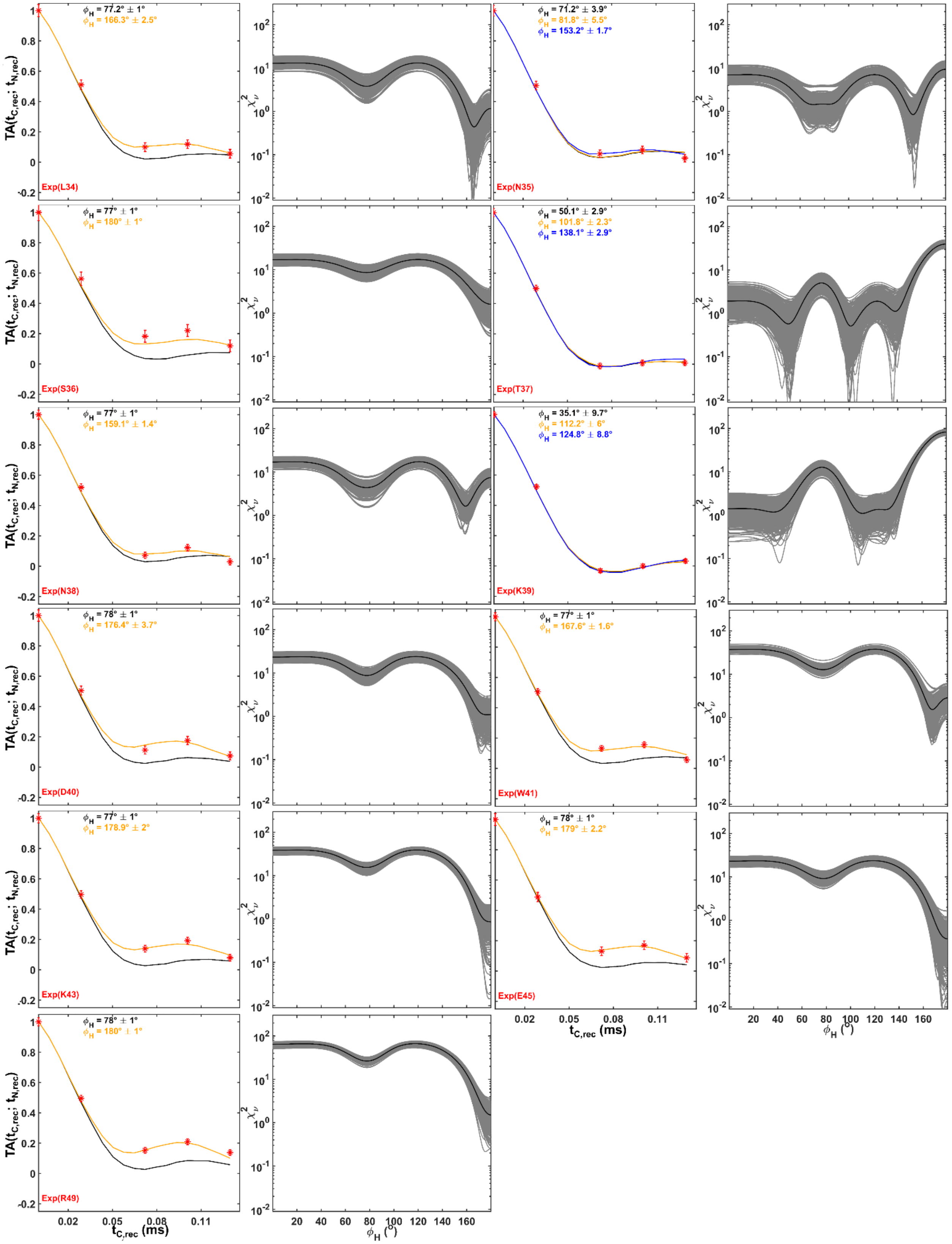

TA(t_C,rec; t_N,rec)
Exp(L34)
φH = 77.2° ± 1°
φH = 166.3° ± 2.5°
Exp(N35)
φH = 71.2° ± 3.9°
φH = 81.8° ± 5.5°
φH = 153.2° ± 1.7°
Exp(S36)
φH = 77° ± 1°
φH = 180° ± 1°
Exp(T37)
φH = 50.1° ± 2.9°
φH = 101.8° ± 2.3°
φH = 138.1° ± 2.9°
Exp(N38)
φH = 77° ± 1°
φH = 159.1° ± 1.4°
Exp(K39)
φH = 35.1° ± 9.7°
φH = 112.2° ± 6°
φH = 124.8° ± 8.8°
Exp(D40)
φH = 78° ± 1°
φH = 176.4° ± 3.7°
Exp(W41)
φH = 77° ± 1°
φH = 167.6° ± 1.6°
Exp(K43)
φH = 77° ± 1°
φH = 178.9° ± 2°
Exp(E45)
φH = 78° ± 1°
φH = 179° ± 2.2°
Exp(R49)
φH = 78° ± 1°
φH = 180° ± 1°
χ²ν
t_C,rec (ms)
φH (°)

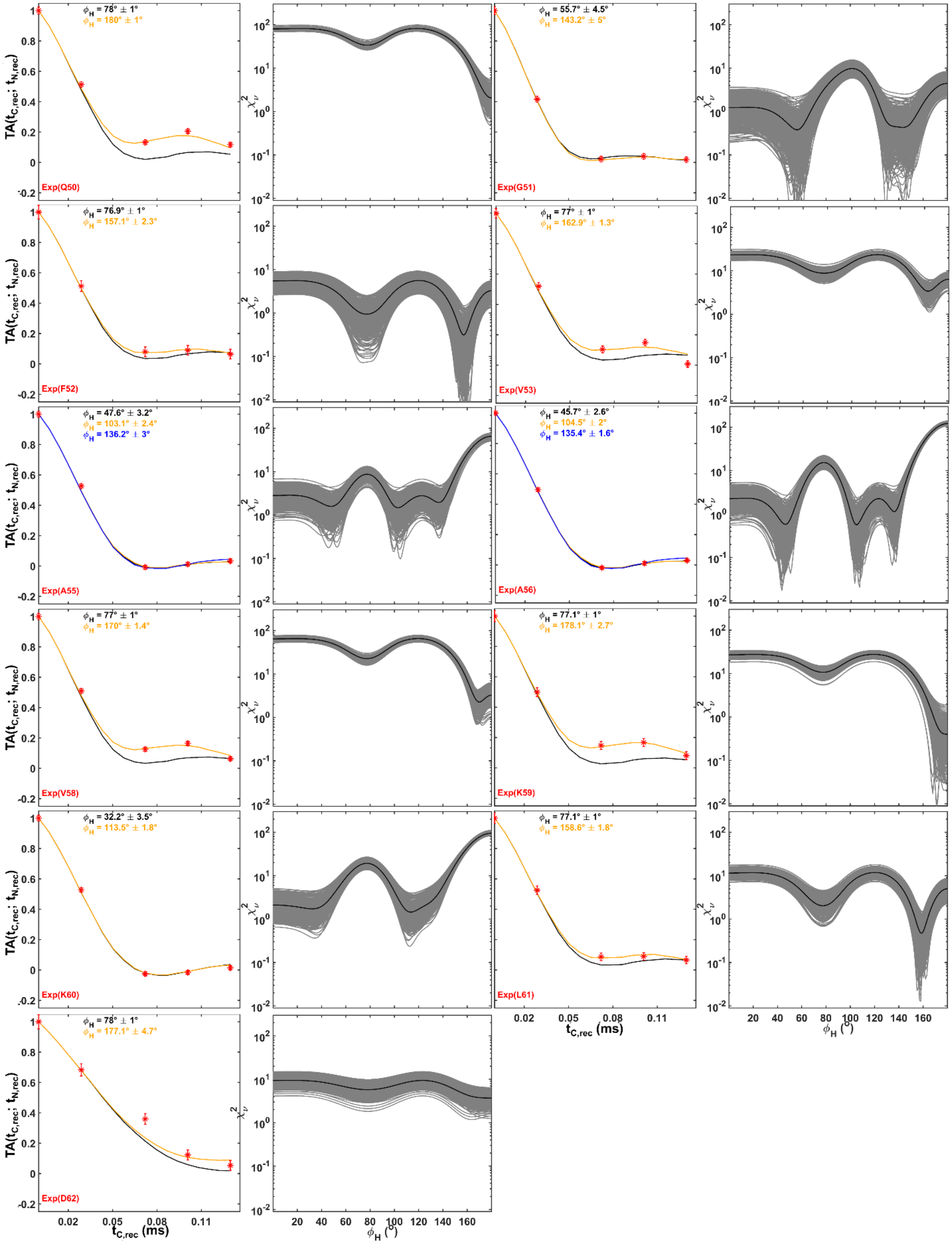
φH = 78° ± 1°
φH = 180° ± 1°
Exp(Q50)
φH = 55.7° ± 4.5°
φH = 143.2° ± 5°
Exp(G51)
φH = 76.9° ± 1°
φH = 157.1° ± 2.3°
Exp(F52)
φH = 77° ± 1°
φH = 162.9° ± 1.3°
Exp(V53)
φH = 47.6° ± 3.2°
φH = 103.1° ± 2.4°
φH = 136.2° ± 3°
Exp(A55)
φH = 45.7° ± 2.6°
φH = 104.5° ± 2°
φH = 135.4° ± 1.6°
Exp(A56)
φH = 77° ± 1°
φH = 170° ± 1.4°
Exp(V58)
φH = 77.1° ± 1°
φH = 178.1° ± 2.7°
Exp(K59)
φH = 32.2° ± 3.5°
φH = 113.5° ± 1.8°
Exp(K60)
φH = 77.1° ± 1°
φH = 158.6° ± 1.8°
Exp(L61)
φH = 78° ± 1°
φH = 177.1° ± 4.7°
Exp(D62)
TA(tC,rec; tN,rec)
χ2ν
tC,rec (ms)
φH (°)

Figure S20 Experimental SH3 (with windowed CP condition of 270°(1.5ν_R)/ 630°(1.75ν_R)) and simulated **TA curves for torsion angle values**. The data was acquired on a 600 MHz spectrometer. The simulated pMODERN curves were obtained using the $\Delta\alpha_{rf,max}$ values (summarized in Figures S10A and S10B, blue diamonds) and $T_{2,eff} = 0.353$ ms for better fitting. The fitting errors were obtained by generating 200 Monte Carlo curves, assuming a Gaussian noise distribution (2σ).

Figures 21, 22 and 23 show the experimental and simulated Dip CH, Dip NH and TA curves for CP condition with windowed shape: 90°(0.5ν_R)/ 450°(2.5ν_R). For TA determination, the last experimental point at 0.1296 ms of $t_{1,rec}$ was excluded from the quantitative analysis of the TA curves and the determination of ϕ_H values.

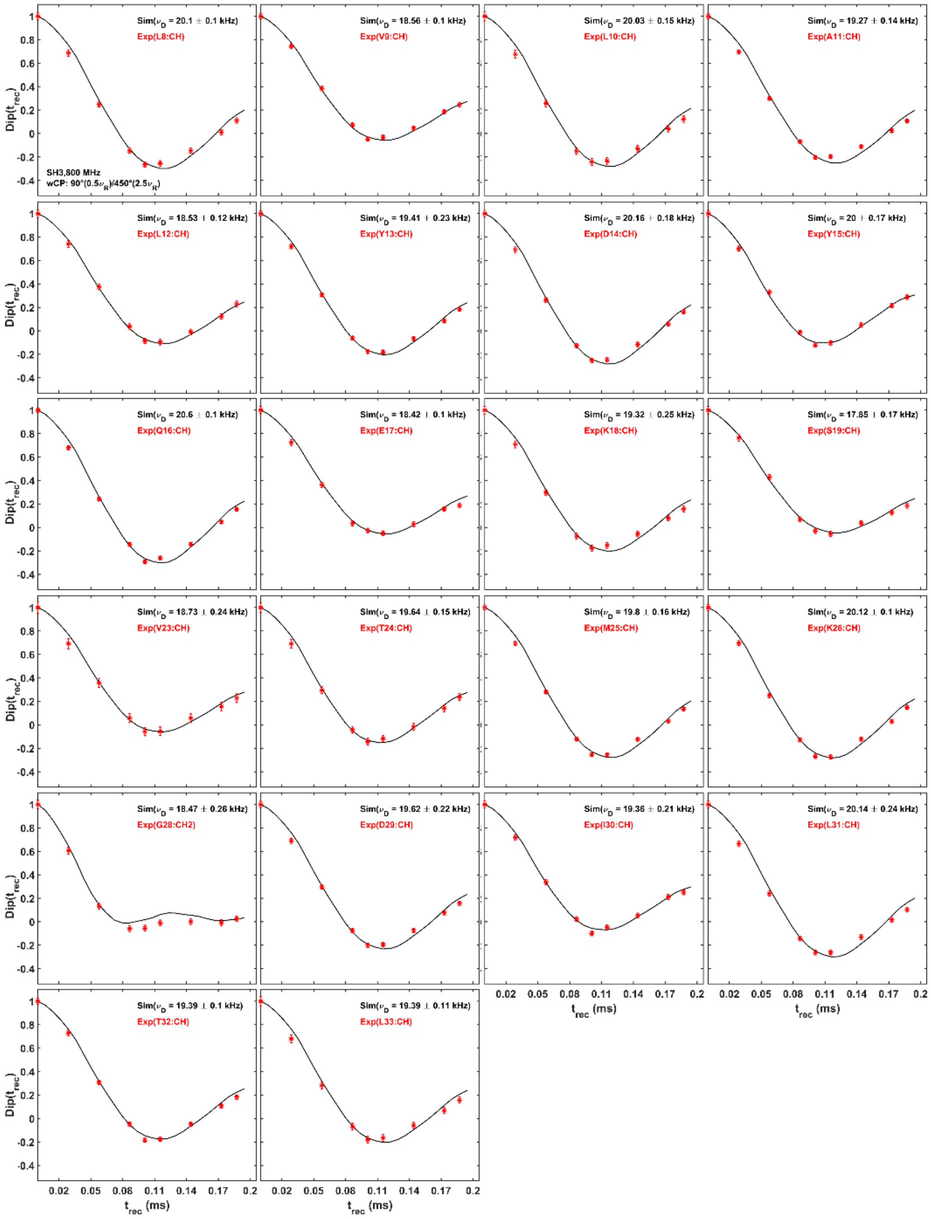
Sim(νD = 20.1 ± 0.1 kHz)
Exp(L8:CH)
SH3,800 MHz
wCP: 90°(0.5νR)/450°(2.5νR)
Sim(νD = 18.56 ± 0.1 kHz)
Exp(V9:CH)
Sim(νD = 20.03 ± 0.15 kHz)
Exp(L10:CH)
Sim(νD = 19.27 ± 0.14 kHz)
Exp(A11:CH)
Sim(νD = 18.53 ± 0.12 kHz)
Exp(L12:CH)
Sim(νD = 19.41 ± 0.23 kHz)
Exp(Y13:CH)
Sim(νD = 20.16 ± 0.18 kHz)
Exp(D14:CH)
Sim(νD = 20 ± 0.17 kHz)
Exp(Y15:CH)
Sim(νD = 20.6 ± 0.1 kHz)
Exp(Q16:CH)
Sim(νD = 18.42 ± 0.1 kHz)
Exp(E17:CH)
Sim(νD = 19.32 ± 0.25 kHz)
Exp(K18:CH)
Sim(νD = 17.85 ± 0.17 kHz)
Exp(S19:CH)
Sim(νD = 18.73 ± 0.24 kHz)
Exp(V23:CH)
Sim(νD = 19.64 ± 0.15 kHz)
Exp(T24:CH)
Sim(νD = 19.8 ± 0.16 kHz)
Exp(M25:CH)
Sim(νD = 20.12 ± 0.1 kHz)
Exp(K26:CH)
Sim(νD = 18.47 ± 0.26 kHz)
Exp(G28:CH2)
Sim(νD = 19.62 ± 0.22 kHz)
Exp(D29:CH)
Sim(νD = 19.36 ± 0.21 kHz)
Exp(I30:CH)
Sim(νD = 20.14 ± 0.24 kHz)
Exp(L31:CH)
Sim(νD = 19.39 ± 0.1 kHz)
Exp(T32:CH)
Sim(νD = 19.39 ± 0.11 kHz)
Exp(L33:CH)
Dip(trec)
trec (ms)

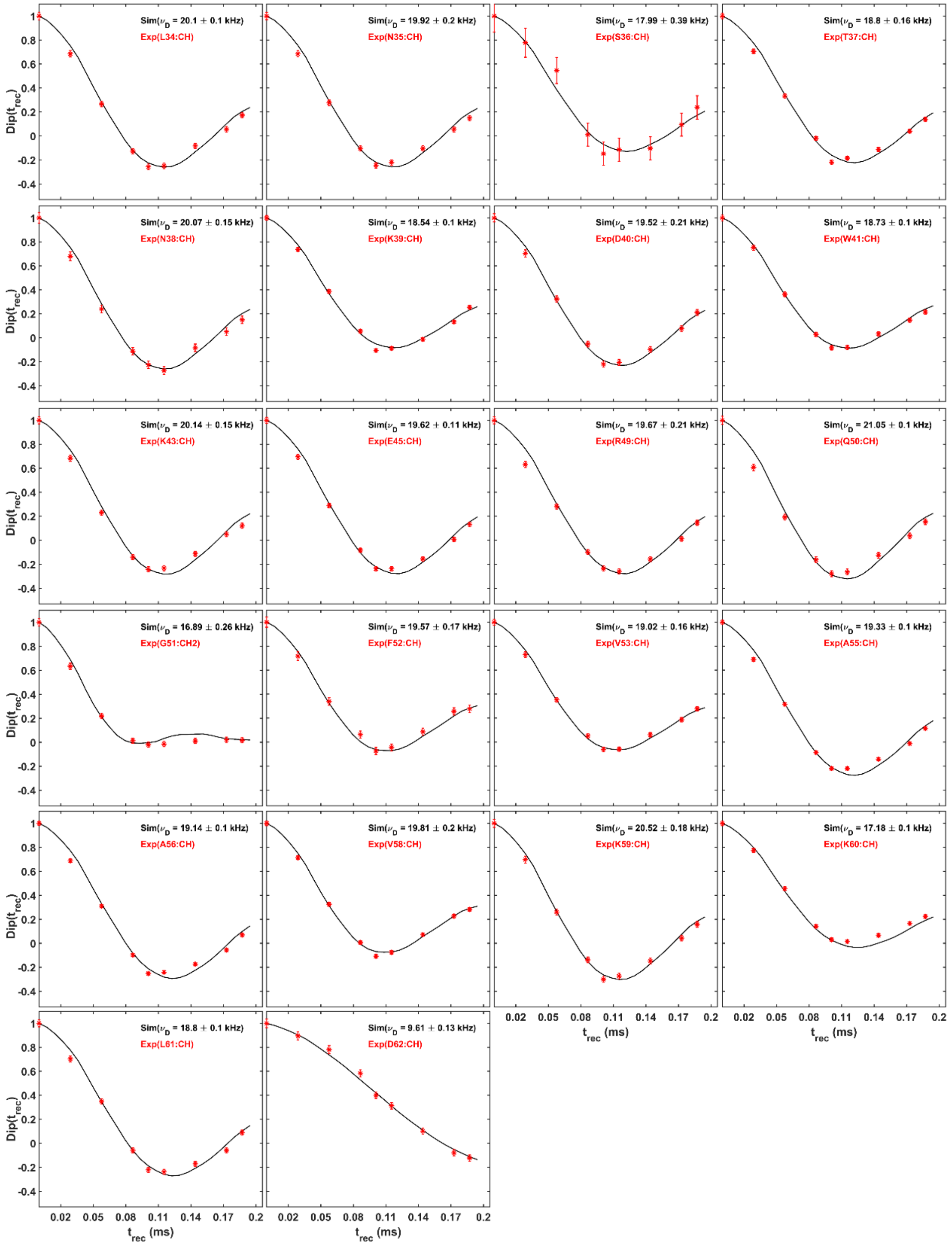

Figure S21 Experimental SH3 (with windowed CP condition of 90°(0.5ν_R)/ 450°(2.5ν_R)) and simulated **Dip curves for HC dipolar coupling values**. The data was acquired on a 800 MHz spectrometer. The simulated pMODERN curves were obtained using the $\Delta\alpha_{rf,max}$ values (summarized in Figure S10A, red circles) and $T_{2,eff} = 0.566$ ms for better fitting. The fitting errors were obtained by generating 200 Monte Carlo curves, assuming a Gaussian noise distribution (2σ).

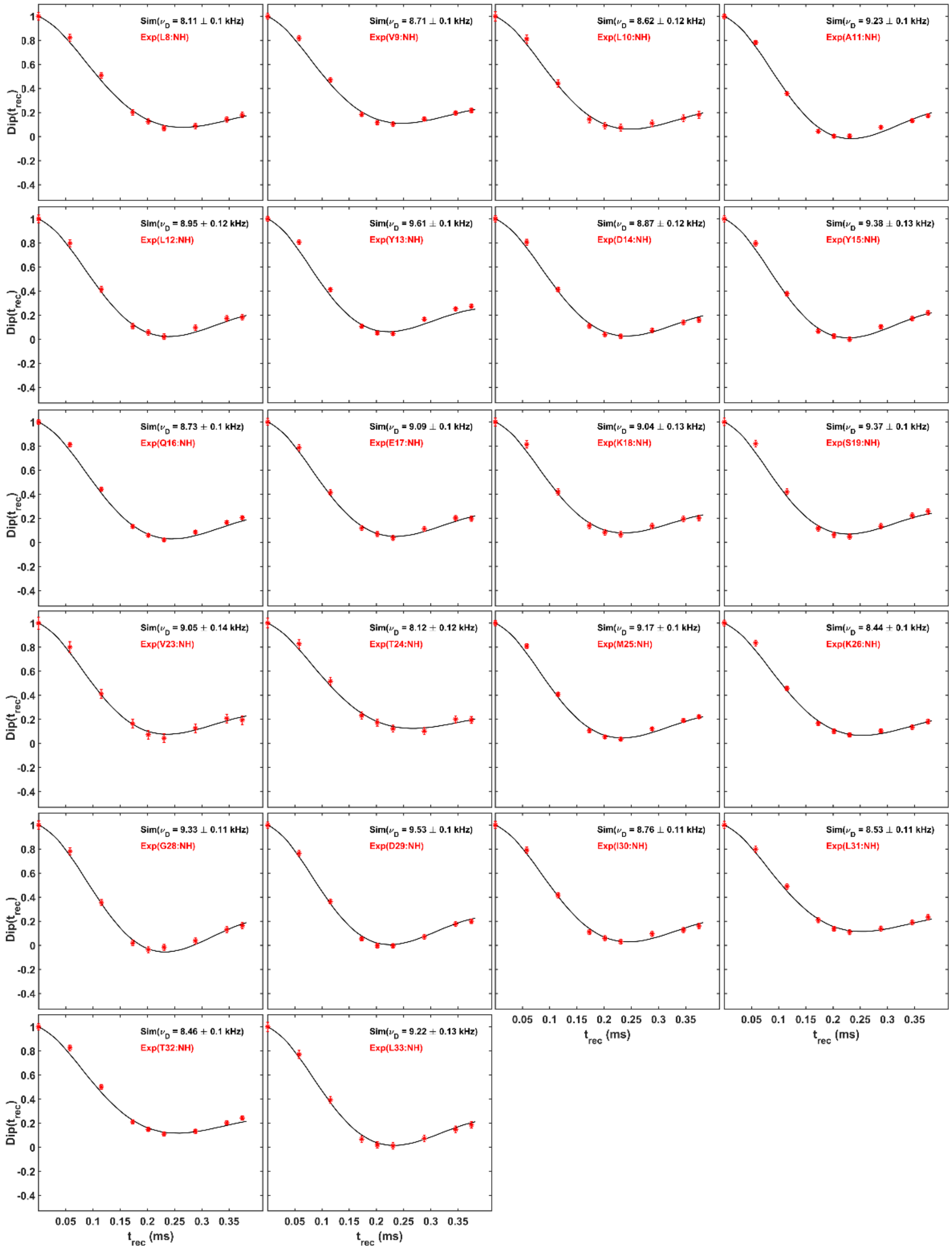

Sim(νD = 8.11 ± 0.1 kHz)
Exp(L8:NH)
Sim(νD = 8.71 ± 0.1 kHz)
Exp(V9:NH)
Sim(νD = 8.62 ± 0.12 kHz)
Exp(L10:NH)
Sim(νD = 9.23 ± 0.1 kHz)
Exp(A11:NH)
Sim(νD = 8.95 ± 0.12 kHz)
Exp(L12:NH)
Sim(νD = 9.61 ± 0.1 kHz)
Exp(Y13:NH)
Sim(νD = 8.87 ± 0.12 kHz)
Exp(D14:NH)
Sim(νD = 9.38 ± 0.13 kHz)
Exp(Y15:NH)
Sim(νD = 8.73 ± 0.1 kHz)
Exp(Q16:NH)
Sim(νD = 9.09 ± 0.1 kHz)
Exp(E17:NH)
Sim(νD = 9.04 ± 0.13 kHz)
Exp(K18:NH)
Sim(νD = 9.37 ± 0.1 kHz)
Exp(S19:NH)
Sim(νD = 9.05 ± 0.14 kHz)
Exp(V23:NH)
Sim(νD = 8.12 ± 0.12 kHz)
Exp(T24:NH)
Sim(νD = 9.17 ± 0.1 kHz)
Exp(M25:NH)
Sim(νD = 8.44 ± 0.1 kHz)
Exp(K26:NH)
Sim(νD = 9.33 ± 0.11 kHz)
Exp(G28:NH)
Sim(νD = 9.53 ± 0.1 kHz)
Exp(D29:NH)
Sim(νD = 8.76 ± 0.11 kHz)
Exp(I30:NH)
Sim(νD = 8.53 ± 0.11 kHz)
Exp(L31:NH)
Sim(νD = 8.46 ± 0.1 kHz)
Exp(T32:NH)
Sim(νD = 9.22 ± 0.13 kHz)
Exp(L33:NH)
Dip(trec)
trec (ms)

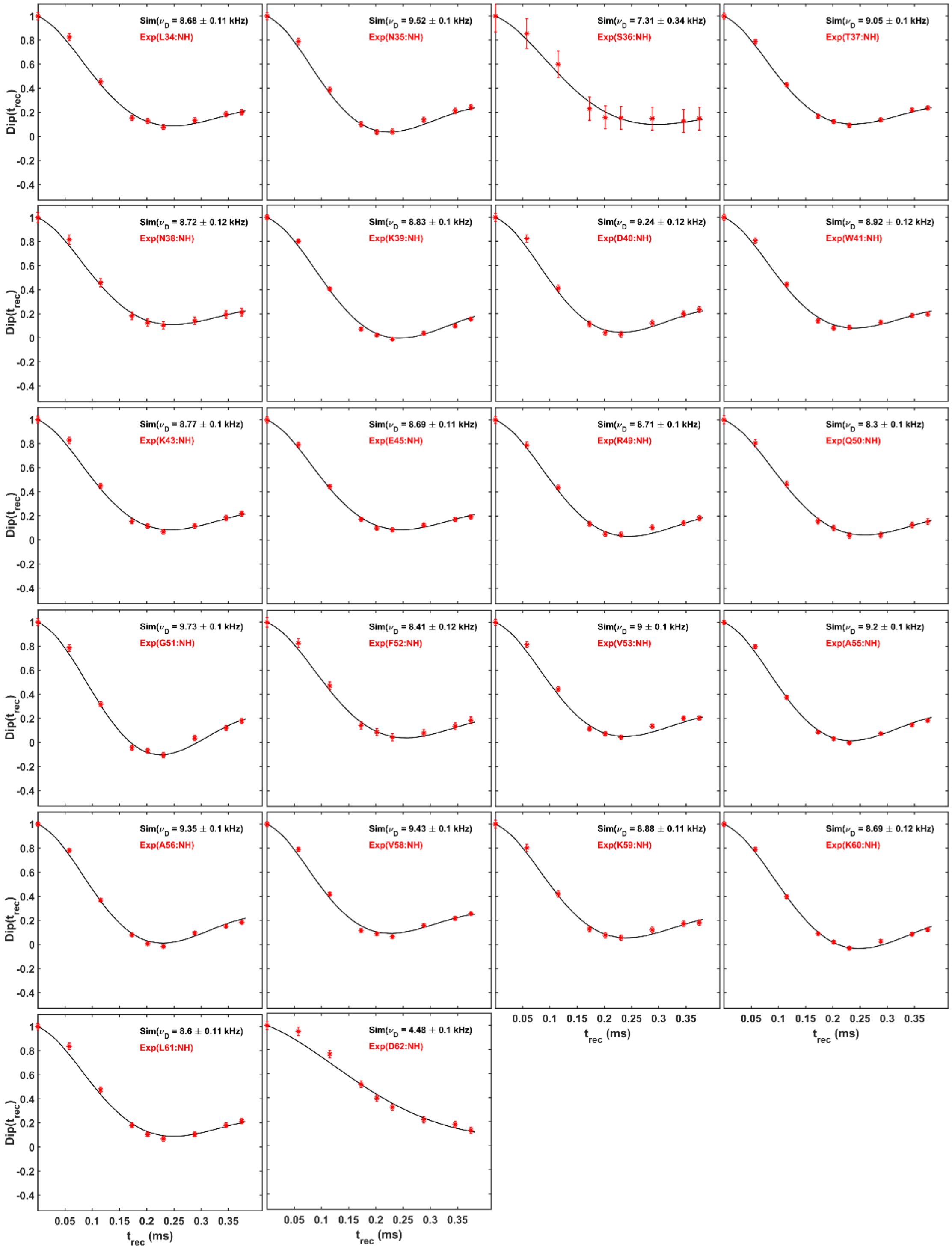

Figure S22 Experimental SH3 (with windowed CP condition of 90°(0.5ν_R)/ 450°(2.5ν_R)) and simulated **Dip curves for HN dipolar coupling values**. The data was acquired on a 800 MHz spectrometer. The simulated pMODERN curves were obtained using the $\Delta\alpha_{rf,max}$ values (summarized in Figure S10B, red circles) and $T_{2,eff}$ = 0.566 ms for better fitting. The fitting errors were obtained by generating 200 Monte Carlo curves, assuming a Gaussian noise distribution (2σ).

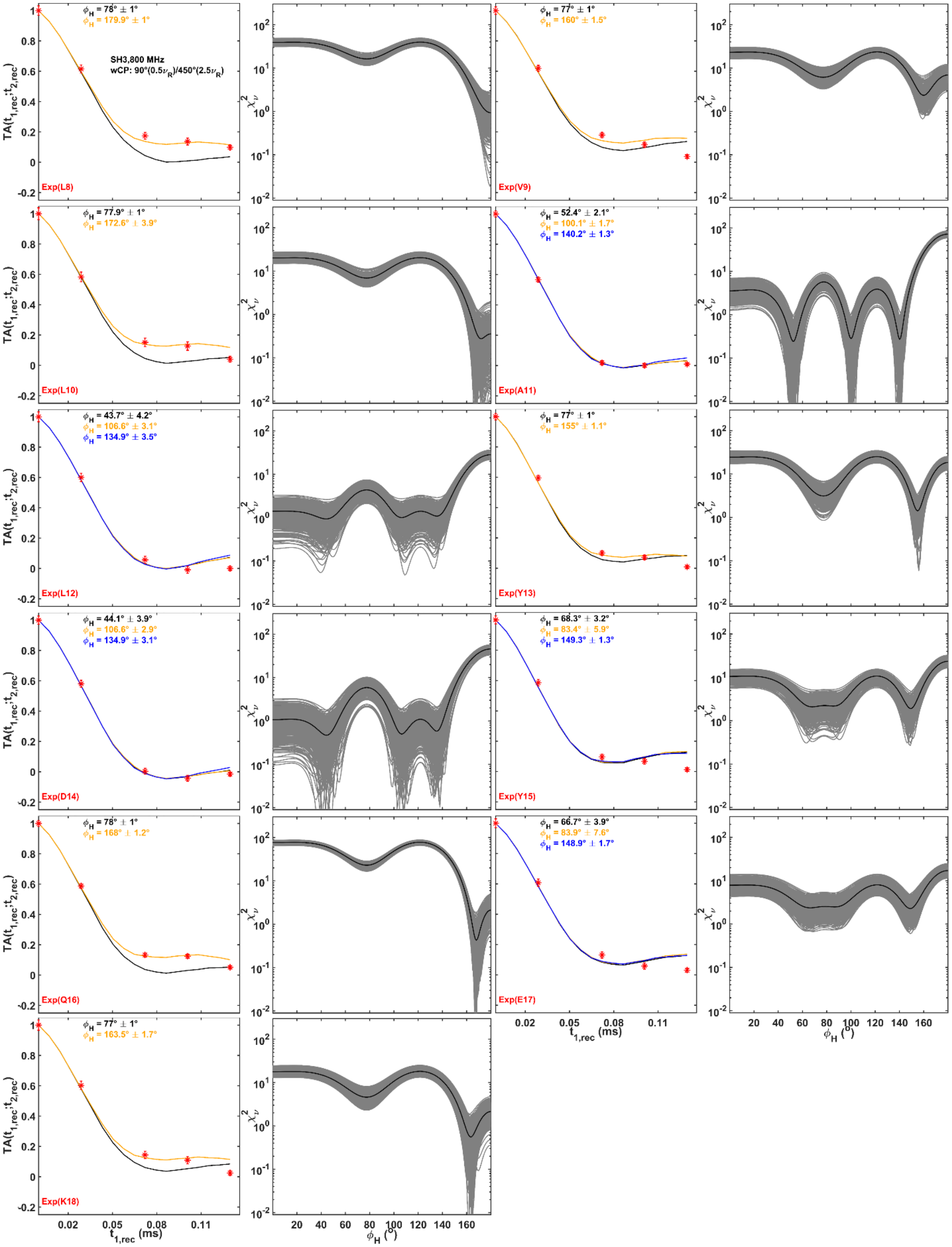

φH = 78° ± 1°
φH = 179.9° ± 1°
SH3,800 MHz
wCP: 90°(0.5νR)/450°(2.5νR)
Exp(L8)
φH = 77° ± 1°
φH = 160° ± 1.5°
Exp(V9)
φH = 77.9° ± 1°
φH = 172.6° ± 3.9°
Exp(L10)
φH = 52.4° ± 2.1°
φH = 100.1° ± 1.7°
φH = 140.2° ± 1.3°
Exp(A11)
φH = 43.7° ± 4.2°
φH = 106.6° ± 3.1°
φH = 134.9° ± 3.5°
Exp(L12)
φH = 77° ± 1°
φH = 155° ± 1.1°
Exp(Y13)
φH = 44.1° ± 3.9°
φH = 106.6° ± 2.9°
φH = 134.9° ± 3.1°
Exp(D14)
φH = 68.3° ± 3.2°
φH = 83.4° ± 5.9°
φH = 149.3° ± 1.3°
Exp(Y15)
φH = 78° ± 1°
φH = 168° ± 1.2°
Exp(Q16)
φH = 66.7° ± 3.9°
φH = 83.9° ± 7.6°
φH = 148.9° ± 1.7°
Exp(E17)
φH = 77° ± 1°
φH = 163.5° ± 1.7°
Exp(K18)
TA(t1,rec;t2,rec)
χ2ν
t1,rec (ms)
φH (°)

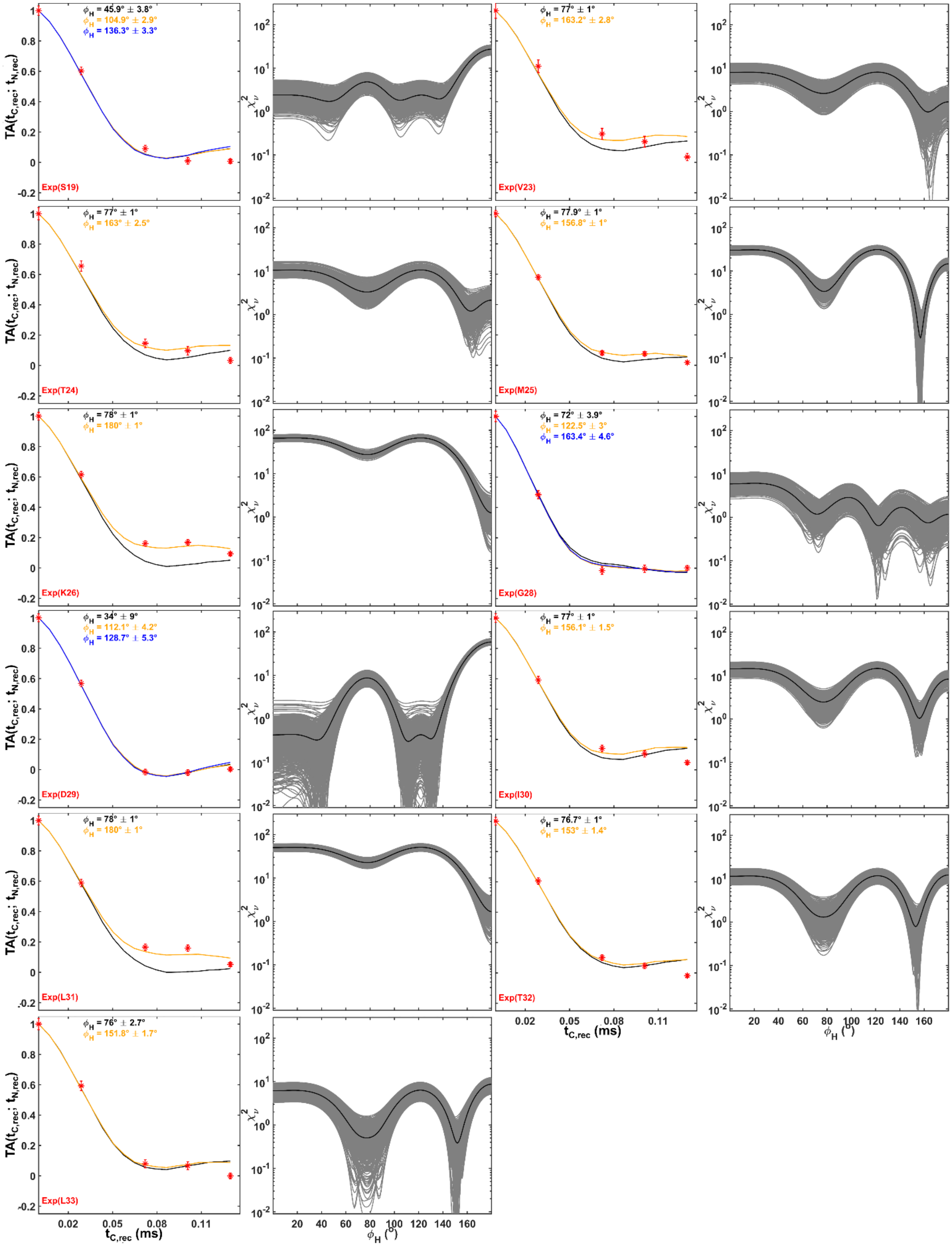

φH = 45.9° ± 3.8°
φH = 104.9° ± 2.9°
φH = 136.3° ± 3.3°
Exp(S19)
φH = 77° ± 1°
φH = 163.2° ± 2.8°
Exp(V23)
φH = 77° ± 1°
φH = 163° ± 2.5°
Exp(T24)
φH = 77.9° ± 1°
φH = 156.8° ± 1°
Exp(M25)
φH = 78° ± 1°
φH = 180° ± 1°
Exp(K26)
φH = 72° ± 3.9°
φH = 122.5° ± 3°
φH = 163.4° ± 4.6°
Exp(G28)
φH = 34° ± 9°
φH = 112.1° ± 4.2°
φH = 128.7° ± 5.3°
Exp(D29)
φH = 77° ± 1°
φH = 156.1° ± 1.5°
Exp(I30)
φH = 78° ± 1°
φH = 180° ± 1°
Exp(L31)
φH = 76.7° ± 1°
φH = 153° ± 1.4°
Exp(T32)
φH = 76° ± 2.7°
φH = 151.8° ± 1.7°
Exp(L33)
TA(tC,rec; tN,rec)
χ²ν
tC,rec (ms)
φH (°)

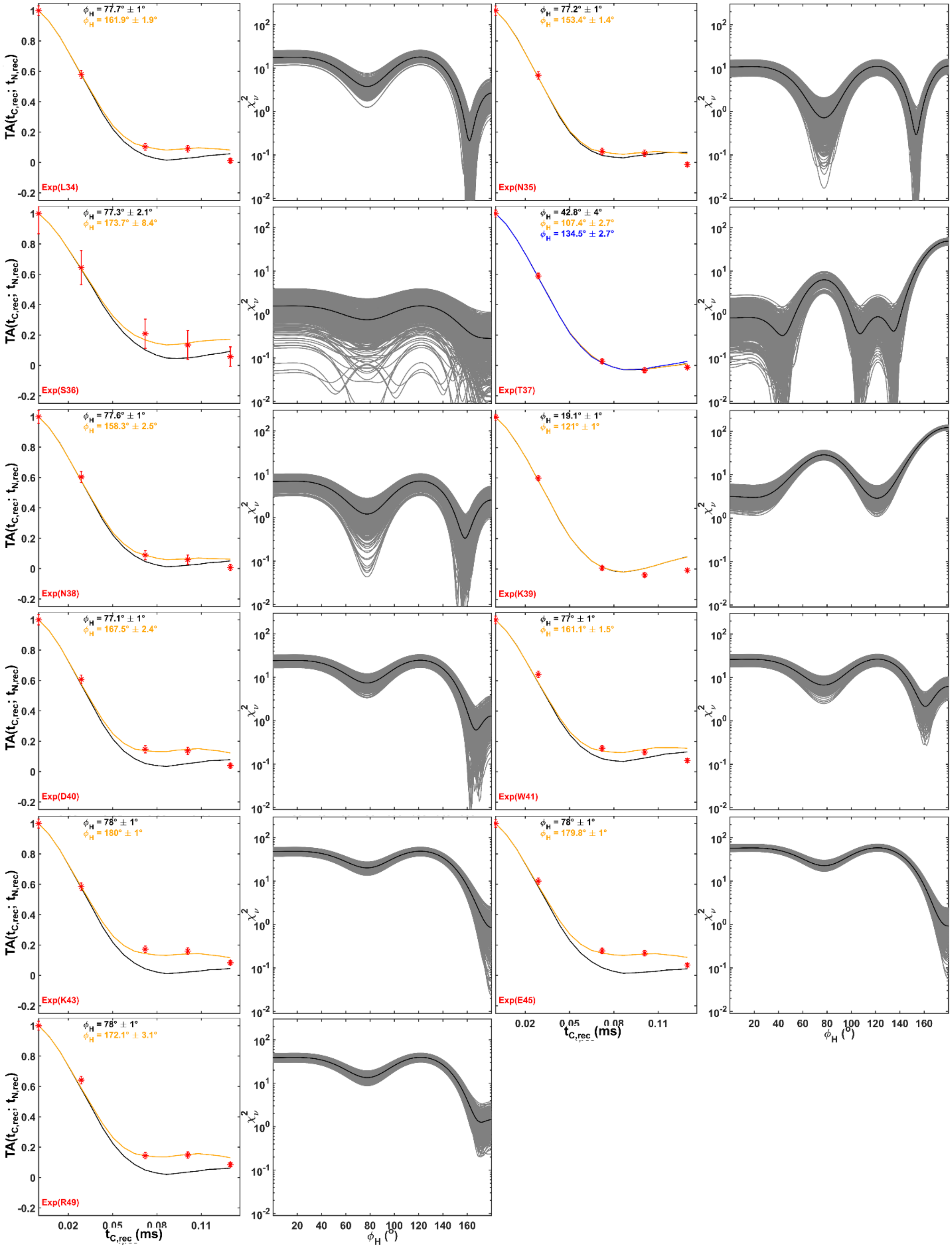

Exp(L34)
ϕ_H = 77.7° ± 1°
ϕ_H = 161.9° ± 1.9°
Exp(N35)
ϕ_H = 77.2° ± 1°
ϕ_H = 153.4° ± 1.4°
Exp(S36)
ϕ_H = 77.3° ± 2.1°
ϕ_H = 173.7° ± 8.4°
Exp(T37)
ϕ_H = 42.8° ± 4°
ϕ_H = 107.4° ± 2.7°
ϕ_H = 134.5° ± 2.7°
Exp(N38)
ϕ_H = 77.6° ± 1°
ϕ_H = 158.3° ± 2.5°
Exp(K39)
ϕ_H = 19.1° ± 1°
ϕ_H = 121° ± 1°
Exp(D40)
ϕ_H = 77.1° ± 1°
ϕ_H = 167.5° ± 2.4°
Exp(W41)
ϕ_H = 77° ± 1°
ϕ_H = 161.1° ± 1.5°
Exp(K43)
ϕ_H = 78° ± 1°
ϕ_H = 180° ± 1°
Exp(E45)
ϕ_H = 78° ± 1°
ϕ_H = 179.8° ± 1°
Exp(R49)
ϕ_H = 78° ± 1°
ϕ_H = 172.1° ± 3.1°
TA($t_{C,rec}$; $t_{N,rec}$)
χ^2_ν
$t_{C,rec}$ (ms)
ϕ_H (°)

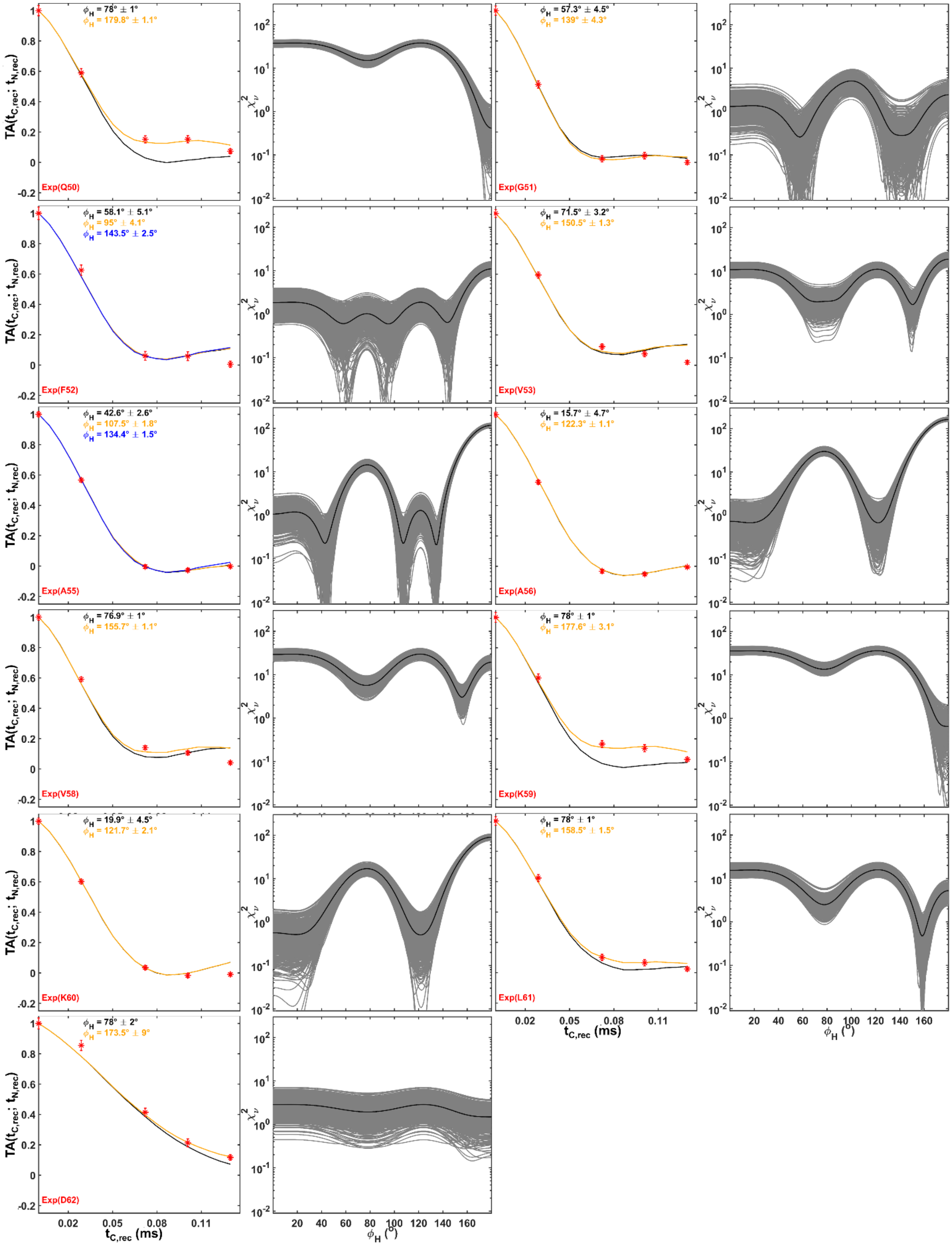

ϕ_H = 78° ± 1°
ϕ_H = 179.8° ± 1.1°
Exp(Q50)
ϕ_H = 57.3° ± 4.5°
ϕ_H = 139° ± 4.3°
Exp(G51)
ϕ_H = 58.1° ± 5.1°
ϕ_H = 95° ± 4.1°
ϕ_H = 143.5° ± 2.5°
Exp(F52)
ϕ_H = 71.5° ± 3.2°
ϕ_H = 150.5° ± 1.3°
Exp(V53)
ϕ_H = 42.6° ± 2.6°
ϕ_H = 107.5° ± 1.8°
ϕ_H = 134.4° ± 1.5°
Exp(A55)
ϕ_H = 15.7° ± 4.7°
ϕ_H = 122.3° ± 1.1°
Exp(A56)
ϕ_H = 76.9° ± 1°
ϕ_H = 155.7° ± 1.1°
Exp(V58)
ϕ_H = 78° ± 1°
ϕ_H = 177.6° ± 3.1°
Exp(K59)
ϕ_H = 19.9° ± 4.5°
ϕ_H = 121.7° ± 2.1°
Exp(K60)
ϕ_H = 78° ± 1°
ϕ_H = 158.5° ± 1.5°
Exp(L61)
ϕ_H = 78° ± 2°
ϕ_H = 173.5° ± 9°
Exp(D62)
TA($t_{C,rec}$; $t_{N,rec}$)
χ^2_ν
$t_{C,rec}$ (ms)
ϕ_H (°)

Figure S23 Experimental SH3 (with windowed CP condition of 90°(0.5ν_R)/ 450°(2.5ν_R)) and simulated **TA curves for torsion angle values**. The data was acquired on a 800 MHz spectrometer. The simulated pMODERN curves were obtained using the $\Delta\alpha_{rf,max}$ values (summarized in Figures S10A and S10B, red circles) and $T_{2,eff} = 0.566$ ms for better fitting. The fitting errors were obtained by generating 200 Monte Carlo curves, assuming a Gaussian noise distribution (2σ). In all plots, the last experimental point at 0.1296 ms of $t_{1,rec}$ was excluded from the quantitative analysis of the TA curves and the determination of ϕ_H values.

Dipolar and torsion angle S31N M2 curves

Figures 24, 25 and 26 show the experimental and simulated Dip CH, Dip NH and TA curves for CP condition with windowed shape: 270°(1.5ν_R)/ 630°(1.75ν_R).

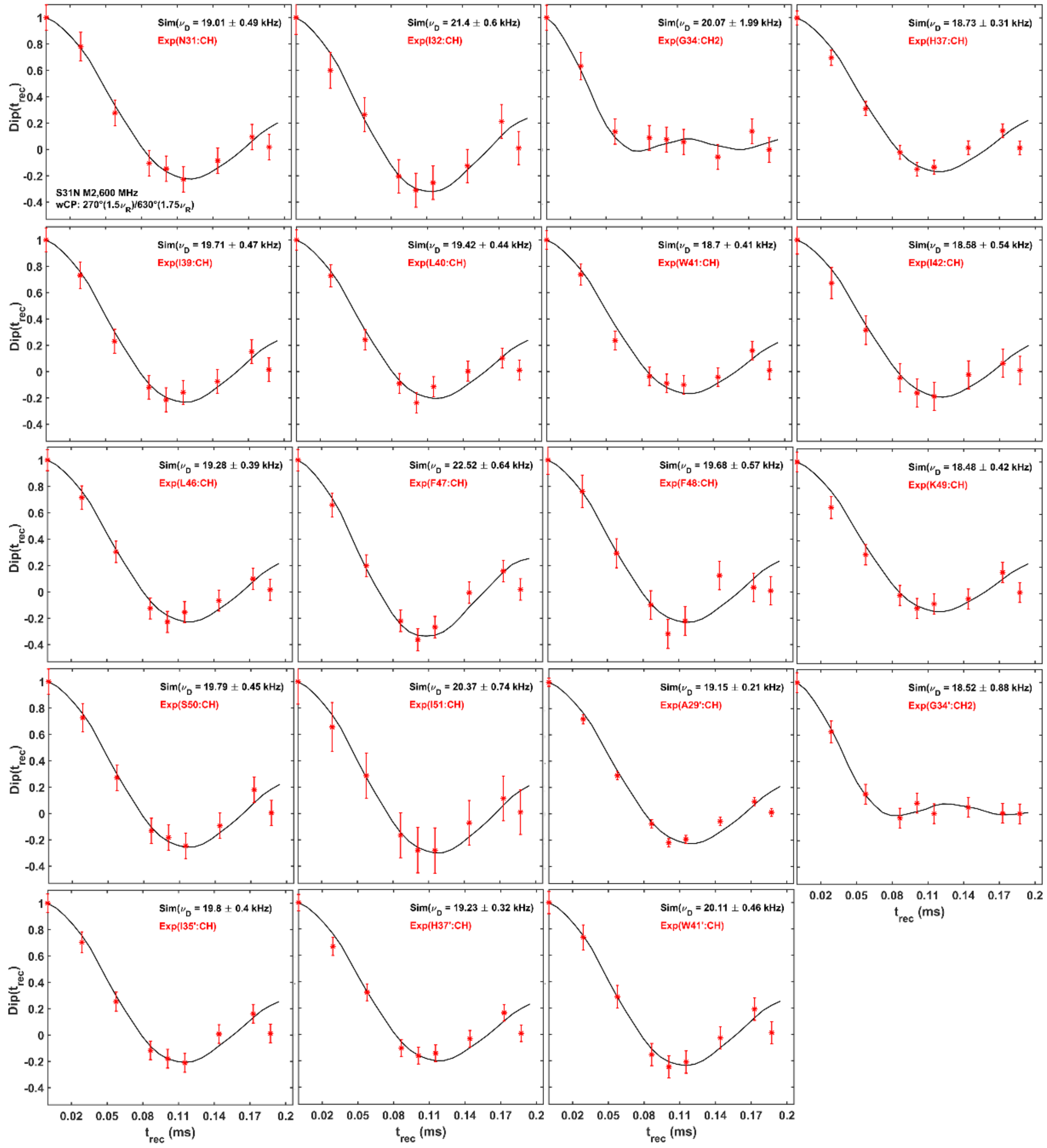

Figure S24 Experimental S31N M2 (with windowed CP condition of 270°(1.5ν_R)/ 630°(1.75ν_R)) and simulated **Dip curves for CH dipolar coupling values**. The data was acquired on a 600 MHz spectrometer. The simulated pMODERN curves were obtained using the $\Delta\alpha_{rf,max}$ values (summarized in Figure S11A, black stars) and $T_{2,eff}$ = 0.555 ms for better fitting. The fitting errors were obtained by generating 200 Monte Carlo curves, assuming a Gaussian noise distribution (2σ).

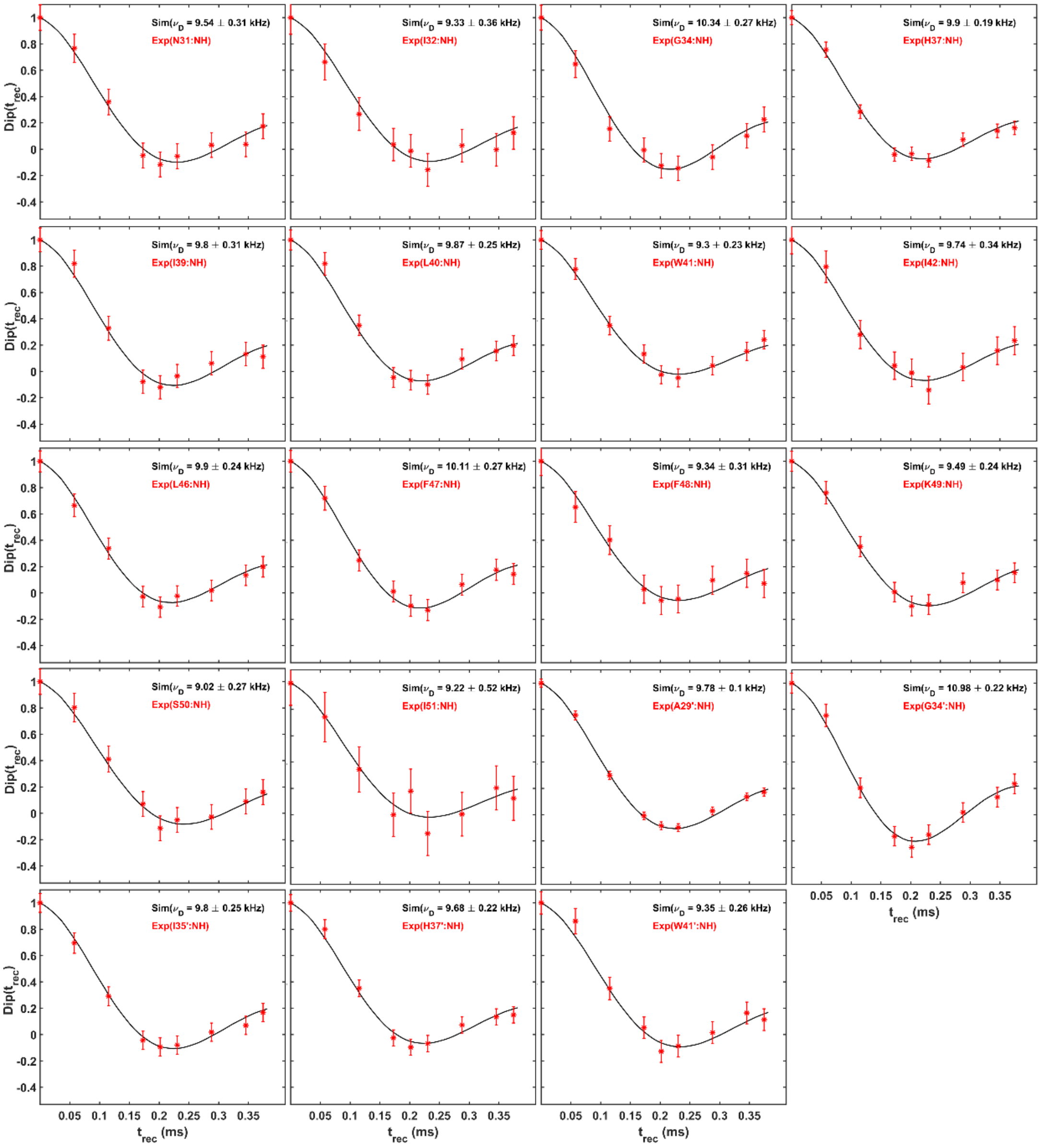

Figure S25 Experimental S31N M2 (with windowed CP condition of 270°(1.5ν_R)/ 630°(1.75ν_R)) and simulated **Dip curves for HN dipolar coupling values**. The data was acquired on a 600 MHz spectrometer. The simulated pMODERN curves were obtained using the $\Delta\alpha_{rf,max}$ values (summarized in Figure S11B, black stars) and $T_{2,eff}$ = 0.555 ms for better fitting. The fitting errors were obtained by generating 200 Monte Carlo curves, assuming a Gaussian noise distribution (2σ).

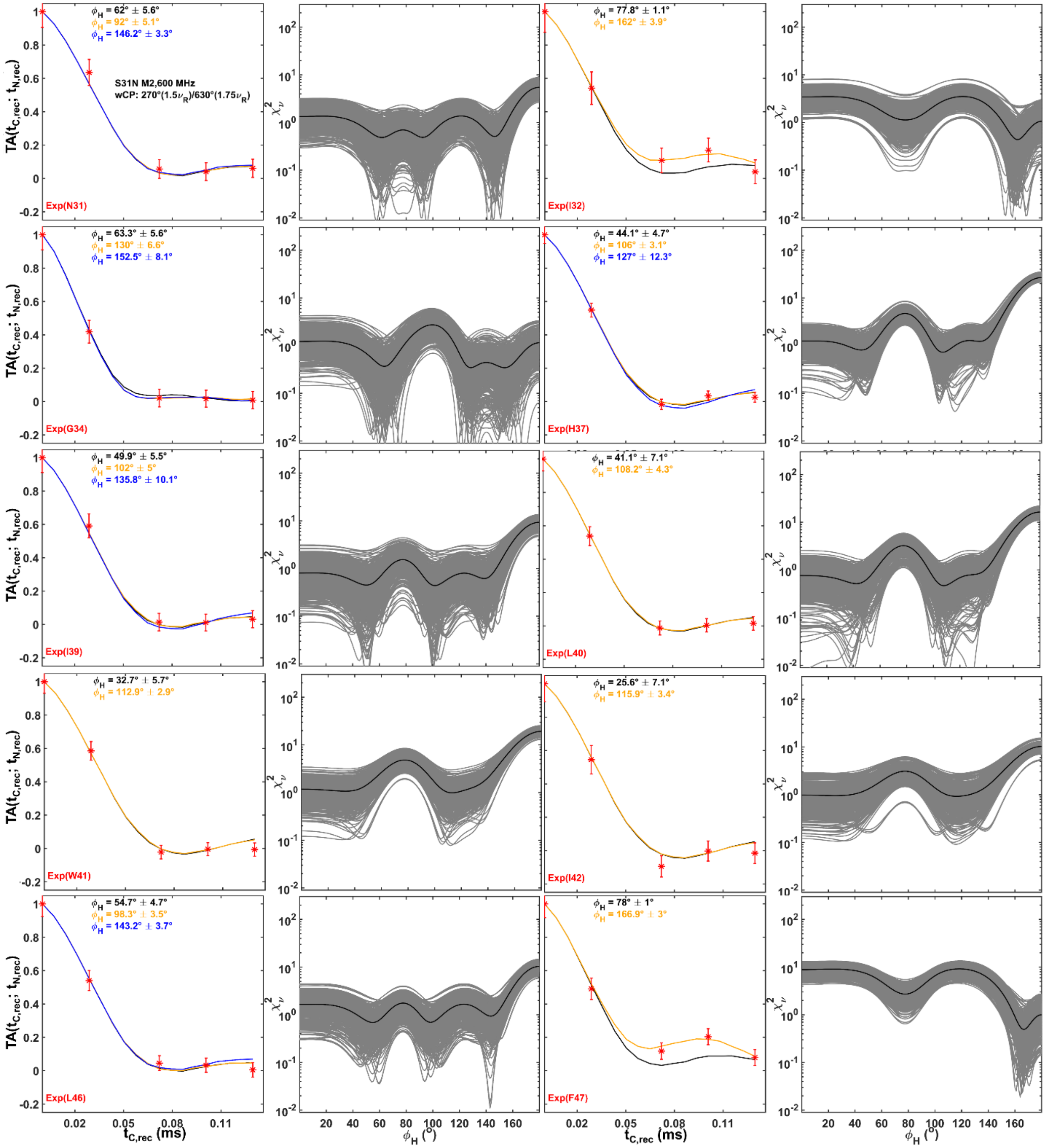

ϕH = 62° ± 5.6°
ϕH = 92° ± 5.1°
ϕH = 146.2° ± 3.3°
S31N M2,600 MHz
wCP: 270°(1.5νR)/630°(1.75νR)
Exp(N31)
ϕH = 77.8° ± 1.1°
ϕH = 162° ± 3.9°
Exp(I32)
ϕH = 63.3° ± 5.6°
ϕH = 130° ± 6.6°
ϕH = 152.5° ± 8.1°
Exp(G34)
ϕH = 44.1° ± 4.7°
ϕH = 106° ± 3.1°
ϕH = 127° ± 12.3°
Exp(H37)
ϕH = 49.9° ± 5.5°
ϕH = 102° ± 5°
ϕH = 135.8° ± 10.1°
Exp(I39)
ϕH = 41.1° ± 7.1°
ϕH = 108.2° ± 4.3°
Exp(L40)
ϕH = 32.7° ± 5.7°
ϕH = 112.9° ± 2.9°
Exp(W41)
ϕH = 25.6° ± 7.1°
ϕH = 115.9° ± 3.4°
Exp(I42)
ϕH = 54.7° ± 4.7°
ϕH = 98.3° ± 3.5°
ϕH = 143.2° ± 3.7°
Exp(L46)
ϕH = 78° ± 1°
ϕH = 166.9° ± 3°
Exp(F47)
TA(tC,rec; tN,rec)
tC,rec (ms)
χ²ν
ϕH (°)

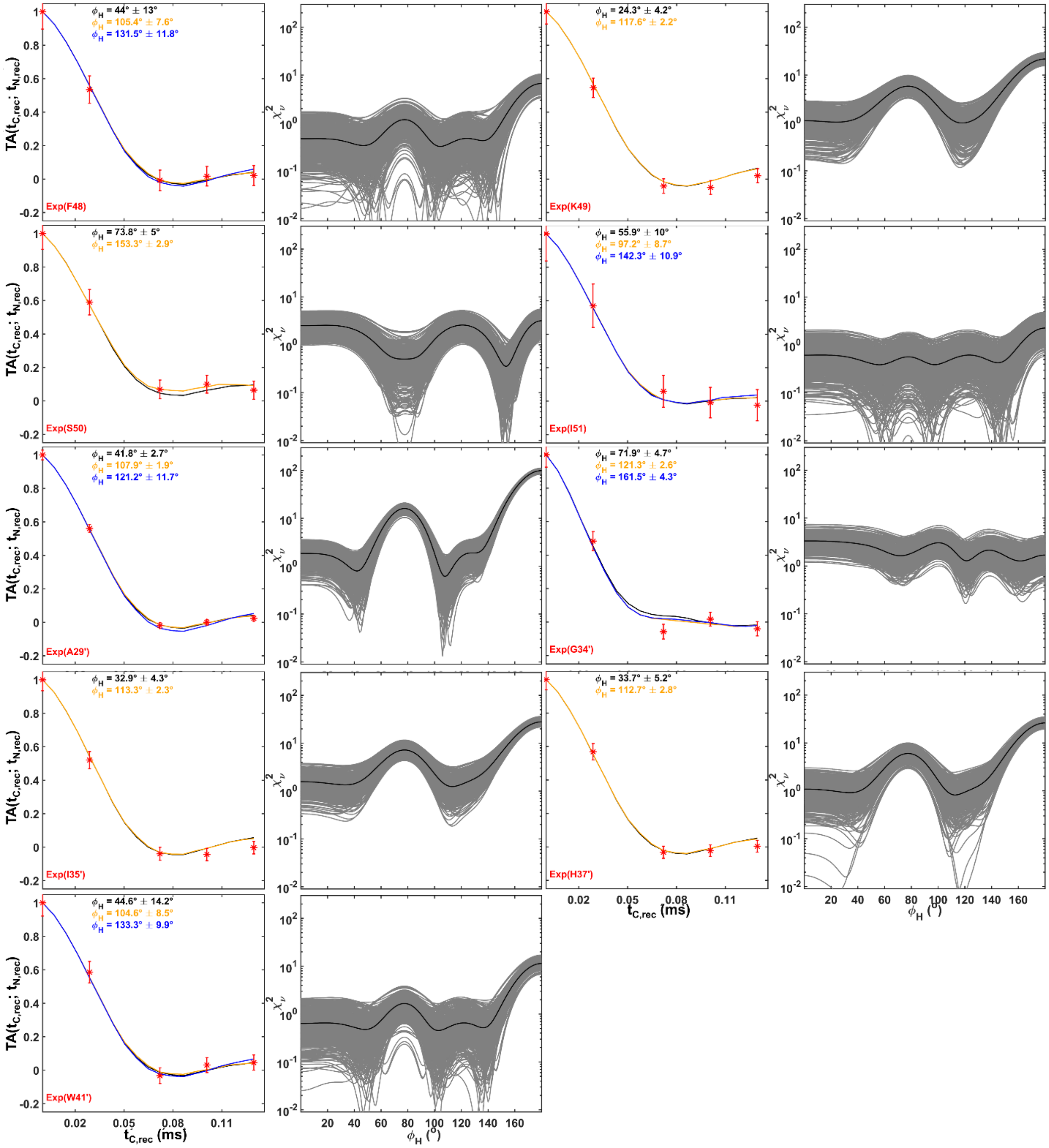

Figure S26 Experimental S31N M2 (with windowed CP condition of 270°($1.5\nu_R$)/ 630°($1.75\nu_R$)) and simulated **TA curves for torsion angle values**. The data was acquired on a 600 MHz spectrometer. The simulated pMODERN curves were obtained using the $\Delta\alpha_{rf,max}$ values (summarized in Figures S11A and 11B, black stars) and $T_{2,eff}$ = 0.555 ms for better fitting. The fitting errors were obtained by generating 200 Monte Carlo curves, assuming a Gaussian noise distribution (2σ).

Figures 27, 28 and 29 show the experimental and simulated Dip CH, Dip NH and TA curves for CP condition with windowed shape: 90°(0.5ν_R)/ 450°(2. 5ν_R).

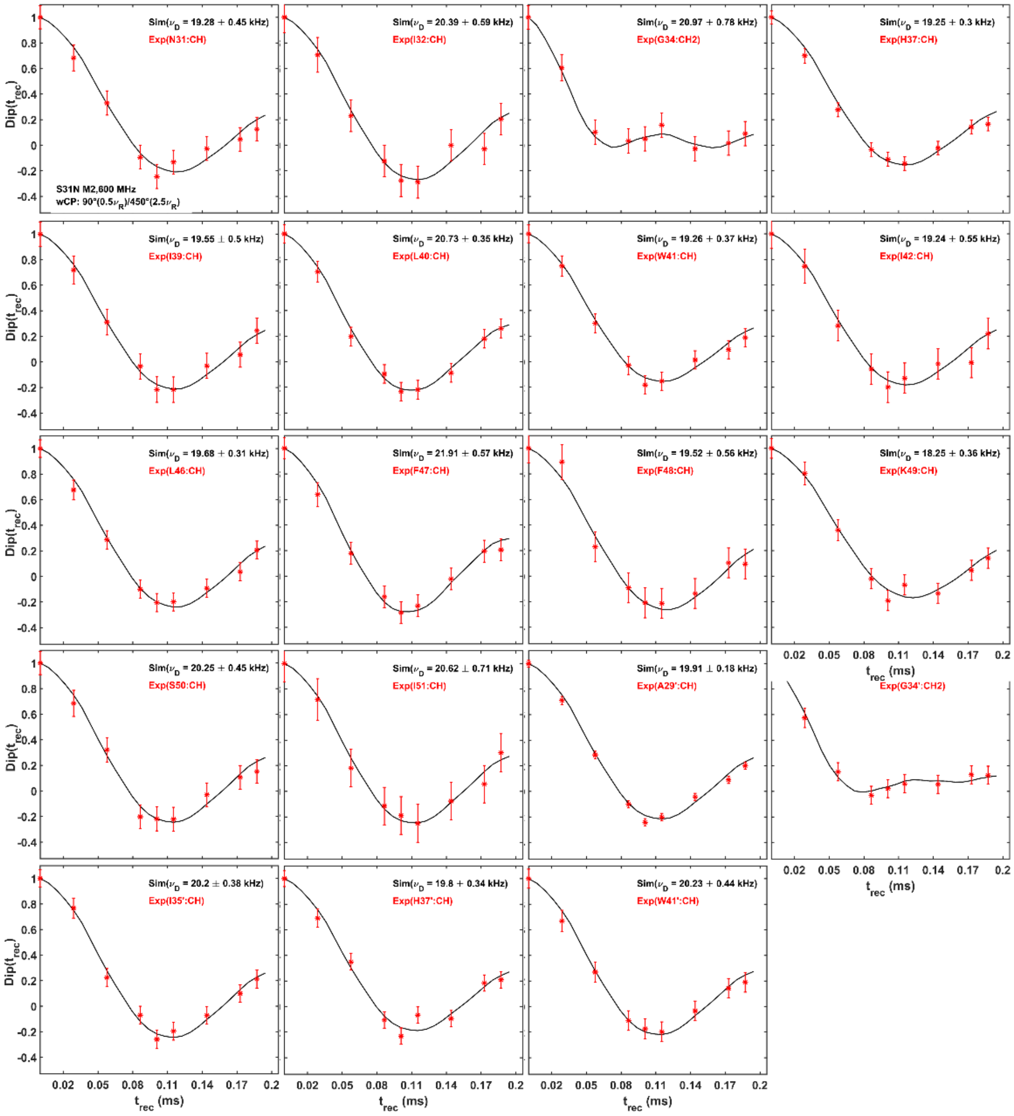

Figure S27 Experimental S31N M2 (with windowed CP condition of 90°(0.5ν_R)/ 450°(2.5ν_R)) and simulated **Dip curves for CH dipolar coupling values**. The data was acquired on a 600 MHz spectrometer. The simulated pMODERN curves were obtained using the $\Delta\alpha_{rf,max}$ values (summarized in Figure S11A, blue diamonds) and $T_{2,eff}$ = 0.596 ms for better fitting. The fitting errors were obtained by generating 200 Monte Carlo curves, assuming a Gaussian noise distribution (2σ).

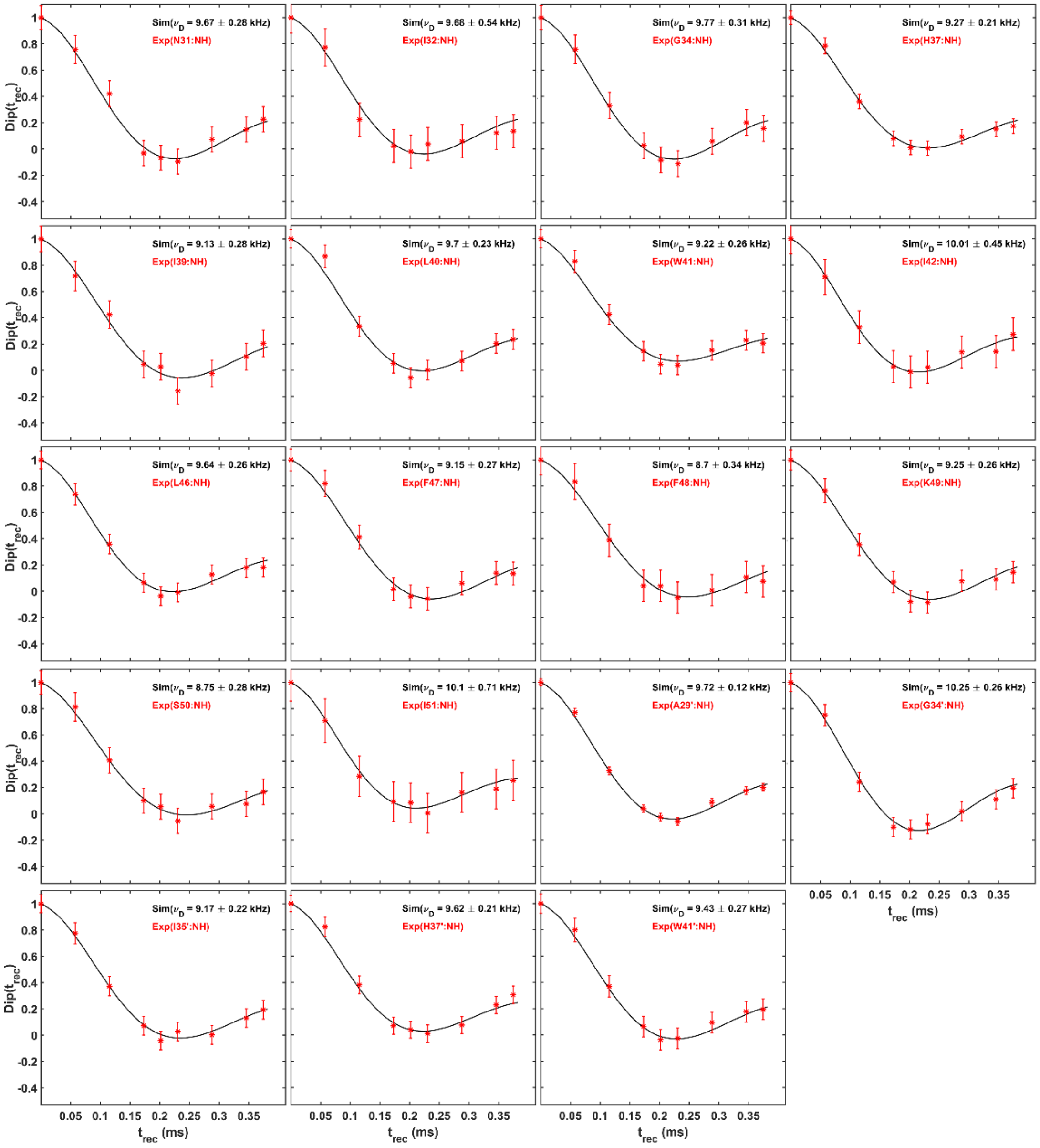

Figure S28 Experimental S31N M2 (with windowed CP condition of 90°(0.5ν_R)/ 450°(2.5ν_R)) and simulated **Dip curves for HN dipolar coupling values**. The data was acquired on a 600 MHz spectrometer. The simulated pMODERN curves were obtained using the $\Delta\alpha_{rf,max}$ values (summarized in Figure S11B, blue diamonds) and $T_{2,eff}$ = 0.596 ms for better fitting. The fitting errors were obtained by generating 200 Monte Carlo curves, assuming a Gaussian noise distribution (2σ).

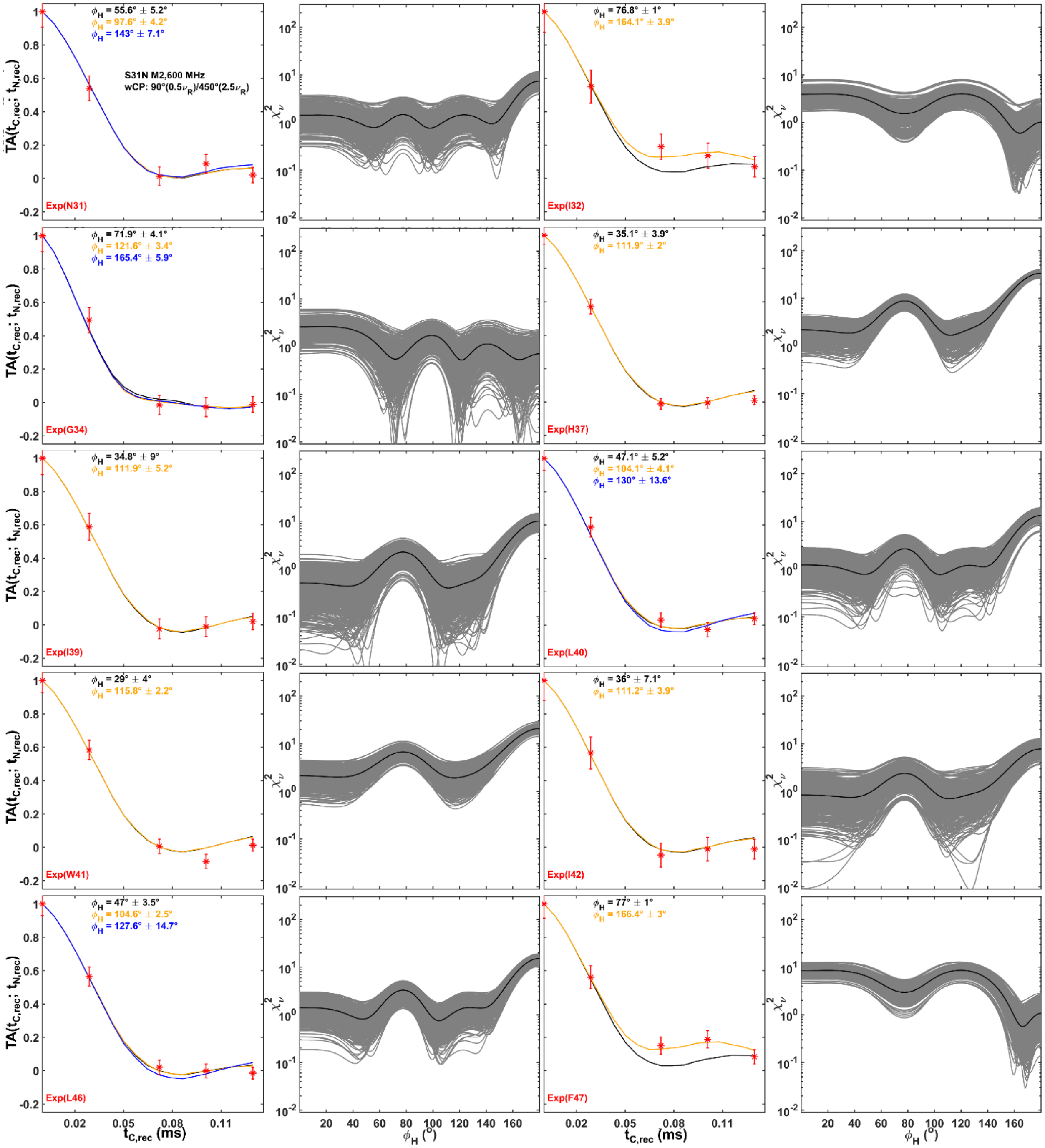
S31N M2,600 MHz
wCP: 90°(0.5ν_R)/450°(2.5ν_R)
ϕ_H = 55.6° ± 5.2°
ϕ_H = 97.6° ± 4.2°
ϕ_H = 143° ± 7.1°
Exp(N31)
ϕ_H = 76.8° ± 1°
ϕ_H = 164.1° ± 3.9°
Exp(I32)
ϕ_H = 71.9° ± 4.1°
ϕ_H = 121.6° ± 3.4°
ϕ_H = 165.4° ± 5.9°
Exp(G34)
ϕ_H = 35.1° ± 3.9°
ϕ_H = 111.9° ± 2°
Exp(H37)
ϕ_H = 34.8° ± 9°
ϕ_H = 111.9° ± 5.2°
Exp(I39)
ϕ_H = 47.1° ± 5.2°
ϕ_H = 104.1° ± 4.1°
ϕ_H = 130° ± 13.6°
Exp(L40)
ϕ_H = 29° ± 4°
ϕ_H = 115.8° ± 2.2°
Exp(W41)
ϕ_H = 36° ± 7.1°
ϕ_H = 111.2° ± 3.9°
Exp(I42)
ϕ_H = 47° ± 3.5°
ϕ_H = 104.6° ± 2.5°
ϕ_H = 127.6° ± 14.7°
Exp(L46)
ϕ_H = 77° ± 1°
ϕ_H = 166.4° ± 3°
Exp(F47)
TA($t_{C,rec}$; $t_{N,rec}$)
χ^2_ν
$t_{C,rec}$ (ms)
ϕ_H (°)

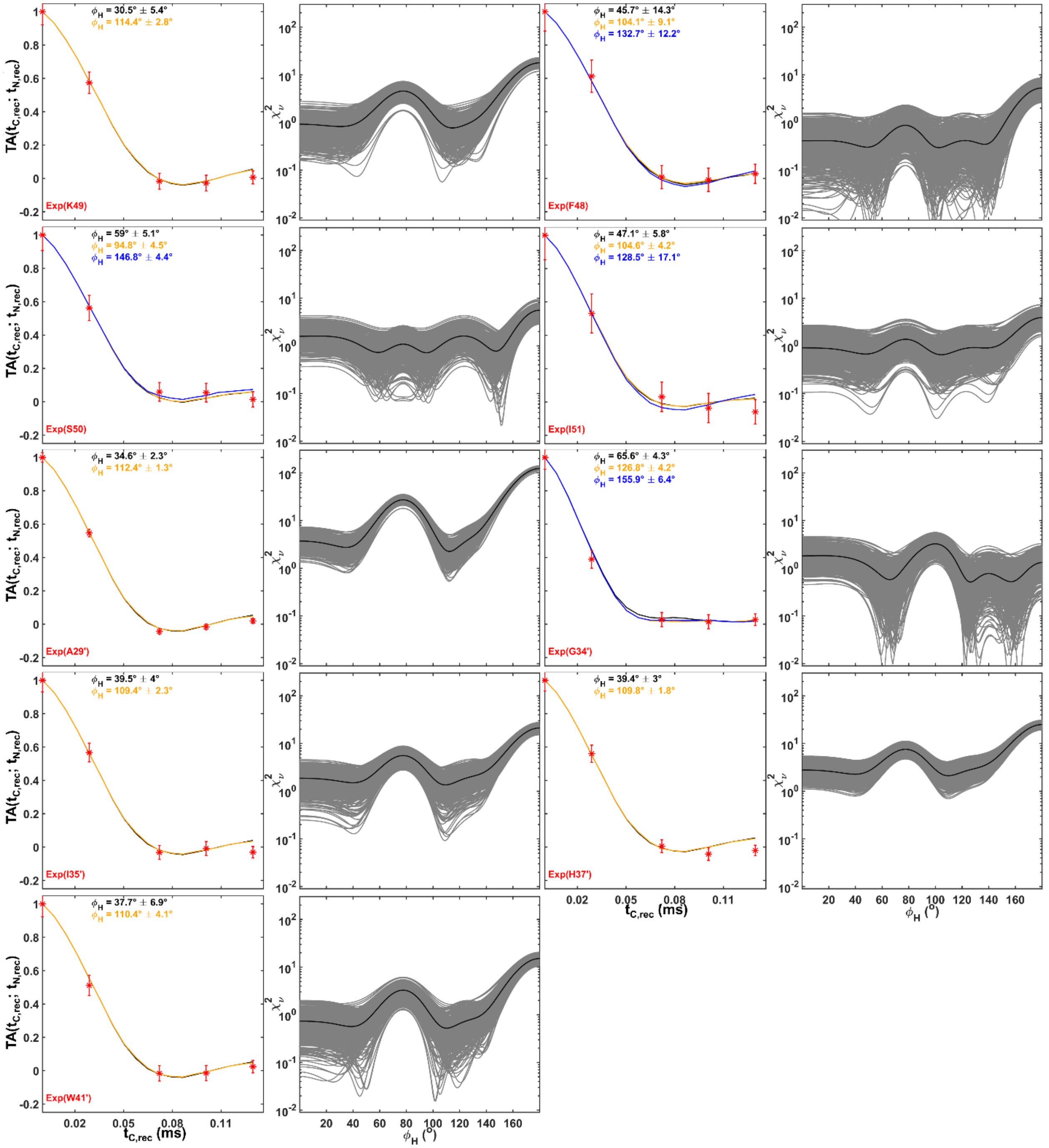

Figure S29 Experimental S31N M2 (with windowed CP condition of 90°(0.5ν_R)/ 450°(2.5ν_R)) and simulated **TA curves for torsion angle values**. The data was acquired on a 600 MHz spectrometer. The simulated pMODERN curves were obtained using the $\Delta\alpha_{rf,max}$ values (summarized in Figures S11A and 11B, blue diamonds) and $T_{2,eff}$ = 0.596 ms for better fitting. The fitting errors were obtained by generating 200 Monte Carlo curves, assuming a Gaussian noise distribution (2σ).

Additional simulations

Figure S30 compares TA curves with $\phi_H = 180°$ (green) and $\phi_H = 120°$ (black) under four different N→H CP conditions, where C has either one dipolar-coupled spin (dash-dot lines) or four dipolar-coupled spins (solid lines).

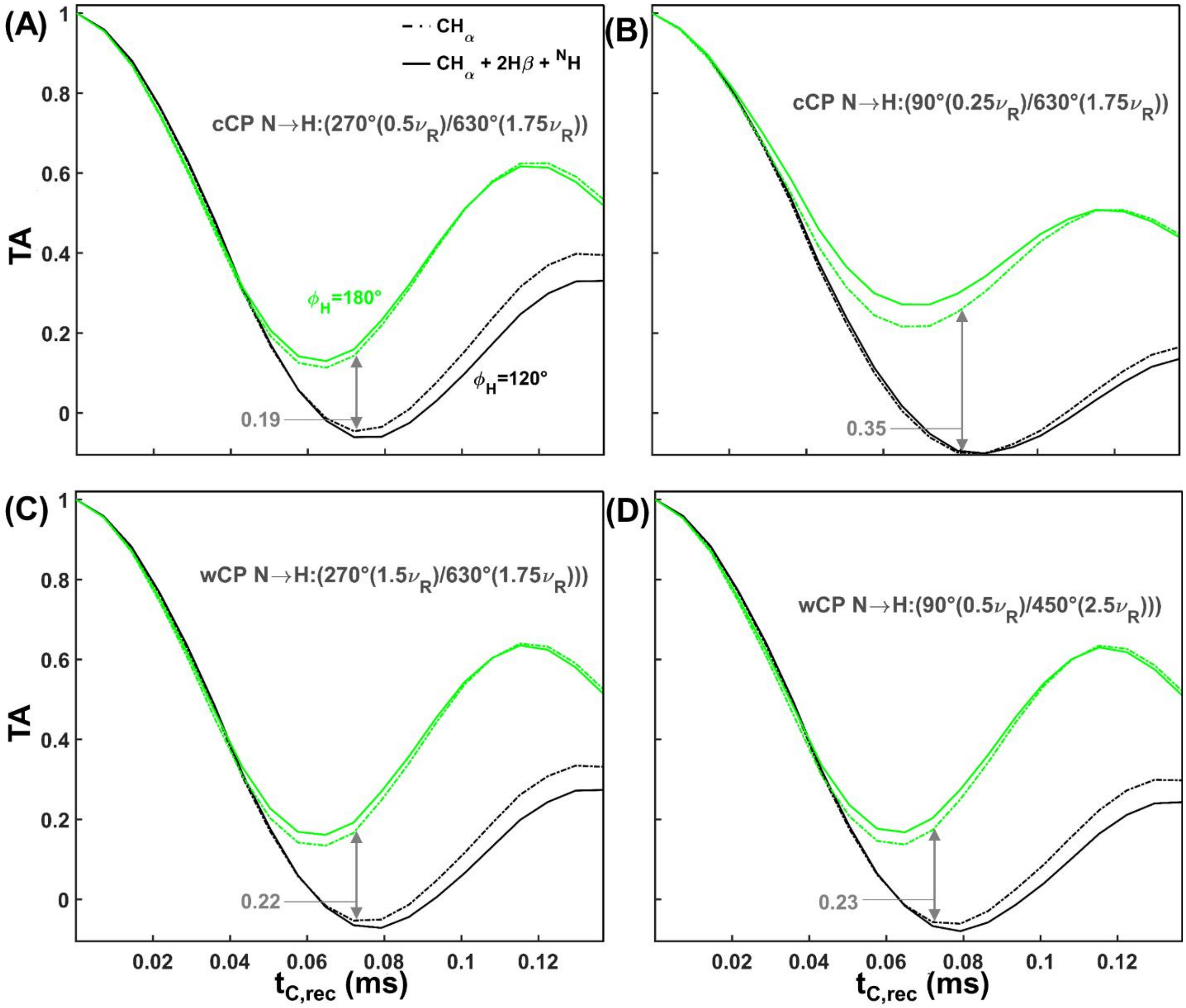

Figure S30 Torsion curves with $\phi_H = 180°$ (green) and $\phi_H = 120°$ (black) are shown under different N→H CP conditions with continuous (A-B) and half-windowed (C-D) shapes. For CH, either a single dipolar-coupled spin with a dipolar coupling of 20 kHz (dash-dot curves) or with additional dipolar-coupled proton spins – two Hβ and one ^{N}H spins – from the same amino-acid residues, each with similar dipolar coupling values of 3.1 kHz (solid curves) are considered during the first H→C CP transfer.

Figures S31 and S32 compare the CH and NH Dip orientation plots (the response of individual orientation-dependent signals as a function of recoupling time and β) under ideal case (A) and under two cCP (B-C) and wCP conditions (D) for N→H transfer. Note that the signal also depends on the α angle, which is varied together with the β angle and explains the fine structure of the plots. The ϕ_H value was set to 150° with respect to CH dipolar interaction in Figure S30, while in Figure S31 it was set with respect to NH dipolar interaction.

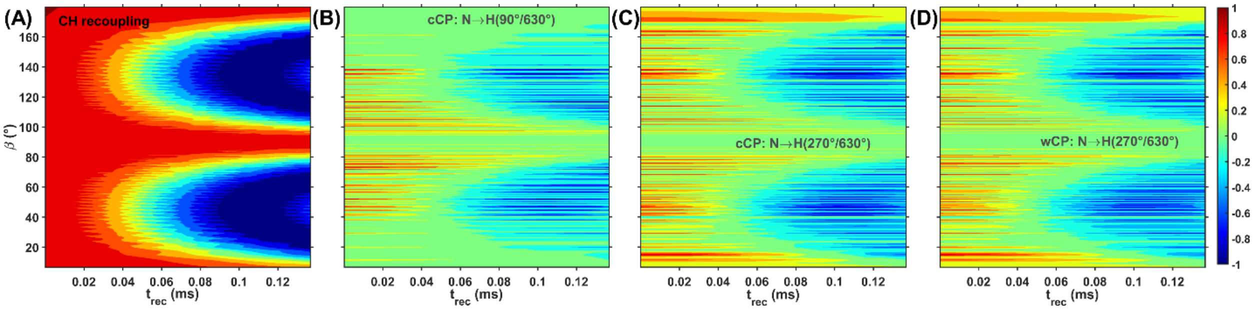

Figure S31 Simulated CH dipolar orientation plots are provided for ϕ_H =150° with respect to CH dipolar interaction. The orientation amplitudes are shown as function of recoupling time and β angle for the ideal case (A) and in the presence of three CP elements for H→C, C→N and N→H transfers (B)-(D). In (B) a 90°($0.25\nu_R$)/ 630°($1.75\nu_R$) continuous CP condition was used for the N→H transfer. In (C) 270°($0.75\nu_R$)/ 630°($1.75\nu_R$) continuous CP condition for the N→H transfer. In (D) 270°($1.5\nu_R$)/ 630°($1.75\nu_R$) windowed CP condition for the N→H transfer was used. For the rest CP elements, an [80:100](%) ramped CP with a duration of 720 µs and a [100:80](%) ramped CP with a duration of 630 µs were used to connect the H spin with C and the N spin with H, respectively. A SPEPS element with a duration of 2.88 ms was used to connect C with N. For H→C and C→N transfers, $1.75\nu_R$/ $0.75\nu_R$ and $0.25\nu_R$/ $0.75\nu_R$ CP conditions were used, respectively. In the simulations, a six-spin system was considered: one Hα, two Hβ, one Cα, one N and one ^{N}H. In all simulations, only directly bonded spins (Hα for Cα and ^{N}H for N) are taken into account during the pMODERN sequences. Note that the signal also depends on the α angle, which is varied together with the β angle and explains the fine structure of the plots.

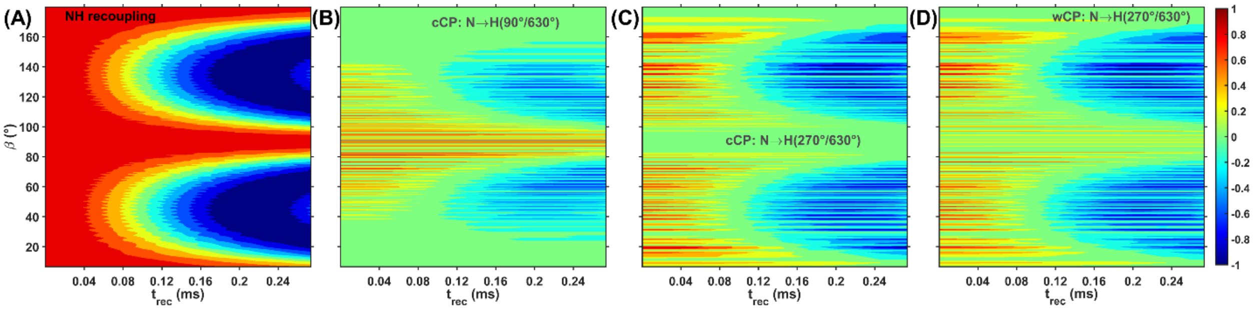

Figure S32 Simulated NH dipolar orientation plots are provided for ϕ_H =150° with respect to NH dipolar interaction. The remaining simulation details are the same as those in Figure S31. Note that the signal also depends on the α angle, which is varied together with the β angle and explains the fine structure of the plots.

^{1}H CSA has an impact on TA curves at longer mixing times, similar to the simulations in which additional dipolar-coupled spins were included during the pMODERN sequences (Figure 10 in the main text). This is another factor that caused the experimental TA curves to converge toward the x-axis at longer mixing time.

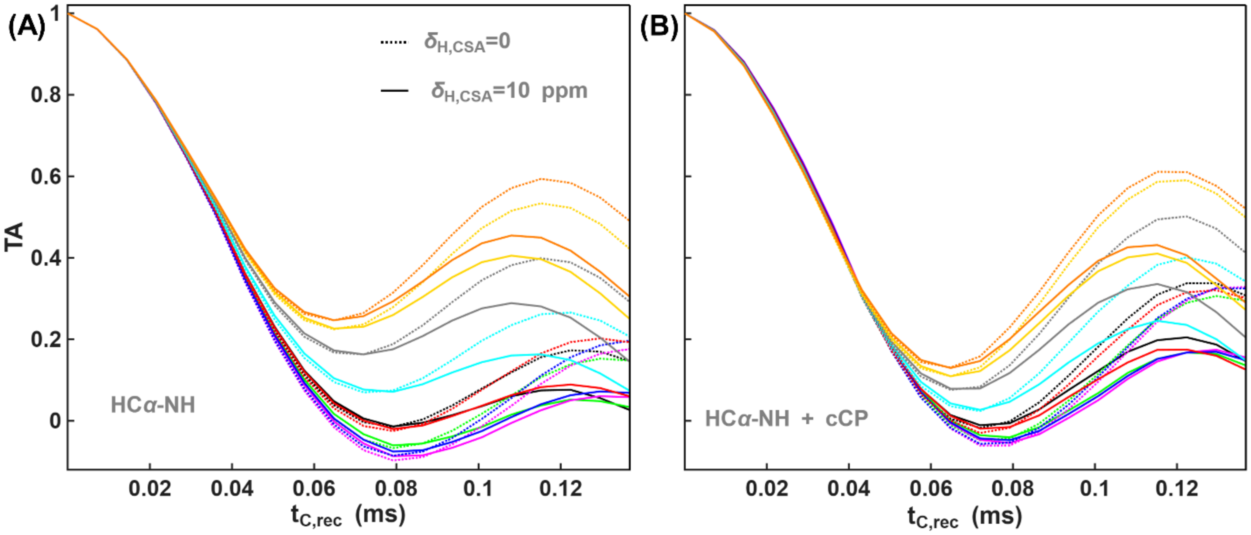

Figure S33 Simulated torsion angle curves without (round dots) and with ^{1}H CSA (solid lines) are shown for the ideal case (A) and in the presence of continuous CP elements (B). Both directly bonded Hα and ^{N}H spins had the same reduced anisotropy (δ_{CSA}) value of 10 ppm (6 kHz at a 600 MHz spectrometer, Haeberlen convention). The asymmetry parameters were 0.7 and 0.1, respectively, and the CSA orientations were [0°,30°,50°] and [150°,140°,10°], respectively, relative to the heteronuclear Hα-C dipolar interaction. In the simulations, a six-spin system was considered: one Hα, two Hβ, one Cα, one N and one ^{N}H. Only directly bonded spins (Hα for Cα and ^{N}H for N) were taken into account during the pMODERN sequences. For H→C transfers, the following ramp was applied on the proton channel: an [80:100](%) with a duration of 720 μs and RF-field conditions of $1.75\nu_R/0.75\nu_R$. For N→H transfers, the following ramp was applied a [100:80](%) with a duration of 630 μs and RF-field conditions of $0.75\nu_R/1.75\nu_R$. For the C→N transfer, a SPEPS element was used with a duration of 2.88 ms and RF-

field conditions of $0.25\nu_R/0.75\nu_R$. For all CP elements, the mentioned values in MAS units represent the RF-field strengths at the midpoint of the applied shapes. The MAS rate was 55.555 kHz.

Simulated TA curves at six different MAS rates, both without (ideal, dashed) and with continuous CP elements (solid) are shown below.

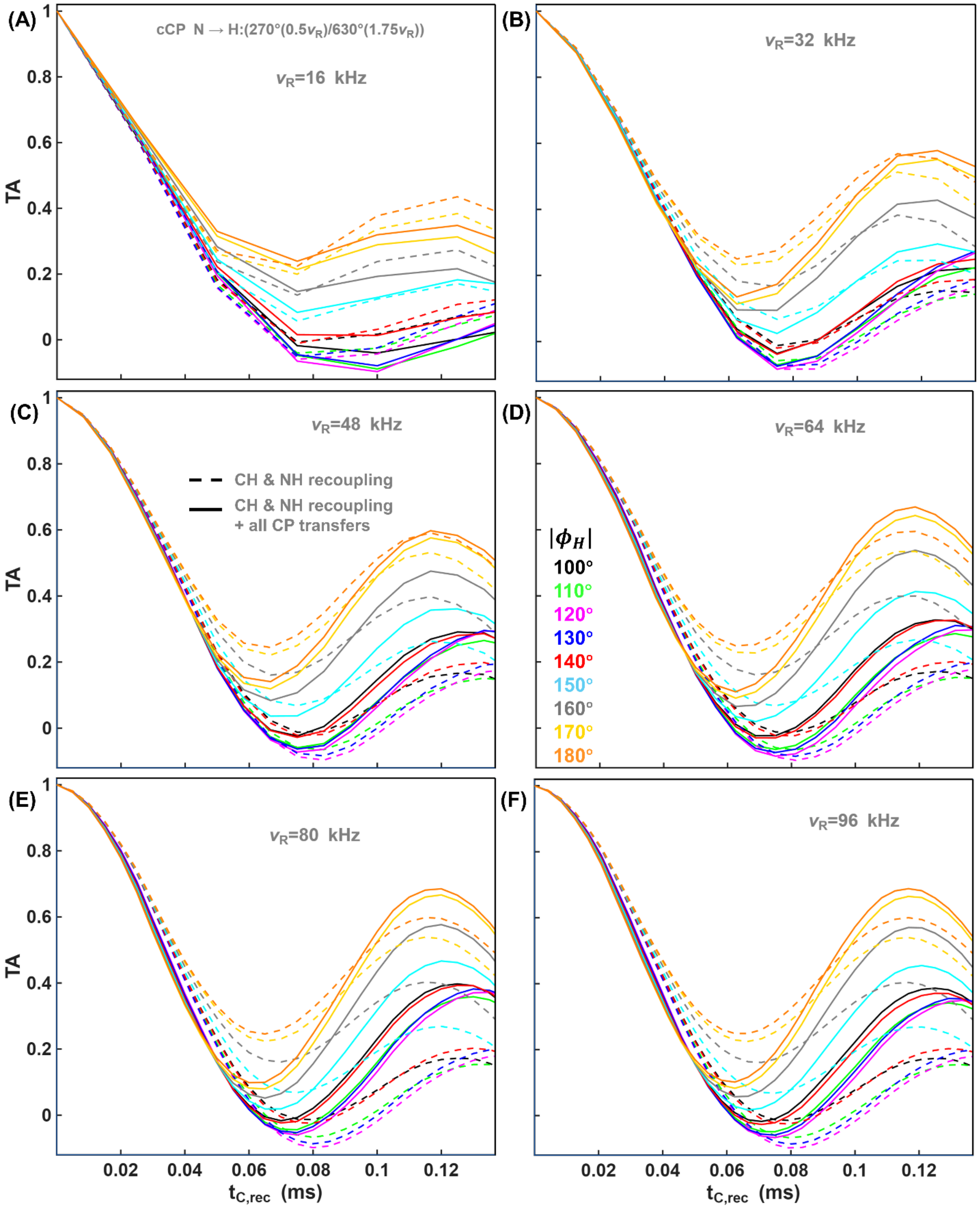
(A)
cCP N → H:(270°(0.5νR)/630°(1.75νR))
νR=16 kHz
(B)
νR=32 kHz
(C)
νR=48 kHz
CH & NH recoupling
CH & NH recoupling
+ all CP transfers
(D)
νR=64 kHz
|ϕH|
100°
110°
120°
130°
140°
150°
160°
170°
180°
(E)
νR=80 kHz
(F)
νR=96 kHz
TA
tC,rec (ms)

Figure S34 Simulated torsion angle curves in the ideal case (dashed lines) and with continuous CP elements (solid lines) at different MAS rates, ν_R, as indicated. In the simulations, a six-spin system was considered: one Hα, two Hβ, one Cα, one N and one ^{N}H. In all simulations, only directly bonded spins (Hα for Cα and ^{N}H for N) are taken into account during the pMODERN sequences. For H→C transfers, an 80 to 100% ramp was applied on the proton channel, centered an RF-field condition of $1.75\nu_R/0.75\nu_R$. The CP duration was 1125 µs. For N→H transfers, a 100 to 80% ramp was appliet, centered at the $0.75\nu_R/1.75\nu_R$ condition. The duration was 625 µs. For the C→N transfer, a SPEPS element at the $0.25\nu_R/0.75\nu_R$ condition was used with a duration of around 2 ms.

Experimental methods

Simulations

MODERN and pMODERN simulations were performed using in-house MATLAB scripts with the numerical solution of the equation of motion.[13]

Sample Preparation

Fully protonated microcrystalline chicken alpha-spectrin SH3 was prepared according to the protocols in the next references[14,15] and influenza A M2 protein, residues 18-60, was prepared according to the protocols in the references [[16,17]]. Both samples contained containing Cu^{2+} ethylenediaminetetraacetic (EDTA) acid to accelerate the acquisition. Each sample was packed into a Bruker 1.3 mm rotor via centrifugation.

Solid state NMR spectroscopy

The RF-field power of pMODERN pulses was optimized using 1D (HCAN)H experiments with a pMODERN recoupling time of 0.201 ms mixing time for NH recoupling. The optimal RF-filed was identified when the measured signal reached its minimum (non-zero negative value). This recoupling time corresponds to a dipolar coupling value of ~10 kHz. The pMODERN sequences were applied as constant-time elements,[18] with a total fixed duration of 936 ms.

All experiments were performed with 55.555 kHz MAS rate and the temperature of the nitrogen cooling gas set to 245 K with 1000 to 1300 liters per hour. In all 3D (H)CANH experiments, a [80:100](%) ramped CP element with a duration of 720 μs and a [100:80](%) ramped CP element[2] with a duration of 630 μs were used to connect the ^{1}H spin with ^{13}C and the ^{15}N spin with ^{1}H, respectively. A SPEPS element[9] with a duration of 2.88 ms was used to connect ^{13}C with ^{15}N. For $^{1}H \rightarrow ^{13}C$ and $^{13}C \rightarrow ^{15}N$ transfers, $1.75\nu_R / 0.75\nu_R$ and $0.25\nu_R / 0.75\nu_R$ CP conditions were used, respectively.

All CP conditions were optimized in the same way. For the $^{1}H \rightarrow ^{13}C$ and $^{15}N \rightarrow ^{1}H$ transfers, the optimal RF-field power levels on the ^{13}C and ^{15}N channels were calculated with respect to 90° hard pulses, while the optimal ^{1}H RF-field powers were experimentally optimized. For the $^{13}C \rightarrow ^{15}N$ transfer with SPEPS element, the optimal ^{13}C RF-field power was calculated with respect to a 90° hard pulse, and the optimal ^{15}N RF-field power was experimentally optimized.

For decoupling of the heteronuclear dipolar interactions SW_f-TPPM[4] was used on the proton channel, and WALTZ-16[5] was used on heteronuclear channels. MISSISSIPPI[6] water suppression was applied with a duration of 100 ms and ~14 kHz RF-field strength.

600 MHz spectrometer 3D (H)CANH experiments with pMODERN pulses were acquired on a Bruker Avance III HD spectrometer operating at 14.1 T (600 MHz ^{1}H frequency) using a DVT600W2 BL1.3 mm HXY probe.

For the SH3 sample, the sets of 3D (H)CANH for CH, NH dipolar coupling and TA experiments were acquired with the same number scans for each recoupling time point (8 scans), with a 0.4 s delay between each scan. The total time for a single experiment was 20.22 h.

In the following Table below: SW – spectral width; TD – the number of points in the FID; IN_F – an increment time; AQ – the acquisition time; CF – Carrier Frequency. 4 Dummy Scans were used.

(H)CANH	F3	F2	F1
	^{1}H	^{15}N	^{13}C
TD	1386	136	120
SW(ppm)	46.3664	40	40
IN_F(μs)		411.78	165.96
AQ(s)	0.024948	0.0280009	0.0099569
CF	4.7	118	53.7

Table S3 3D (H)CANH experimental parameters

For the S31N M2 sample, CH and NH dipolar coupling experiments were acquired with 16 scans for each recoupling time point (0.35 s delay time between each scan), except the reference point (zero pMODERN recoupling time) which was acquired with 32 scans. The total time for a single experiment with 16 scans was 18.62 h. For TA experiments, the Table S2 summarizes number scans for each point:

	First point (reference)	Second point	Third point	Fourth point	Fifth point
$t_{1,rec}$ (μs)<br>$t_{2,rec}$ (μs)	0<br>0	28.8<br>57.6	72<br>144	100.8<br>201.6	129.6<br>259.2
NS	32	48	48	48	48
Total time	1.55 days	2.33 days	2.33 days	2.33 days	2.33 days

Table S4 The summary of scans (NS) and the total time in 3D (H)CANH torsion angle experiments

In the following Table below: SW – spectral width; TD – the number of points in the FID; IN_F – an increment time; AQ – the acquisition time; CF – Carrier Frequency. 4 Dummy Scans were used.

(H)CANH	F3	F2	F1
	^{1}H	^{15}N	^{13}C
TD	1386	88	96
SW(ppm)	60.1872	40	40
IN_F(μs)		411.78	165.96
AQ(s)	0.24948	0.280009	0.0099569
CF	4.7	118	53.7

Table S5 3D (H)CANH experimental parameters

800 MHz spectrometer: 3D (H)CANH experiments with pMODERN pulses were acquired on a Bruker Avance III HD spectrometer operating at 18.8 T (800 MHz ^{1}H field power), equipped with a 1.3 mm HCN MAS probe.

For the SH3 sample, the sets of 3D (H)CANH for CH, NH dipolar coupling and TA experiments were acquired with the same number scans for each recoupling time point (8 scans), with a 0.22 s delay between each scan. The total time for a single experiment was 11.86 h.

In the following Table below: SW – spectral width; TD – the number of points in the FID; IN_F – an increment time; AQ – the acquisition time; CF – Carrier Frequency. 4 Dummy Scans were used.

(H)CANH	F3	F2	F1

	^{1}H	^{15}N	^{13}C
TD	1024	136	112
SW(ppm)	49.6215	40	40
IN_F(μs)		308.48	124.32
AQ(s)	0.0129024	0.0209766	0.0069619
CF	4.7	122	53.53

Table S6 3D (H)CANH experimental parameters

BRUKER PULSE PROGRAM

The 3D (H)CANH sequence contains a SPEPS element, which requires Echo/Anti-Echo mode for the ^{13}C dimension (always F1!). After data acquisition, the data should be firstly processed with a conversion script. Please refer to the SPEPS articles for more details.[9,19]

The width of the SPEPS and pMODERN pulses are automatically calculated using the 'cnst31' parameter (the MAS rate in Hz). Note that there is protection against long acquisitions that occur if this parameter is mistakenly set too low. However, still use with precaution.

Short explanation of parameters: *l10* defines the number of rotor periods in the constant-time element. The total duration of the CT element is *l10*/cnst31+2/cnst31. *l11* and *l12* define the repeated number of pMODERN basis elements for CH and NH recoupling, respectively. The duration of a pMODERN basis element is 0.4/cnst31. The recoupling times for CH and NH dipolar interactions are *l11*·0.4/cnst31 and *l12*·0.4/cnst31, respectively.

The code

```
;3D (H)CANH with SPEPS element for CA-N transfer and pMODERN for HA-C and N-H recoupling
; basis (H)CANH was developed at CRMN in the group of G. Pintacuda and modified at MPI-NAT in the group of L.Andreas

; Recent notes:
; this version does not require 13C axis inversion

;Avance III version
;parameters:
;p1 : 1H 90 pulse duration
;p3 : 13C 90 pulse duration
;p7 : 15N 90 pulse duration
;p30 : water suppression time (30-200 ms)
;cnst21 : CO offset in ppm (173.7)
;cnst22 : CA offset in ppm (53.7)
;cnst23 : CO/CO offset in ppm (113.7)
;d1 : recycle delay
;d0 : C incremental delay (t1)
;d10 : N incremental delay (t2)
;in10    : 1/2 increment for 15N evolution
;in0   : 1/2 increment for 13CA evolution
;cpdprg1 : tppm (at pl13) or waltz (at pl13)
;cpdprg4 : cwY (at pl12)
;cpdprg5 : cwX (at pl12)
;pcpd1   : pulse length in decoupling sequence (2xtau_r for tppm, 25us for 10kHz waltz)
;pcpd7   : pulse length in decoupling sequence during MODERN
;pl1    : power level of 1H hard pulse
;pl12   : power level of decoupling (10-15 kHz)
;pl13   : power level for water suppression
;spnam1  : 1H shape for 1H->13CA CP (ramp 10-20%)
;spoal1  : N/A
;spoff1 : [ON/RES]
;sp1    : 1H power level during 1H->13CA CP
;spnam10 : 1H shape for 15N->1H(N) CP (ramp 10-20%)
;spoal10  : N/A
;spoff10 : [ON/RES]
;sp10    : 1H power level during 15N->1H CP
;cpdprg2 : 15N decoupling pattern during acq (waltz-16)
;p17     : contact time 15N->1H(N) CP (300-500 us)
;pcpd2   : pulse length in 15N decoupling sequence (25 us)
;pl7    : power level for 15N hard pulse
;pl16   : power level for 15N decoupling (corr. to 10 kHz)
;pl2    : power level for 15N hard pulse
```

```
;pl20   : 15N power level for 15N->1H CP
;spnam2  : 15N shape for 13CA->15N CP (tan-c100-w10pct)
;sp2    : 15N power level for 13CA->15N CP
;spoal2  : N/A
;spoff2 : [ON/RES]
;p15     : contact time 1H->13CA CP
;p18     : Q3 CO pulse duration
;p19     : Q3 CA pulse duration
;pcpd3   : pulse length in 13C decoupling sequence (25 us)
;cpdprg3 : 13CO/CA decoupling pattern during 15N evol (waltz-16)
;pl3    : power level of 13C hard pulse [REFERENCE]
;pl17   : power level for 13CO/CA decoupling (10 kHz)
;spnam9  : 13C shape for 13CA->15N CP (rectangle)
;sp9     : 13C power for 13CA->15N CP
;spoal9  : N/A
;spoff9 : [ON/RES CO]
;spnam18 : 13CO selective pulse shape (Q3)
;spoal18  : N/A
;spoff18 : [ON/RES CO]
;spnam19 : 13CA selective pulse shape (Q3)
;spoal19  : N/A
;spoff19 : [ON/RES CA]
;spnam29 : 13C shape for 1H->13CA CP (rectangle)
;sp29   : 13C power level for 1H->13CA CP
;spoal29  : N/A
;spoff29 : [ON/RES CO]
;zgoptns : -Dfslg, -Dlacq, or blank
;td1 : number of C increments
;td2 : number of N increments

;l21:  Number SPEPS elements
;sp5: ~0.25*MAS C for SPEPS
;sp6: ~0.75*MAS N for SPEPS

;p26 : pulse length in decoupling during MODERN
;p4: pMODERN pule
;pl4: 2.75*MAS pMODERN HC
;pl5: 2.75*MAS pMODERN HN
;l10: number of rotor periods for CT
;l11: loop counter for recoupling time, HC
;l12: loop counter for recoupling time, HN


;$COMMENT=Inverse Cp with INEPT CBCA mixing
;$CLASS=Solids
;$DIM=3D
```

```
;$TYPE=H detect
;$SUBTYPE=Heteronuclear
;$OWNER=CRMN

#include <Avancesolids.incl>

; Start evolutions from exactly 0
"d10=0.0"
"d0=0.0"

; 1H settings
"spoal1=0.5"   ; default value (irrelevant)
"spoff1=0.0"    ; on-resonance
"spoal10=0.5"  ; default value (irrelevant)
"spoff10=0.0"   ; on-resonance

; 15N settings
"pcpd2=25"                        ;does not work!
"plw2=plw7"
"plw16=plw2*(pow(p7/25,2))"     ; 15N waltz 10kHz decoupling power level
"spoal2=0.5"   ; default value (irrelevant)
"spoff2=0.0"     ; on-resonance

; 13C settings
"plw17=plw3*(pow(p3/25,2))"     ; 13C waltz 10kHz decoupling power level

"cnst22 = (sfo3-bf3)*1000000/bf3"  ; CA frequency offset (ppm)
"cnst21 = 173.7"     ; CO frequency offset (ppm)
;"cnst21 = cnst22+(173.7-53.7)"     ; CO frequency offset (ppm)
"cnst23 = cnst22+(113.7-53.7)"    ; the offset half-way CO and CA (ppm)

"p18=3.412/(85.0*bf3/1000000)"    ; 95 ppm bandwidth (safe)
"spw18=plw3*pow((0.5/(p18*0.1515))/(0.25/p3),2)" ;  Q3 power level
"spoal18=0.5"                      ; default value (irrelevant)
"spoff18=bf3*((cnst21-cnst22)/1000000)"

"p19=3.412/(85.0*bf3/1000000)"      ; 95 ppm bandwidth (safe)
"spw19=plw3*pow((0.5/(p19*0.1515))/(0.25/p3),2)" ;  Q3 power level
"spoal19=0.5"                          ; default value (irrelevant)
"spoff19=0.0"                      ; CA frequency

"spoal9=0.0"                      ; needed for offset on C
"spoff9=0.0"                      ; on-resonance

"spoal29=1.0"                      ; needed for offset on C
"spoff29=0.0"                      ; on-resonance CA
```

```
"in0=inf1/2"
"in10=inf2/2"


;"acqt0=0"              ; baseopt correction

"p10 = (16*(2s/cnst31))"

define delay mix
"mix = (l21*p10)"


"plw27 = 0.0"

"p4=(0.1s/cnst31)"
define delay del26
"del26 = (1s/cnst31)-p3-1u"
define delay del27
"del27 = (1s/cnst31)-p7-1u"
define delay mix1
"mix1 = (l11*(4*p4))"
define delay mix2
"mix2 = (l12*(4*p4))"

"p25=l10*(1s/cnst31)"

define delay del21
"del21 = 0.5*p25 - mix1"
define delay del22
"del22 = 0.5*p25"

define delay del23
"del23 = 0.5*p25 - mix2"

1m
  if "p15   > 15m"  goto Problem
  if "aq    > 56m"    goto Problem
  if "p17    > 7m"    goto Problem
  if "mix    > 15m"    goto Problem
  goto PassParams
Problem, 1m
  print " cnst31 is too low; aq, p15 or p17 are too long."
  goto HaltAcqu
PassParams, 1m
```

```
1 ze
  mix
  mix1
  mix2
2 d1 do:f2
#include <p15_prot.incl>
#include <aq_prot.incl>

  ;1u fq=0:f3
  1u fq=cnst22(bf ppm):f3
  (p1 pl1 ph3):f1

  (p15:sp29 ph15):f3 (p15:sp1 ph16):f1

(p3 pl3 ph26):f3
  (p30*0.066 pl13 ph0):f1
  (p30*0.066 pl13 ph1):f1
  (p30*0.066 pl13 ph0):f1
  (p30*0.066 pl13 ph1):f1
(p3 pl3 ph27):f3
;;;;;;;;;;;;;;;;pMODERN;;;;;;;;;;;;;;;;;;;;;;;;;;;;

3 (p4 pl4 ph29^):f1
  (p4 pl4 ph29^):f1
  (p4 pl4 ph29^):f1
  (p4 pl4 ph29^):f1
  lo to 3 times l11
  1u cpds4:f1
  del21
  del26
  (p3*2 pl3 ph0):f3
  del26
  del22
  1u do:f1
  0.5u cpds1:f1
;;;;;;;;;;;;;;;;;;;;;;;;;;;;;;;;;;;;;;;;;;;;;;;

  (p18:sp18 ph2):f3  ;CO selective Pi
  1u
  (p19:sp19 ph2):f3   ;CA selective Pi
  1u
  d0
  (center (p7*2 ph0 pl7):f2 (p18:sp18 ph2):f3)  ;CO selective Pi
  d0
  1u do:f1
```

```
; echo-antiecho determination
(p3 pl3 ph4):f3        ; 1st half of 180
(p3 pl3 ph25):f3        ; 2nd half of 180

5 (p10:sp5 ph10):f3 (p10:sp6 ph12):f2
  lo to 5 times l21
  1u fq=cnst23(bf ppm):f3
  1u cpds1:f1 cpds3:f3
  d10
  d10
  1u do:f1 do:f3

;;;;;;;;;;;;;;;pMODERN;;;;;;;;;;;;;;;;;;;;;;;;;
6 (p4 pl5 ph29^):f1
  (p4 pl5 ph29^):f1
  (p4 pl5 ph29^):f1
  (p4 pl5 ph29^):f1
   lo to 6 times l12
  1u cpds4:f1
  del23
  del27
  (p7*2 pl7 ph18):f2
  del27

  del22
  1u do:f1
;;;;;;;;;;;;;;;;;;;;;;;;;;;;;;;;;;;;;;;;;;;;;;;

  (p7 pl7 ph5):f2
 ;water suppression
  (p30*0.184 pl13 ph0):f1
  (p30*0.184 pl13 ph1):f1
  (p30*0.184 pl13 ph0):f1
  (p30*0.184 pl13 ph1):f1
 ;water suppression
  (p7 pl7 ph6):f2

  (p17:sp20 ph7):f2 (p17:sp10 ph17):f1

  1u cpds2:f2
  go=2 ph31
  1m do:f2

  10m mc #0 to 2

 F1EA(calph(ph25, +180),caldel(d0, +in0) & calph(ph15, +180) & calph(ph31, +180))  ;13C
```

```
F2PH(calph(ph5, +90) & calph(ph18, +90), caldel(d10, +in10)) ;15N


HaltAcqu, 1m      ;jump address for protection files
exit              ;quit

ph0  = 0
ph1  = 1
ph3  = 1
ph15 = 0
ph16 = 0 0 2 2
ph5 =  1
ph6 =  3
ph2 = 0
ph4= 1
ph25= 3
ph10= 0 3
ph12= 0 1
ph21= 0
ph22= 2

ph29 = (360) 124 0 123 180

ph26=1
ph27=3

ph7  = 0 0 0 0 2 2 2 2
ph17 = 0
ph18=0

ph31 = 1 3 3 1 3 1 1 3
```

References

(1) Nimerovsky, E.; Soutar, C. P. A Modification of γ-Encoded RN Symmetry Pulses for Increasing the Scaling Factor and More Accurate Measurements of the Strong Heteronuclear Dipolar Couplings. *J. Magn. Reson.* **2020**, *319*, 106827. https://doi.org/10.1016/j.jmr.2020.106827.

(2) Metz, G.; Wu, X. L.; Smith, S. O. Ramped-Amplitude Cross Polarization in Magic-Angle-Spinning NMR. *J. Magn. Reson. A* **1994**, *110* (2), 219–227. https://doi.org/10.1006/jmra.1994.1208.

(3) Burum, D. P.; Ernst, R. R. Net Polarization Transfer via a *J*-Ordered State for Signal Enhancement of Low-Sensitivity Nuclei. *J. Magn. Reson. 1969* **1980**, *39* (1), 163–168. https://doi.org/10.1016/0022-2364(80)90168-7.

(4) Thakur, R. S.; Kurur, N. D.; Madhu, P. K. Swept-Frequency Two-Pulse Phase Modulation for Heteronuclear Dipolar Decoupling in Solid-State NMR. *Chem. Phys. Lett.* **2006**, *426* (4), 459–463. https://doi.org/10.1016/j.cplett.2006.06.007.

(5) Shaka, A. J.; Keeler, J.; Frenkiel, T.; Freeman, R. An Improved Sequence for Broadband Decoupling: WALTZ-16. *J. Magn. Reson. 1969* **1983**, *52* (2), 335–338. https://doi.org/10.1016/0022-2364(83)90207-X.

(6) Zhou, D. H.; Rienstra, C. M. High-Performance Solvent Suppression for Proton Detected Solid-State NMR. *J. Magn. Reson.* **2008**, *192* (1), 167–172. https://doi.org/10.1016/j.jmr.2008.01.012.

(7) Nimerovsky, E.; Becker, S.; Andreas, L. B. Windowed Cross Polarization at 55 kHz Magic-Angle Spinning. *J. Magn. Reson.* **2023**, *349*, 107404. https://doi.org/10.1016/j.jmr.2023.107404.

(8) Hartmann, S. R.; Hahn, E. L. Nuclear Double Resonance in the Rotating Frame. *Phys. Rev.* **1962**, *128* (5), 2042–2053. https://doi.org/10.1103/PhysRev.128.2042.

(9) Nimerovsky, E.; Varkey, A. C.; Kim, M.; Becker, S.; Andreas, L. B. Simplified Preservation of Equivalent Pathways Spectroscopy. *JACS Au* **2023**, *3* (10), 2763–2771. https://doi.org/10.1021/jacsau.3c00312.

(10) Andreas, L. B.; Reese, M.; Eddy, M. T.; Gelev, V.; Ni, Q. Z.; Miller, E. A.; Emsley, L.; Pintacuda, G.; Chou, J. J.; Griffin, R. G. Structure and Mechanism of the Influenza A M218–60 Dimer of Dimers. *J. Am. Chem. Soc.* **2015**, *137* (47), 14877–14886. https://doi.org/10.1021/jacs.5b04802.

(11) Mehring, M. *Principles of High Resolution NMR in Solids*, 2nd ed.; Springer-Verlag: Berlin Heidelberg, 1983. https://doi.org/10.1007/978-3-642-68756-3.

(12) Xue, K.; Nimerovsky, E.; Tekwani Movellan, K. A.; Becker, S.; Andreas, L. B. Backbone Torsion Angle Determination Using Proton Detected Magic-Angle Spinning Nuclear Magnetic Resonance. *J. Phys. Chem. Lett.* **2022**, *13* (1), 18–24. https://doi.org/10.1021/acs.jpclett.1c03267.

(13) Nimerovsky, E.; Goldbourt, A. Insights into the Spin Dynamics of a Large Anisotropy Spin Subjected to Long-Pulse Irradiation under a Modified REDOR Experiment. *J. Magn. Reson.* **2012**, *225*, 130–141. https://doi.org/10.1016/j.jmr.2012.09.015.

(14) Pauli, J.; van Rossum, B.; Förster, H.; de Groot, H. J. M.; Oschkinat, H. Sample Optimization and Identification of Signal Patterns of Amino Acid Side Chains in 2D RFDR Spectra of the α-Spectrin SH3 Domain. *J. Magn. Reson.* **2000**, *143* (2), 411–416. https://doi.org/10.1006/jmre.2000.2029.

(15) Chevelkov, V.; Faelber, K.; Schrey, A.; Rehbein, K.; Diehl, A.; Reif, B. Differential Line Broadening in MAS Solid-State NMR Due to Dynamic Interference. *J. Am. Chem. Soc.* **2007**, *129* (33), 10195–10200. https://doi.org/10.1021/ja072024c.
(16) Schnell, J. R.; Chou, J. J. Structure and Mechanism of the M2 Proton Channel of Influenza A Virus. *Nature* **2008**, *451* (7178), 591–595. https://doi.org/10.1038/nature06531.
(17) Andreas, L. B.; Eddy, M. T.; Pielak, R. M.; Chou, J.; Griffin, R. G. Magic Angle Spinning NMR Investigation of Influenza A M218−60: Support for an Allosteric Mechanism of Inhibition. *J. Am. Chem. Soc.* **2010**, *132* (32), 10958–10960. https://doi.org/10.1021/ja101537p.
(18) Hohwy, M.; Jaroniec, C. P.; Reif, B.; Rienstra, C. M.; Griffin, R. G. Local Structure and Relaxation in Solid-State NMR: Accurate Measurement of Amide N−H Bond Lengths and H−N−H Bond Angles. *J. Am. Chem. Soc.* **2000**, *122* (13), 3218–3219. https://doi.org/10.1021/ja9913737.
(19) Nimerovsky, E.; Kosteletos, S.; Lange, S.; Becker, S.; Lange, A.; Andreas, L. B. Homonuclear Simplified Preservation of Equivalent Pathways Spectroscopy. *J. Phys. Chem. Lett.* **2024**, 6272–6278. https://doi.org/10.1021/acs.jpclett.4c00991.
